# A two-level model for the role of complex and young genes in the formation of organism complexity and new insights into the relationship between evolution and development

**DOI:** 10.1186/s13227-018-0111-4

**Published:** 2018-11-12

**Authors:** Dong Yang, Aishi Xu, Pan Shen, Chao Gao, Jiayin Zang, Chen Qiu, Hongsheng Ouyang, Ying Jiang, Fuchu He

**Affiliations:** 1 State Key Laboratory of Proteomics, Beijing Proteome Research Center, National Center for Protein Sciences (Beijing), Beijing Institute of Lifeomics, Beijing, 102206 The People’s Republic of China; 2 Animal Sciences College of Jilin University, Changchun, 130062 The People’s Republic of China

**Keywords:** Genome complexity, Gene complexity, Gene age grade, Evolution, Development, Organism complexity

## Abstract

**Background:**

How genome complexity affects organismal phenotypic complexity is a fundamental question in evolutionary developmental biology. Previous studies proposed various contributing factors of genome complexity and tried to find the connection between genomic complexity and organism complexity. However, a general model to answer this question is lacking. Here, we introduce a ‘two-level’ model for the realization of genome complexity at phenotypic level.

**Results:**

Five representative species across Protostomia and Deuterostomia were involved in this study. The intrinsic gene properties contributing to genome complexity were classified into two generalized groups: the complexity and age degree of both protein-coding and noncoding genes. We found that young genes tend to be simpler; however, the mid-age genes, rather than the oldest genes, show the highest proportion of high complexity. Complex genes tend to be utilized preferentially in each stage of embryonic development, with maximum representation during the late stage of organogenesis. This trend is mainly attributed to mid-age complex genes. In contrast, young genes tend to be expressed in specific spatiotemporal states. An obvious correlation between the time point of the change in over- and under-representation and the order of gene age was observed, which supports the funnel-like model of the conservation pattern of development. In addition, we found some probable causes for the seemingly contradictory ‘funnel-like’ or ‘hourglass’ model.

**Conclusions:**

These results indicate that complex and young genes contribute to organismal complexity at two different levels: Complex genes contribute to the complexity of individual proteomes in certain states, whereas young genes contribute to the diversity of proteomes in different spatiotemporal states. This conclusion is valid across the five species investigated, indicating it is a conserved model across Protostomia and Deuterostomia. The results in this study also support ‘funnel-like model’ from a new viewpoint and explain why there are different evo–devo relation models.

**Electronic supplementary material:**

The online version of this article (10.1186/s13227-018-0111-4) contains supplementary material, which is available to authorized users.

## Background

The relationship between genome complexity and organism complexity is one of the core topics in genomics and evolutionary systems biology. However, measuring genome complexity is not a simple task. Gene numbers show no obvious correlation with organism complexity, a phenomenon referred to as the *G*-value paradox [1–3]. To explain this paradox, biologists have separately presented numerous genome complexity factors [3–9] related to genome sequence and the structural and functional features of genes and their products. However, as each study focuses on one or only a few factors, one cannot obtain a global understanding of the factors contributing to genome complexity. Additionally, the various contributing factors are uncategorized, further complicating and confusing their relationships.

In this study, the intrinsic properties of genes/gene products related to genome complexity were categorized into two classes: gene complexity and gene age grade. And we try to answer the fundamental question about how the complex and young genes contribute to the formation of organism complexity. It is through the process of development that genome complexity is represented as phenotypic complexity at the organismal level [10]. Development is a complex and dynamic process involving differentiation from a single embryonic stem cell to various terminal differentiated somatic cells. During this process, organism complexity gradually increases because of an increase in the number of cell types constituting the whole body [11–13]. At the adult stage, organism complexity is maintained by specific gene expression patterns among different organs, tissues and cell types (OTCs) of the adult body. The complexity of the organism is determined at two levels. The first level comprises the diversity of spatiotemporal states, i.e., different specific developmental time points or OTCs. If an organism contains much more different OTCs, it can be regarded as a more complex organism. The second level is the complexity of each spatiotemporal state. If two organisms have the same number of OTCs, the difference in complexity between them is determined by the complexity of each individual OTC in each organism. To explore how these genomic complexity-contributing factors affect an organism’s phenotypic complexity, both levels should be considered simultaneously.

We classified genes, including protein-coding genes (PCGs) and miRNA genes, according to the above-mentioned genome complexity factors, and explored the relationship between gene complexity and age degree. We then investigated the over- and under-representation of each class of genes in a certain developmental stage/OTC (based on the gene expression data listed in Additional file 1: Table S1) compared with all the genes in the genome. In addition, we calculated the tissue-/stage-specificity and compared this across each class of genes. Based on these results, a general pattern for the utilization of the genome complexity factors was inferred.

In addition, the relationship between gene age grade and the expression pattern during development may provide new clues to the understanding of the relationship between evolution and development. In this filed, there are two major models related to evolution–development connection. The first one is the ‘funnel-like’ model, which was firstly proposed by von Baer in 1828 [14]. In this model, developmental similarities are highest in the earliest stages of embryogenesis and lowest at the end of development. Some recent studies also supported this model based on the analysis of genome-wide gene expression [15, 16]. In 1994, Duboule proposed another model [17], the ‘hourglass’ model, in which the middle stage of embryonic development show the most conserved morphological pattern, but not the early stage. During this middle stage, the body plan is being set. This model was also supported by the high-throughput gene expression analysis [15, 18–21]. Based on our results, we obtained some new insights into this fundamental question and the probable explanations for the ‘paradox’ between these two models.

## Results

### The classification of the factors contributing to genome complexity

**Fig. 1:**
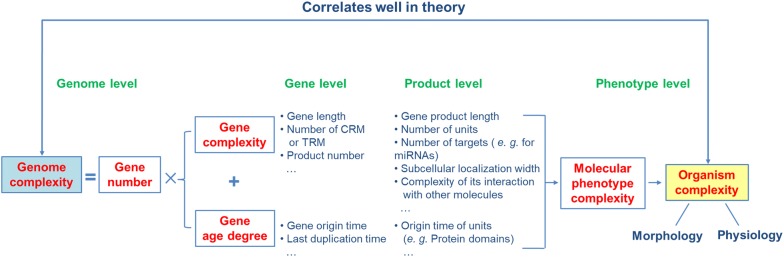
Schematic diagram of the factors contributing to genome/organism complexity. In theory, organism complexity correlates well with genome complexity. The factors contributing to genome complexity are classified into two classes: gene number and intrinsic gene properties which include the complexity and age degree of genes or the gene products. *CRM* cis-regulatory motifs/modules, *TRM* trans-regulatory molecules

To give a clear and general conception of the factors contributing to genome complexity, we categorized them into two classes for the first time (Fig. 1). The first class is the number of genes and non-genic elements. The gene number is the same as the original understanding of genome complexity. The second class is the intrinsic properties of the genes or non-genic elements. In this study, due to the data available, we mainly focus on the intrinsic properties of genes, which include two generalized categories: gene complexity and gene age degree. Gene complexity factors include the gene length, number of *cis*-regulatory motifs and trans-regulatory molecules [3], and the complexity features of the gene’s product, such as the length [6], the number of structural or functional units (e.g., protein domains) [5, 6], the number of subcellular locations and the complexity of its interaction with other molecules. The factors for gene age degree include the age of the genes and its protein domains, the last duplication time of the genes. The young genes contribute substantially to the novel morphological and functional characteristics of complex organisms during evolution [22–29].

### The relationship between gene complexity and age degree

In this study, gene complexity and age degree were defined as the two basic properties of a gene. Gene complexity refers to the complexity of the sequence, structure and function of one gene, whereas gene age degree refers to the evolutionary novelty of one gene in a certain species compared with other species across the phylogenetic tree. Both of them contribute to genome complexity. Four factors were selected to represent the complexity of a gene: gene length (GL), *cis*-regulatory module number (CRMN) [6, 30], protein length (PL) [6] and domain number including repeats in a protein (DNIR) [5, 6] (see Additional file 2: Table S2 for the detailed values for each gene). We confirmed that these gene complexity factors correlate well with organism complexity, measured with cell type number within the organism [2, 31]. In total, 46 eukaryotes were used for this analysis (Additional file 1: Fig. S1a–c, Table S3).

To explore the role of complex genes in the formation of organism complexity, we then investigated the functional characteristics of complex genes. Compared with the simple genes, the complex genes tended to take part in developmental and multicellular processes (Additional file 1: Fig. S1d). Interestingly, certain regulatory processes, such as signal transduction, were over-represented in simple proteins in the mouse data, due in part to the olfactory receptor family, a large family with special expression and function characteristics [32]. Among the 870 short genes (≤ 3200 bp) participating in signal transduction, 693 were olfactory receptor-encoding genes (Additional file 3: Table S4). In view of knockout phenotype of genes, we found that complex genes tended to have multiple knockout phenotypes (Additional file 1: Fig. S1e1–e4 for mouse data, and Additional file 4: Table S5 for other species). Regarding pathways, the complex genes tended to be involved in multiple KEGG pathways. Almost half of the high-complexity genes of mouse participated in two or more KEGG pathways (Additional file 1: Fig. S1f1–f4). We further analyzed what pathways are over-represented in complex genes compared with all the genes participating in at least one pathway. Most of these pathways are signaling pathways, such as the MAPK, calcium, ErbB, insulin, Wnt and TGF-β signaling pathways (Additional file 5: Table S6).

One may expect the above-mentioned four complexity factors to show interdependence. For example, long proteins tend to contain multiple domains. To test the independence of these four complexity factors, the spearman correlations between each pair of them in the five species were calculated (Table 1). In fact, the correlations were not so strong. Taking mouse data as an example, the strongest correlation, between GL and PL, showed a coefficient of only 0.58, and the weakest, between DNIR and CRMN, showed 0.17. To further explore the features of the complex genes common or specific to these four factors, Venn diagrams of the four categories of complex genes were used to visualize their detailed relationship (Additional file 1: Fig. S2a). As a result, about 22.8–41.8% complex genes only belong to one category of the complex genes (Additional file 1: Fig. S2b), and they have distinct specific functional features (see supplementary results and Figure s2c in Additional file 1 for details). These results indicate that these four factors are to some extent complementary, and it would therefore be insufficient to measure gene complexity using only one of them.

**Table 1:** Results of the spearman correlation analysis for each pair of gene complexity and age factors of mouse

Factor pairs	*M. musculus*		*G. gallus*		*D. rerio*		*D. melanogaster*		*C. elegans*	
	*R*	*P*	*R*	*P*	*R*	*P*	*R*	*P*	*R*	*P*
GL vs. PL	0.58	< 1E−323	0.57	< 1E−323	0.58	< 1E−323	0.79	< 1E−323	0.80	< 1E−323
GL vs. CRMN	0.46	< 1E−323	0.20	2.6E−126	0.04	4.4E−10	0.13	2.5E−50	− 0.12	1.3E−63
GL vs. DNIR	0.33	< 1E−323	0.34	< 1E−323	0.36	< 1E−323	0.42	< 1E−323	0.38	< 1E−323
PL vs. CRMN	0.27	< 1E−323	0.05	2.4E−10	0.01	2.4E−02	0.04	2.2E−07	− 0.09	6.1E−39
PL vs. DNIR	0.52	< 1E−323	0.53	< 1E−323	0.50	< 1E−323	0.48	< 1E−323	0.49	< 1E−323
CRMN vs. DNIR	0.17	3.0E−138	0.01	2.8E−01	0.01	1.7E−01	0.11	3.3E−41	− 0.03	3.9E−05
GOT_Ens vs. GOT_Mode	0.64	< 1E−323	0.47	< 1E−323	0.47	< 1E−323	0.74	< 1E−323	0.64	< 1E−323
GOT_Ens vs. LDT	0.65	< 1E−323	0.52	< 1E−323	0.42	< 1E−323	0.76	< 1E−323	0.87	< 1E−323
GOT_Ens vs. DOT	0.60	< 1E−323	0.51	< 1E−323	0.45	< 1E−323	0.64	< 1E−323	0.67	< 1E−323
GOT_Mode vs. LDT	0.67	< 1E−323	0.37	< 1E−323	0.35	< 1E−323	0.57	< 1E−323	0.50	< 1E−323
GOT_Mode vs. DOT	0.87	< 1E−323	0.39	< 1E−323	0.43	< 1E−323	0.67	< 1E−323	0.60	< 1E−323
LDT vs. DOT	0.40	< 1E−323	0.32	< 1E−323	0.22	2.9E−280	0.48	< 1E−323	0.56	< 1E−323
GL vs. GOT_Ens	− 0.40	< 1E−323	− 0.23	7.9E−175	− 0.30	< 1E−323	− 0.27	1.6E−230	− 0.34	< 1E−323
GL vs. GOT_Mode	− 0.28	< 1E−323	− 0.16	3.3E−83	− 0.17	1.3E−168	− 0.23	8.0E−165	− 0.27	< 1E−323
GL vs. LDT	− 0.44	< 1E−323	− 0.16	4.2E−80	− 0.25	< 1E−323	− 0.29	7.6E−262	− 0.30	< 1E−323
GL vs. DOT	− 0.22	< 1E−323	− 0.06	7.7E−12	− 0.08	2.7E−38	− 0.19	8.0E−115	− 0.32	< 1E−323
PL vs. GOT_Ens	− 0.35	< 1E−323	− 0.25	8.5E−209	− 0.20	2.2E−230	− 0.32	5.6E−321	− 0.41	< 1E−323
PL vs. GOT_Mode	− 0.20	2.0E−175	− 0.14	1.0E−63	− 0.10	2.2E−59	− 0.30	8.3E−278	− 0.32	< 1E−323
PL vs. LDT	− 0.34	< 1E−323	− 0.17	1.6E−97	− 0.13	2.8E−103	− 0.32	< 1E−323	− 0.38	< 1E−323
PL vs. DOT	− 0.21	2.0E−213	− 0.09	1.1E−27	− 0.06	3.4E−23	− 0.23	5.2E−164	− 0.40	< 1E−323
CRMN vs. GOT_Ens	− 0.27	< 1E−323	0.06	1.1E−14	− 0.03	2.3E−06	− 0.13	7.7E−54	0.01	3.4E−01
CRMN vs. GOT_Mode	− 0.13	2.0E−78	− 0.01	4.5E−01	− 0.04	1.7E−09	− 0.10	1.4E−34	− 0.04	7.6E−10
CRMN vs. LDT	− 0.31	< 1E−323	0.04	3.9E−06	− 0.04	6.8E−10	− 0.18	1.1E−106	0.01	5.6E−02
CRMN vs. DOT	− 0.17	< 1E−323	0.04	1.9E−07	− 0.02	6.7E−04	− 0.08	3.5E−19	− 0.01	3.6E−01
DNIR vs. GOT_Ens	− 0.34	< 1E−323	− 0.30	5.6E−302	− 0.25	< 1E−323	− 0.57	< 1E−323	− 0.61	< 1E−323
DNIR vs. GOT_Mode	− 0.20	2.0E−173	− 0.18	5.7E−103	− 0.19	7.1E−207	− 0.50	< 1E−323	− 0.45	< 1E−323
DNIR vs. LDT	− 0.22	6.0E−240	− 0.14	2.2E−68	− 0.10	3.5E−61	− 0.49	< 1E−323	− 0.55	< 1E−323
DNIR vs. DOT	− 0.36	< 1E−323	− 0.31	1.2E−312	− 0.32	< 1E−323	− 0.61	< 1E−323	− 0.79	< 1E−323

The gene complexity factors used in this study included GL (gene length), CRMN (*cis*-regulatory module number), PL (protein length) and DNIR (domain number including repeats in one protein). The gene age factors included GOT (gene origin time), LDT (last duplication time) and DOT (domain origin time). Correlation coefficients (R) and *P* values are shown in the table

Three factors were used to represent the age degree of a given gene: gene origin time (GOT) [22, 33], last duplication time (LDT) [25, 26] and protein domain origin time (DOT) [5] (Additional file 2: Table S2). Two types of GOT were obtained from EnsemblCompara [34] with slight correction (see “Methods” for detailed information) and a consensus gene age dataset [33] named as GOT_Ens and GOT_Mode, respectively. Similarly, the spearman correlation between each pair of these three novelty factors was calculated (Table 1). Taking mouse data as an example, the correlation was strongest between GOT_Mode and DOT (*R* = 0.87), but weakest coefficient (between DOT and LDT) was only 0.4. Venn diagrams of the three classes of young genes were also used to visualize their detailed relationship (Additional file 1: Fig. S2d). As a result, there are about 20.6–46.5% category-specific young genes (Additional file 1: Fig. S2e), and they also have distinct specific functional features (see supplementary results and Fig. S2f in Additional file 1 for details). These results indicated that the three age degree factors are also complementary to each other to some extent.

These four complexity factors (GL, CRMN, PL and DNIR) and four age degree factors (GOT_Ens, GOT_Mode, LDT and DOT) were used in the further analyses. First, we focused on the relationship between gene complexity and age degree, the two basic intrinsic properties of one gene, and found that nearly all of the complexity factors correlated negatively with the age degree factors; that is, the older genes tend to be more complex, whereas the younger genes tend to be simpler (Table 1). This comprehensive analysis, using multiple factors of gene complexity and age degree, confirmed the previous conclusions about the relationship between gene age and protein length [35, 36]. Our results suggest that this kind of correlation is valid across each pair factor of gene complexity and age degree and valid across the representative species.

**Fig. 2:**
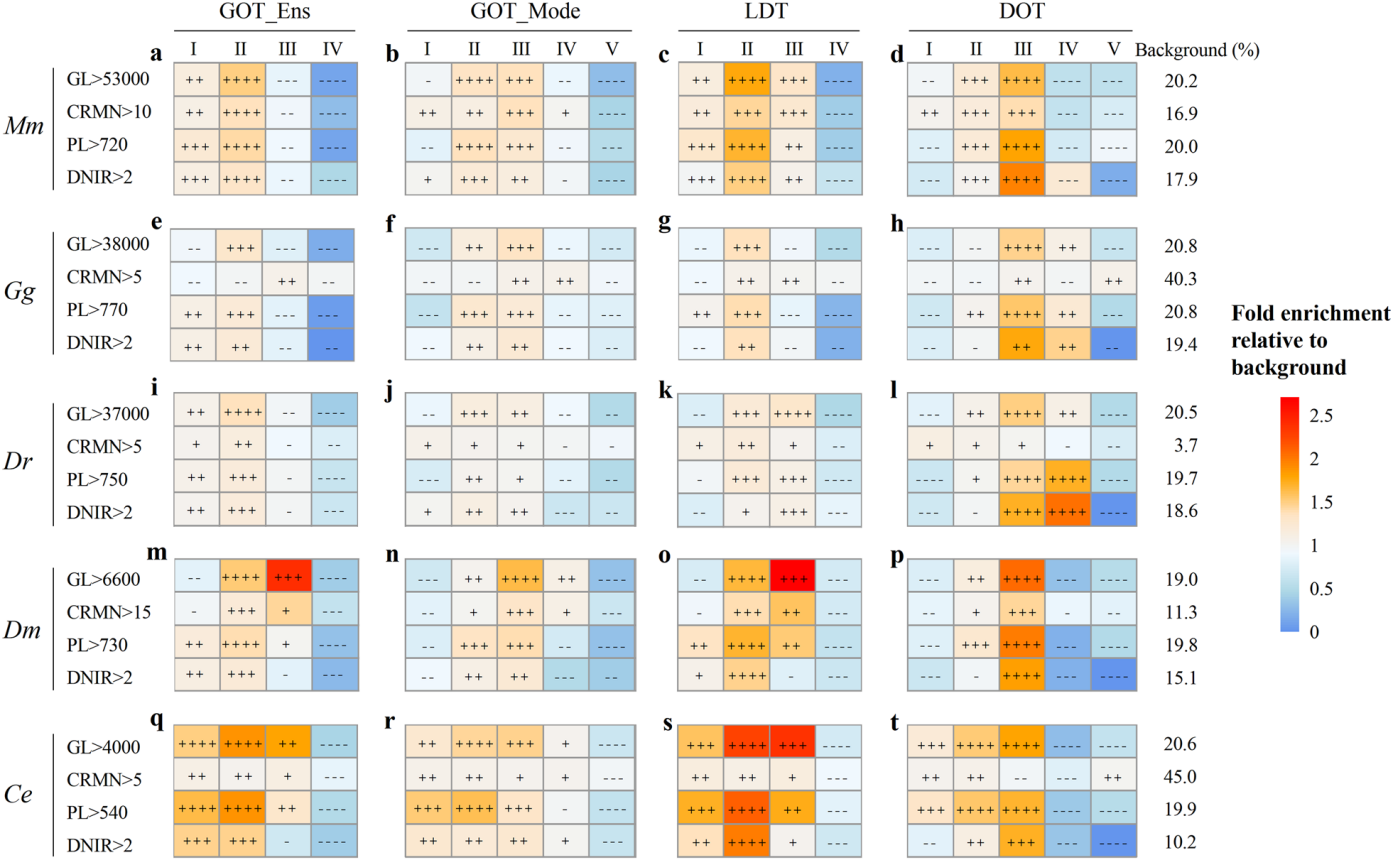
Distribution of complex genes across different age categories in the five species. **a**–**d** for *Mm*, *Mus musculus*; **e**–**h** for *Gg*, *Gallus gallus*; **i**–**l** for *Dr*, *Danio rerio*; **m**–**p** for *Dm*, *Drosophila melanogaster*; **q**–**t** for *Ce*, *Caenorhabditis elegan*s. The percentages of complex protein-coding genes (PCGs) in each age degree category were calculated and divided by the expected percentage. Heat map showing the fold enrichment values obtained from this division. The expected percentage was the percentage of each type of complex PCGs in the genome of each species, represented as ‘background (%)’ in the right region of the figure. Gene complexity was measured by gene length (GL), cis-regulatory module number (CRMN), protein length (PL) and domain number including repeats in one protein (DNIR) for each species, and the results of the most complex PCGs are shown in the figure. The full result data, including other complexity degrees, are in Additional file 1: Table S8. The abbreviations of age degree names: GOT_Mode, gene origin time from the consensus mode gene age dataset, GOT_Ens, gene origin time from the EnsemblCompara database; LDT, last duplication time; and DOT, the origin time of the youngest domain in one protein. The abbreviations of the grades for each age type of each species are listed in Additional file 1: Table S7. For the convenience of presentation, the V grades of the LDT and DOT of *Mus musculus* were the combination of V and VI grades shown in Additional file 1: Table S7. The over- or under-representation strengths of the complex genes in each age degree category were estimated and are represented by − log (*p*) or log (*p*), respectively (see “Methods”). All of the PCGs in each species were used as the background in the over-/under- representation analysis. The symbols in this figure: ++++ , over-represented and *P *< 1E−50; +++, over-represented and 1E−50 ≤ *P* < 1E−10; ++, over-represented and 1E−10 ≤ *P* < 0.05; +, over-represented but *P* > 0.05; −−−− , under-represented and *P* < 1E−10; −−−, under-represented and 1E−50 ≤ *P* < 10-10; −−, under-represented and 10-10 ≤ *P* < 0.05; −, under-represented but *P* > 0.05

To explore the detailed relationship between them, all of the genes were partitioned into several classes according to each gene complexity factor and each age degree factor. Based on GOT_Ens and GOT_Mode, all of the PCGs were classified into four (Fig. 2a, e, i, m, q) or five (Fig. 2b, f, j, n, r) age classes (see Additional file 1: Table S7 for the names of age grades). According to the four gene complexity factors, GL, CRMN, PL and DNIR, all of the PCGs were divided into five or four complexity categories, respectively. The proportion of the most complex PCGs in each age degree category were calculated and divided by the expected percentage (background), which was the percentage of each type of complex PCGs in the genome of each species (Fig. 2, Additional file 6: Table S8). Interestingly, mid-age genes, e.g., the genes originating from the common ancestor of Bilateria (Fig. 2a) or Eumetazoa (Fig. 2b), have the largest proportion of high-complexity genes. The analyses based on other 4 representative species revealed the same conclusion (Fig. 2e, f, i, j, m, n, q, r). We assumed this phenomenon is the outcome of the balance of two different trends (Additional file 1: Fig. S3). In the first trend, one gene became increasingly complex during evolution, so the older genes are more complex, whereas the life spans of the younger genes are too short to become complex; in the second trend, complex organisms produced new complex genes. For example, during the emergence of Eumetazoa, in particular the emergence of Bilateria, probably many new complex genes emerged to meet the complex function requirements, such as the development of three germ layers.

All of the PCGs were then partitioned into several categories according to the last duplication time (LDT, Fig. 2c, g, k, o, s) and protein domain origin time (DOT, Fig. 2d, h, l, p, t), and the proportion of the most complex genes in each category was then compared. The genes with mid-age LDTs (grades II or III) and the genes encoding mid-age protein domains (grade III) have the highest proportion of complex genes. The results were similar to the conclusion that the mid-age genes showed the highest proportion of complex genes mentioned above.

Besides protein-coding genes, noncoding regions also contribute substantially to genome complexity and organism complexity [9, 37]. Thus, the utilization of complex and young noncoding RNA genes was also explored. So far, only miRNA genes were involved in our analysis because of the lack of enough data of the complexity and gene age degree of other noncoding RNA genes. miRNA mainly functions in RNA silencing and posttranscriptional regulation of gene expression [38, 39]. The functional complexity of a miRNA gene is mainly determined by the complexity of the regulatory network in which the miRNA targets to various mRNAs. The number of targets of a certain miRNA is used to represent the complexity degree of a miRNA gene. The age of a miRNA gene is inferred from the miRNA family database (miFam.dat) in miRbase. The relationship between the age grade and the number of targets of a miRNA was investigated. As a result, the mid-age miRNAs, which originated from the common ancestor of mammalian, have the highest proportion of the most complex miRNAs (Additional file 1: Fig. S4). This result is consistent with the conclusion from protein-coding genes, indicating the trend that mid-age genes have the highest proportion of complex genes is a general trend across different types of genes.

To further explore the different roles of complex genes with different age degrees in the formation of organism complexity, we then focused on the functional differences among them. Taking mouse data as an example (Additional file 1: Fig. S5a), the old complex genes generally took part in critical and primitive cellular processes, such as basic metabolism, DNA replication, RNA processing, protein translation, oxidation reduction and transport. The complex genes of medium age (grades II and III) took part mainly in biological processes specific to multicellular organisms, such as development, signal transduction, cell communication, growth, cell motility. The young complex genes were mainly over-represented in transcription regulation. In the view of knockout phenotype of genes, we found that the Bilateria-specific (grade II) complex genes tended to have multiple knockout phenotypes (Additional file 1: Fig. S5b1–b4). As the number of knockout phenotypes of a gene can approximately represent the gene’s functional complexity, the Bilateria-specific complex genes showed a stronger multifunctionality or pleiotropy trend. Regarding pathways, the Bilateria-specific complex genes had the highest proportion of the genes involved in multiple pathways (Additional file 1: Fig. S5c1–c4). These results indicated that compared with other genes, the complex genes with medium age tend to facilitate the formation of organism complexity.

### General utilization patterns of gene complexity/novelty factors in certain spatiotemporal states

Both of the complex and young genes contribute to complexity at genome and organism levels. Along with the selective expression of complex and young genes, both factors can be represented at the phenotypic level. Here, we focused on the general utilization patterns of the gene complexity/age factors in different stages of development and different adult OTCs. We classified PCGs into several categories according to the preceding parameters and calculated the over- or under-representation strength of each gene category in each developmental stage and each adult OTC.

**Fig. 3:**
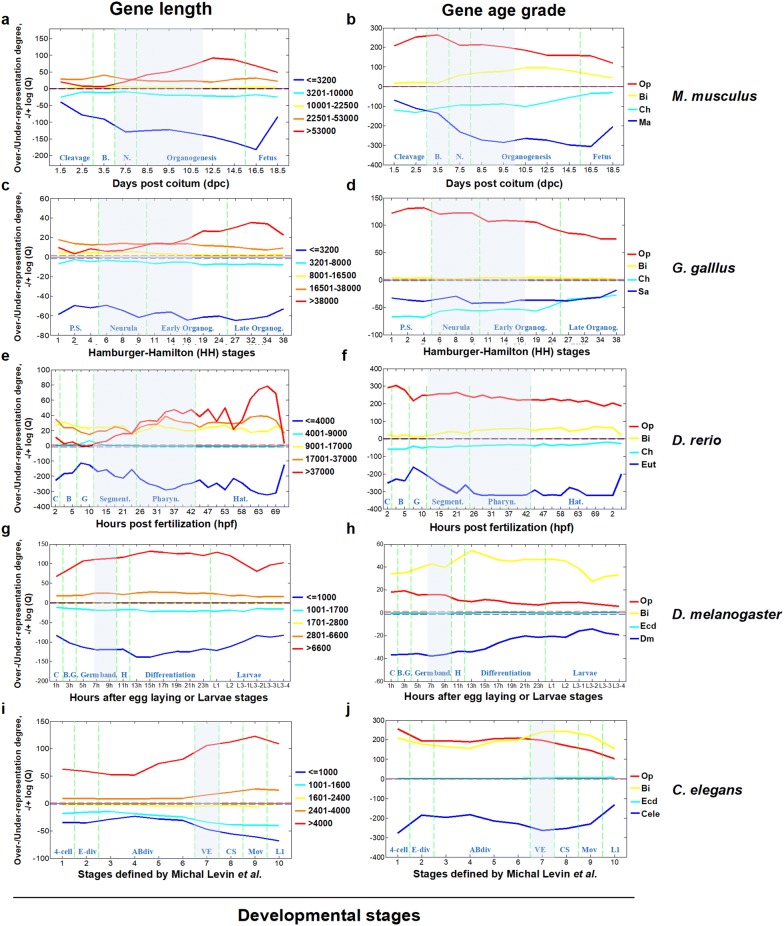
Over- or under-representation strengths of each gene category classified by gene length (**a**, **c**, **e**, **g**, **i**) or gene age grade (**b**, **d**, **f**, **h**,** j**) during development of the five species. Over- and under-representation are represented by −log(*P*) or log(*P*), respectively (see “Methods” for details). The red/blue dashed line represents the ∓log(*P*) value corresponding to significant over- or under-representation. The gray shaded area represents the presumptive phylotypic phase. PCG refers to ‘protein-coding gene’. Developmental stages are separated by dashed light green lines and marked at the bottom of each panel. For *M. musculus*: Cleavage, Blastula (B), Neurula (N), Organogenesis and Fetus (F). For *G. gallus*: Primitive streak (P.S.), Neurula, early Organogenesis (Organog.) and late Organogenesis (Organog.). For *D. rerio* Cleavage (C), Blastula (B), Gastrula (G), Segmentation (Segment.), Pharyngula (Pharyn.) and Hatching (Hat.). For *Drosophila melanogaster*: Cleavage (C), Blastoderm + Gastrulation (B.G.), Germ band elongation and retraction (Germ band.), Early of head involution (H), Differentiation and Larvae. For *C. elegans*: 4-cell stage, E-cell division (E-div), division of the AB lineage (ABdiv), Ventral Enclosure (VE), Comma Stage (CS), Movement (Mov) and First stage larva (L1). The abbreviations for gene age grades: Op, Opisthokonta; Bi, Bilateria; Ch, Chordata; Ma, Mammalia; Eut, Euteleostomi; Ecd, Ecdysozoa; Dm, *D. melanogaster*; Cele, *C. elegans*

Based on gene length (GL, Fig. 3a, c, e, g, i), all of the PCGs were classified into one of the five complexity categories. We found that complex genes were significantly over-represented in nearly all of the stages of development of the five species, increasing from the beginning of the phylotypic stage, a developmental phase during which the embryonic morphology of all species within a phylum is particularly similar [10, 15, 19, 21, 40]. The over-representation peaked at the late stage of the organogenesis stage (for example, E14.5 in mouse) and then decreased a little. The dynamic trend of under-representation of simple genes (low complexity) was similar to the over-representation of the complex genes (Fig. 3a, c, e, g, i). Similar results can be obtained from the analysis of other 3 complexity factors, CRMN, PL and DNIR (Additional file 1: Fig. S6). These results indicated that complex genes are utilized preferentially at each time point of embryonic development, contributing to the complexity of each state. In particular, the complex genes are much more over-represented during the middle and late stages, which substantially contributes to the increasing complexity of the embryo.

For gene age degree, based on GOT, LDT and DOT, the PCGs were divided into several classes (Fig. 3b, d, f, h, j, Additional file 1: Fig. S7). It is obvious that the old genes are significantly over-represented during the entire process of embryonic development, particularly in the early stages, while the young genes are under-represented in all stages. Regardless of whether gene age degree was represented by GOT, LDT or DOT, similar conclusions were obtained (see supplementary description in Additional file 1 for more DOT-related results). In addition, we found an interesting species-specific phenomenon. During embryonic development of *D. melanogaster* and *C. elegans*, the over-representation strength of the sub-old age genes (Bilateria grade) was stronger than the oldest genes (Opisthokonta) (Fig. 3h, j). This trend is different from other 3 species (Fig. 3b, d, f), suggesting the relatively higher-level requirement of the bilaterian-specific genes in the embryonic development of Protostomia.

Interestingly, there is an obvious correlation between the time point change of the over- or under-representation strength and the order of gene origin time (Fig. 3b, d, f, h, j, and Additional file 1: Fig. S7). The over-representation of old genes peaked in the early stages (e.g., E3.5 of mouse), whereas that of the mid-age genes peaked during the late stages of organogenesis (E10.5–E12.5 for mouse). In contrast, the under-representation of both the two classes of young genes decreased. As an example, the Chordata-Amniota grade genes of mouse decreased in the early stages, and the Mammalia-Mus grade genes decreased in the later stages. These results were consistent with the ‘funnel-like’ model of the conservation pattern of development [15, 16], which predicts conservation at the earliest embryonic stage (see “Discussion” section for details).

Both complex and young genes contribute to genome complexity. To more comprehensively describe the regular utilization pattern of genomic complexity factors, we combined gene complexity and gene age degree to classify genes. Taking mouse data as an example, all of the old genes (grade I, Opisthokonta-specific), regardless of their degree of complexity, were over-represented across the developmental stages (Additional file 1: Fig. S8a, e, i, m). Most of these old genes showed the greatest over-representation in the early stages, except for the high-complexity genes, which were most over-represented in the middle stage of organogenesis. The Bilateria-specific (grade II) complex genes became increasingly over-represented from the beginning of the phylotypic stage [15, 21] and peaked at E12.5–14.5 (Additional file 1: Fig. S8b, f, j, n), similar to the complex genes from all of the PCGs (Fig. 3a, b, c, d). This implied that the trend of the dynamic over-representation strength of all PCGs occurred mainly due to Bilateria-specific complex genes. The Chordata-specific (grade III) complex genes were under-represented in the early stages of development (Additional file 1: Fig. S8c, g, k, o), being the least abundant from the beginning of the phylotypic stage, but they became over-represented during the later stages. All of the Mammalia-specific (grade IV) genes, regardless of their degree of complexity, were under-represented across the developmental stages (Additional file 1: Fig. S8d, h, l, p). From these results, we inferred that gene age degree was more powerful than gene complexity in determining of the strength of over- or under-representation.

The preceding analyses were based on developmental data for the whole embryo. When the same analyses were performed using the developmental data for four organs, including the brain, liver, heart and lung of mouse, the general trends were also observed during the development of specific organs (Additional file 1: Fig. S9, Fig. S10). However, only during brain development did we observe the same trend of increasing over-representation of complex genes (Additional file 1: Fig. S9). This implied the increasing brain complexity during development from the moment of its formation is much more obvious than the other organs investigated. Notably, in the liver, the over-representation of complex genes reduced (Additional file 1: Fig. S9b, f, j, n), perhaps due to the emigration of the hematopoietic system from the fetal liver in the later stages of embryonic development. In addition, the general utilization patterns of the complex and young genes in certain adult OTCs were the same as those for the embryonic development data (see supplementary results and figure s11 in Additional file 1 for details).

As for the utilization of complex and young miRNA genes, we found that the gene age degree also was more powerful than gene complexity in determining the strength of over- or under-representation. Old miRNA genes (grades I and II, Metazoa-specific and Vertebrata-specific) were significantly over-represented in each state (Additional file 1: Fig. S12a, d), whereas young miRNA genes (grades III, IV and V, i.e., Mammalia-specific, Rodentia-specific and Mus-specific, respectively) were significantly under-represented in most of the states examined (Additional file 1: Fig. S12a, d). However, there is not such a trend that complex miRNA genes (with more target genes) are over-represented (Additional file 1: Fig. S12a, d) as the protein-coding genes (Fig. 3, Additional file 1: Fig. S11). This result implies that at a certain state the miRNA genes with more target genes are not utilized preferentially to form complexity and they may function at different states.

### Gene complexity, novelty and spatiotemporal specificity

The preceding result that young genes tend to be under-represented during development indicates that young genes are not utilized preferentially in certain states. If so, how do they contribute to the biological complexity at the organism level? The following developmental stage-specificity and OTC-specificity analyses may answer this question.

**Fig. 4:**
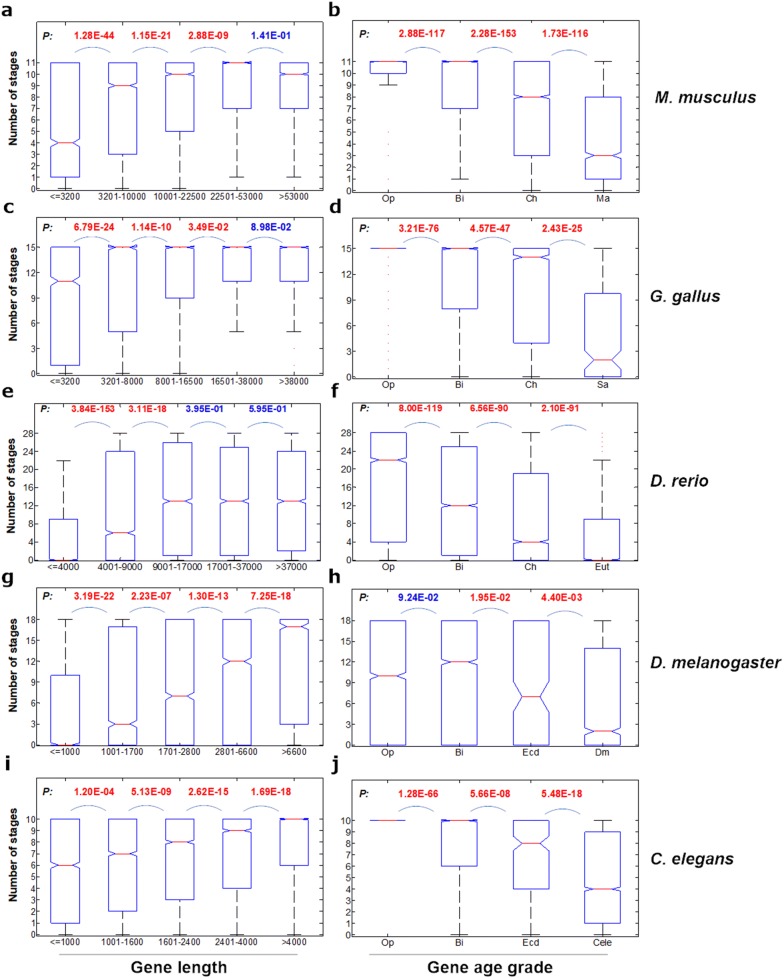
Developmental stage-specificity of the expression of each gene category classified by gene length (**a**, **c**, **e**, **g**, **i**) or gene age grade (**b**, **d**, **f**, **h**, **j**) of the five species. Developmental stage-specificity (SS) of a given gene is simply represented by the number of stages in which the gene is expressed. The values of upper and lower quartile are indicated as upper and lower edges of the box, and the values of median are indicated as a red bar in the box. The differences of SS distribution between the neighboring classes are examined by Mann–Whitney *U* test. The corrected *P* values are shown in the top of each panel. The *P* values marked with red color are those less than 0.05. The abbreviations of the gene age grades are the same as those in Fig. 3

Complex genes tended to be expressed widely across the stages of embryonic development (Fig. 4a, c, g, i, for GL, and Additional file 1: Fig. S13 for the other 3 complexity factors), whereas simple genes tended to be expressed in specific stages. These results indicated that complex genes contributed little to the diversity of proteomes during different stages of development. However, much more obvious differences between young and old genes could be observed. The young genes tended to be expressed in specific stages (Fig. 4 b, d, f, h, i, for GOT_Ens, Additional file 1: Fig. S14 for the other 3 age grade factors), indicating that the young genes contribute to the diversity of proteomes in different stages of development. Similar results were obtained from mouse adult organs, tissues and cell types (OTCs) (Additional file 1: Fig. S15), indicating young genes contribute to the diversity of different adult OTCs.

When gene complexity and gene age degree were combined, we found that old genes were widely expressed regardless of their complexity level (Additional file 1: Fig. S16). On the contrary, most of the young genes tend to be expressed SOTC (stage, organ, tissue and cell type)-specifically when compared to all of the other classes of genes (Additional file 1: Fig. S16). These results indicated that gene age degree has greater power than gene complexity to determine the SOTC-specificity, implying young genes contribute more to the diversity of different spatiotemporal states than simple genes.

For miRNA genes, it is also obvious that old miRNAs tend to express widely, whereas young miRNA tend to express at specific OTCs (Additional file 1: Fig. S17a, d). However, there are no obvious differences in SOTC-specificity among the miRNAs with different complexity degrees (Additional file 1: Fig. S17b, c, e, f). Thus, the results from miRNA confirmed that gene age degree is more powerful than gene complexity to determine the SOTC-specificity.

All these results indicated that young genes contribute to the diversity of proteomes in different stages of development and different adult OTCs.

### Gene complexity and novelty contribute to organismal complexity at two different levels

According to the preceding results, we inferred that complex and young genes contribute to the organismal complexity at two different levels. Complex genes are utilized preferentially in certain states (certain developmental stages or certain organs, tissues and cell types). Almost in each proteome, the complex genes are significantly over-represented, contributing to the complexity of each proteome. In contrast, although young genes are under-represented in each individual state, they tend to have a higher stage-specificity, contributing to the diversity between different proteomes, which in turn facilitates the complexity of the higher-level system (organism complexity in this study). Both the complexity of each individual proteome and the diversity of the proteomes at different states contribute to the formation of organism complexity. This is the so-called two-level model in this paper.

Next, we focused on the detailed contribution patterns of complex genes to the complexity of individual proteome in certain states, and how the young genes contribute to the diversity of proteomes in different spatiotemporal states.

**Fig. 5:**
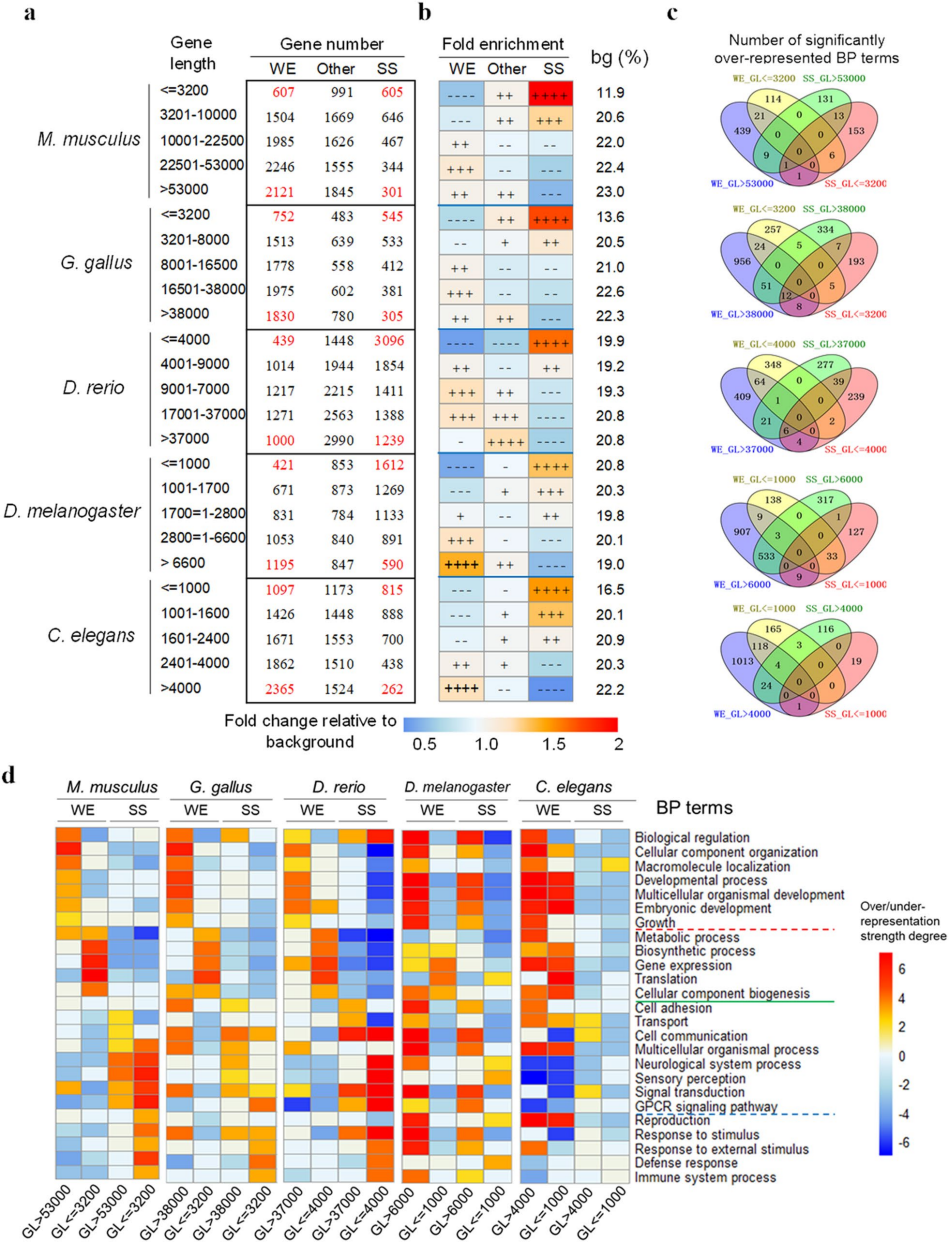
Detailed expression and functional characteristics of complex genes. **a** Shown are the detailed gene numbers of each category classified based on gene complexity grades (5 grades for gene length of the five species) and 3 grades of gene expression width (WE, widely expressed; SS, stage-specific and Other). The gene numbers of key categories are shown in red. **b** The percentages of protein-coding genes (PCGs) of each complexity grade in each age degree category were calculated and divided by the expected percentage. Heat map showing the fold enrichment values obtained from this division. The expected percentage was the percentage of PCGs of each complexity grade in the genome of each species, represented as ‘bg (%)’ in the right region of the panel. The symbols representing over-/under-representation strength are the same as those in Fig. 2. **c** Venn diagrams of the significantly over-represented GO terms (biological processes, BPs) for the four categories of interest. **d** Functional characteristics of the four categories of interest. The extent of over- and under-representation is shown by 14 grades (− 7 to 7; see “Methods” for details). The significantly over-represented BP terms of the two main classes for mouse, WE and SS, are separated by the solid green line. Other dashed lines separate the significantly over-represented BP terms of sub-classes

According to the preceding results, the complex genes tend to be expressed widely across different developmental stages and adult OTCs. Thus, we inferred that the complex genes tend to facilitate the formation of the common complex structures and functions present across different spatial and temporal states. To describe these common complex structures and functions, we set up two controls: widely expressed (WE) simple genes and stage-specific (SS) complex genes. We classified all of the PCGs based on gene complexity grades (5 grades for gene length, CRMN and protein length; 4 grades for DNIR) and 3 grades of gene expression width (Fig. 5a). The gene distribution pattern across these categories was consistent with the results of the gene spatiotemporal specificity analyses (Fig. 4a-d); that is, complex genes tend to be widely expressed across different stages during the development of the five species. This result is confirmed by the over-representation analyses (Fig. 5b, Additional file 7 : Table S9).

For the biological function analysis, we focused on four categories: complex widely expressed (WE) genes, simple WE genes, complex stage-specific (SS) genes and simple SS genes. The characteristics of the gene functions of these four categories were explored based on GO annotation and the hypergeometric distribution model, using all of the genes with BP (biological process) term annotations as the background. The significantly over-represented terms of biological processes (BPs) were counted and compared across these four categories. The two WE categories were obviously closer because they shared more over-represented BP terms, whereas there were almost no shared terms between the WE and SS categories (Fig. 5c). In detail, the complex and simple widely expressed genes obviously participate in different biological processes (Fig. 5d). The complex widely expressed genes tend to take part in such biological processes as biological regulation, catabolic processes, cellular component organization, transcription, cellular localization, cell cycle and cellular component biogenesis. The complex widely expressed genes contribute to biological complexity in each individual state during these biological processes. On the contrary, the simple widely expressed genes tend to take part in translation, transport and the generation of precursor metabolites and energy. These results provided the functional characteristics of complex and simple widely expressed genes, marking for the first time that widely expressed genes were classified according to their complexity level and their functional characteristics were explored. Until now, only the functional characteristics of widely expressed genes had been known. Here, the specific functional features of complex widely expressed genes were deciphered and compared with those of simple widely expressed genes. Furthermore, the complex/simple stage-specific genes clearly have different functional characteristics compared with the complex/simple WE genes. Notably, the BP term over-/under-presentation patterns are more similar among the three higher species, and there is obvious difference between the higher species and the two lower species in this study. For example, in *D. melanogaster*, most of the BP terms in Fig. 5c are significantly over-represented in the long genes, regardless of their stage-specificity. This suggests that the long genes defined in this study (the top 20% in each species) may have different function distributions.

For the analysis of mouse, the gene expression data in the adult OTCs are also taken into the calculation of expression width. The results about the distribution of widely expressed and stage- or OTC-specific genes and their function characteristics (Additional file 1: Fig. S18) were similar to the analysis based on only developmental stage-specificity data. The significantly over-represented gene knockout phenotype terms further confirmed the preceding results (Supplementary results in Additional file 1 and Table S10 in Additional file 8). When exploring the functional characteristics of complex widely expressed genes using the pathway view, some interesting clues were found. There are 14 significantly over-represented pathways in complex widely expressed genes (Additional file 9: Table S11). Interestingly, the high-complexity widely expressed genes tended to be distributed in the middle nodes of the signaling pathways, and they tended to participate in multiple pathways (Additional file 1: Fig. S18 d1–d9).

**Fig. 6:**
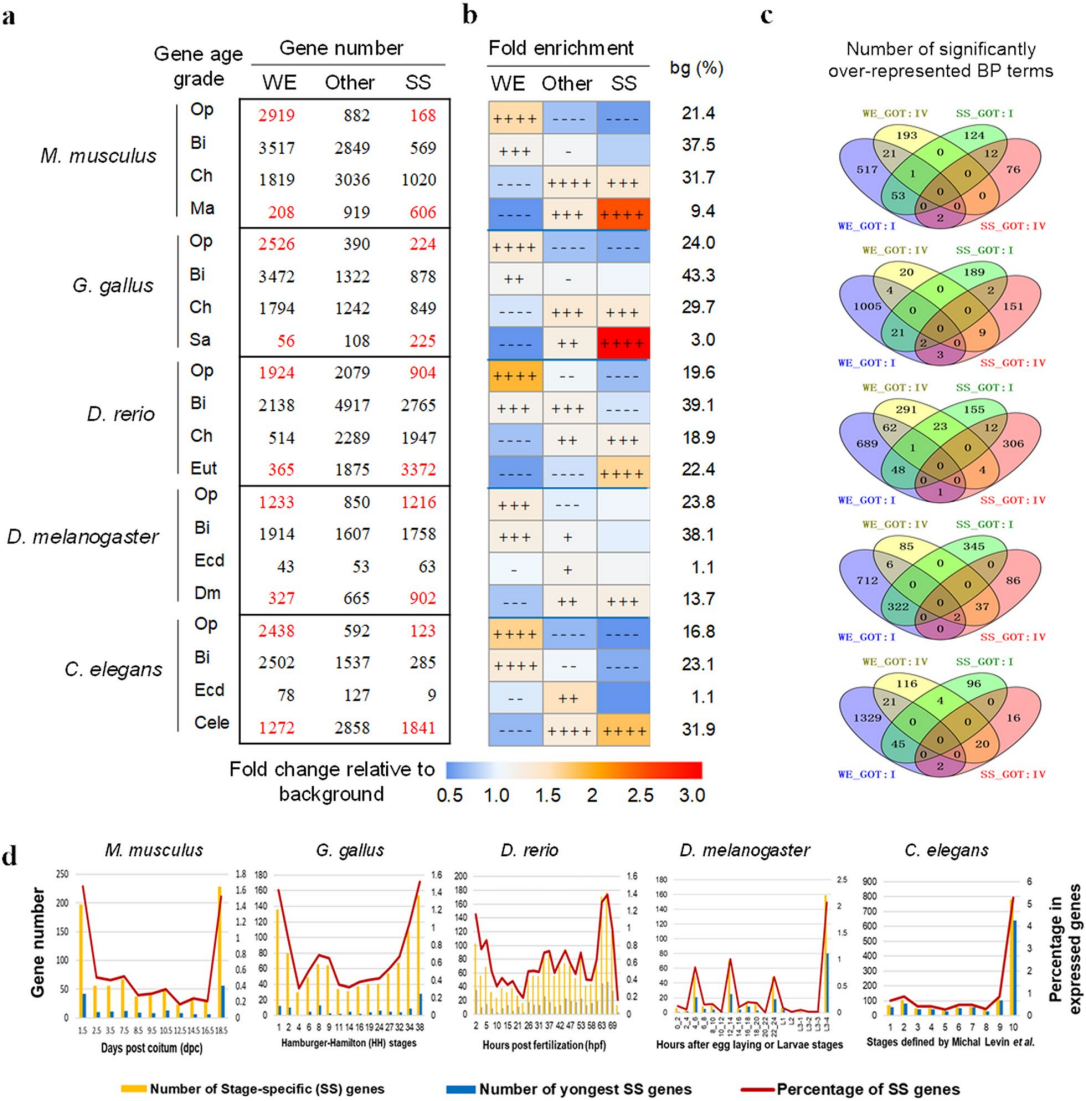
Detailed expression and functional characteristics of young genes. **a** Shown are the detailed gene numbers of each category classified based on gene age grades (4 grades of GOT_Ens of the five species) and 3 grades of gene expression width (WE, widely expressed; SS, stage-specific and Other). The gene numbers of key categories are shown in red. **b** The meaning of heat map is similar to that of Fig. 5b. The symbols representing over-/under-representation strength are the same as those in Fig. 2. **c** Venn diagrams of the significantly over-represented GO terms (biological processes, BPs) for the four categories of interest. **d** The distribution of stage-specific (SS) genes and stage-specific young genes among the different developmental stages analyzed. The numbers of SS genes and the numbers of youngest SS genes are shown as histograms, referencing the left axis in each sub-panel. The percentages of SS genes in the expressed genes in each stage are shown as line charts, referencing the right axis in each sub-panel

To explore the functional characteristics of stage-specific young genes compared with widely expressed young genes and stage-specific old genes, once again all of the PCGs were classified based on gene age grades and 3 grades of gene expression width (Fig. 6a). The gene numbers in these categories were consistent with the result of gene spatiotemporal specificity analyses (Fig. 4e–h); that is, young genes tend to be expressed specifically during the development. This result is confirmed by the over-representation analyses (Fig. 6b, Additional file 7 : Table S9). The GO annotation focused on these four categories: old widely expressed genes, young widely expressed genes, old stage-specific genes and young stage-specific genes. The two ‘old’ categories (GOT: I) were obviously closer because they shared common over-represented BP terms (Fig. 6c). The similar results were obtained based on the analysis of SOTC-specificity of mouse (Additional file 1: Fig. S19). Specifically, the biological functions of the SOTC-specific novel genes were mainly related to signal transduction, immune system processes, sensory perception and multicellular organism processes (Additional file 1: Fig. S19c). Interestingly, novel OTC-specific genes were not expressed evenly among different OTCs; instead, they tended to be concentrated in the testes and OTCs of the nervous system (Additional file 1: Fig. S19d, e). This result indicated that the young genes tend to contribute to the specificity of these special OTCs.

Another interesting phenomenon is about the expression distribution of the stage-specific genes during embryonic and larval development (Fig. 6d). There are much more stage-specific genes expressed during the very early and late stages in *M. musculus*, *G. gallus* and *D. rerio*. However, this trend is not so obvious in *D. melanogaster* and *C. elegans*. Instead, in the development of these two species, there is only one much higher peak of the stage-specific gene number in the larval stage. This indicates that the difference between early- and mid-stage embryonic developments in Protostomia is not so much obvious as Deuterostomia.

## Discussion

### A ‘two-level’ model: new insights into genome complexity realization

Since the accomplishment of genome sequencing for several model organisms, the relationship between the complexity of genome and organism has become a focus of genome studies [41, 42]. However, as more contributing factors to genome complexity have been presented, the picture has grown increasingly complicated and confused. Ours is the first attempt to categorize all gene properties into two groups (Fig. 1), i.e., the complexity and age degree of genes/gene products. This classification system offers us a generalized and clear framework that can incorporate most genome complexity factors. More importantly, based on this general classification, we can identify general trends in how the factors that contribute to genome complexity are utilized under certain conditions to form phenotypic complexity at the molecular and organism levels.

One of the core conclusions of this study is that complex genes are significantly over-represented in each stage of embryonic development (Fig. 3) and each of the adult OTCs (Additional file 1: Fig. S11), indicating that the complex genes tend to be utilized preferentially in each spatiotemporal state. On the contrary, young genes are usually significantly under-represented in each state (Fig. 3 and Additional file 1: Fig. S11) and tend to be expressed at specific states (Fig. 4, Additional file 1: Fig. S15–17). From this result, we can infer that complex gene/gene products contribute to the complexity of individual proteomes in certain states, whereas young gene/gene products contribute to the diversity of proteomes in different spatiotemporal states. Organism complexity is determined at two levels: the diversity of the spatiotemporal states constituting the organism and the complexity of each spatiotemporal state. This study reveals the respective contribution of complex and young genes to these two levels.

### New insights into the relationship between evolution and development from the viewpoint of genome complexity realization

The fundamental issue in evolutionary developmental (evo–devo) biology is how to formulate the relationships between evolutionary and developmental processes [10, 15, 19, 21]. Our study takes a new look at this old question. Genome complexity is the result of a long history of evolution. On the other hand, it is through the developmental process that genome complexity is represented as organismal phenotypic complexity. The main aim of this study is to explore how the genomic complexity-contributing factors, the ‘results’ of evolution, are utilized during development to form the organismal complexity at phenotypic level. This work may provide new insights into the relationship between evolution and development from the viewpoint of genome complexity realization.

First of all, complex genes tend to be utilized preferentially at the late stages of embryonic development, contributing to the increasing complexity of the embryo during development (Fig. 3). More specifically, this general trend occurs mainly due to the complex genes of medium age (Additional file 1: Fig. S8). Meanwhile, our results give new insights into the theory of evolution–development connection [10, 15, 40]. Our findings support the funnel-like model through a new observation of an obvious correlation between the time point of the change in over- and under-representation and the order of gene age (Fig. 3b, d, f, h, j, Additional file 1: Fig. S7). More importantly, we found some explanations about why there are different evo–devo relation models, for example, the seemingly contradictory ‘hourglass’ model and ‘funnel-like’ model. This ‘paradox’ may be due to three aspects of causes. First, the ways used for the comparison may affect the results. ‘Conservation’ has two meanings: One is that the expression pattern is conserved among different species. Another one is referring to the evolutionary age of the expressed genes in a certain developmental stage. In the two previous studies that obtained the funnel-like model [15, 16], their conclusions were based on the analyses of the trend of gene age in the genes expressed during each stage of development. They found that the age of genes expressed in early stage tend to be old and gene duplication and birth were the most rare compared with other stages (see Additional file 1: Table S12 for the detailed description). They did not compare the gene expression pattern between different species. Second, the method used to utilize the gene expression data may also be a cause leading to the different models. Support for the funnel-like model is based mostly on qualitative data (only considering if a given gene is expressed or not, Additional file 1: Table S12) [15, 16], whereas support for the hourglass model is based mostly on quantitative data (considering the amount of gene expression, Additional file 1: Table S12) [15, 18–21]. Barbara Piasecka et al. can obtain different models using the same expression dataset if they calculated using quantitative and qualitative manner, respectively. Our work indicated that at the qualitative view, the old genes tend to be over-represented with the strongest strength at the early stages of embryonic development, supporting the early conservation model (funnel-like model for animal development). Third, different samples (the scope for the comparison analysis) will lead to different conclusions about evo–devo relationship. For example, based on the comparison of gene expression pattern between *C. elegans* and *A. nanus*, a recent study [43] suggested a more complicated, funnel-like pattern of developmental constraints than previously recognized. They found that the level of conservation is throughout morphogenesis stage and the divergence level does not increase. This may be due to the similar morphology between these two species. Altogether, the ways for the comparison, the methods for gene expression calculation and the species scope of the comparison may all affect the evo–devo relationship model observed in a given analysis. The different evo–devo relationship models are not really contradictory. In the further analysis about this question, we will try to decipher the biological significance behind the different models obtained with different ways.

## Conclusion

This study, for the first time, introduces a ‘two-level’ model of the realization of genome complexity at phenotypic level: Complex genes contribute to the complexity of individual proteomes in certain states, whereas young genes contribute to the diversity of proteomes in different spatiotemporal states. This study also gets new insights into the evo–devo relationship: An obvious correlation between the time point of the change in over- and under-representation and the order of gene age was observed, which supports the funnel-like model from a new viewpoint. We also found the probable causes for the different ‘evo–devo relation’ models.

## Methods

### Datasets of gene expression during development

The gene expression datasets were downloaded from the GEO [44] or ArrayExpress [45] databases (see Additional file 1: Table S1 for details and corresponding references). To validate the core conclusion of this study under various conditions, the gene expression datasets selected were as complete as possible, representing the states of all the stages and spaces. For microarray data, the presence or absence of one gene in a certain OTC or condition is calculated using the presence/absence calls by MAS 5.0 algorithm (MAS5) [46]. The RNA-seq fastq files were subjected to quality control using FastQC v0.11.5 (http://www.bioinformatics.babraham.ac.uk/projects/fastqc/). The adapters and low-quality areas were removed with FASTX-Toolkit v0.013 (http://hannonlab.cshl.edu/fastx_toolkit/). The reads were mapped to the *Drosophila melanogaster* genome build BDGP6 using HISAT2 v2.0.4 [47]. Expression levels were calculated as read counts using HTSeq v0.6.1 [48], and the genes with more than 10 reads detected were regarded as expressed genes.

### Determination of developmental stages

For *Mus musculus*, the embryonic developmental process was divided into 5 stages, including Cleavage, Blastula, Neurula, Organogenesis, and Fetus, according to the definitions and descriptions for each stage from Bgee database (http://bgee.unil.ch/bgee/bgee?page=expression&action=easy_search). What is more, the ‘phylotypic stage’ is determined by its morphological characteristics described in Ref. [19] and the information from Bgee database.

For *Gallus gallus*, the method of Hamburger–Hamilton [49] was used to measure the stages of embryonic development. Here, for the data we used, we divided the embryonic process into 4 stages, including Primitive streak, Neurulation, early Organogenesis (for the common features among vertebrates) and late Organogenesis (for the Avian-specific features) [10].

For *Danio rerio*, for the data we used [50], we grouped the 106 samples into 28 groups and divided the developmental process into 6 stages: Cleavage, Blastula, Gastrula, Segmentation, Pharyngula and Hatching. The ‘phylotypic stage’ is about from 11.5 h to 43 h post-fertilization according to Ref. [21].

For *D. melanogaster*, we divided the early developmental process into 6 stages, including Cleavage, Blastoderm + Gastrulation, Germ band elongation and retraction, Head involution, Differentiation, and Larvae [21]. For the data we used here [51], the ‘phylotypic stage’ is about from 6.5 h to 9.5 h according to Ref. [21].

For *Caenorhabditis elegans*, for the data we used, seven stages including 4-cell stage, E-cell division (E-div), division of the AB lineage (ABdiv, 4 time points), Ventral Enclosure (VE), Comma Stage (CS), Movement (Mov) and First stage larva (L1) were assigned. The ‘phylotypic stage’ is about during the VE stage according to Ref. [52].

### Calculation and classification of parameters of gene complexity

Four factors contributing to gene complexity, including gene length (GL), *cis*-regulatory module number (CRMN), protein length (PL) and domain number including repeats (DNIR) in one protein, were selected to represent the complexity grade of one gene. GL was calculated by the start and end site information of the gene in Ensembl release 75 (http://www.ensembl.org/) extracted by BioMart [53]. CRMN was calculated based on the *cis*-regulatory module information of each gene in PReMod database [30] for *Mus musculus*, the position weight matrices (PWMs) in CIS-BP database and mapping DNA sequence [54] for *Gallus gallus* and *Danio rerio*, i-cisTarget [55] for *D. melanogaster* and PhyloNet_sites [56] for *C. elegans*. PL was calculated directly by the number of amino acids in the protein sequence from the FASTA file stored in Ensembl FTP site (ftp://ftp.ensembl.org/pub/current_fasta). The methods for domain identification and DNIR calculation are the same as our previous study [5].

### Calculation and classification of parameters of gene novelty

The gene origin time used in this study was defined by two methods: One is from a consensus gene age dataset which integrated 13 orthology inference algorithms [33]. This kind of gene origin time was named as GOT_Mode. Another method was defined by the most recent common ancestor (MRCA) of the species containing the gene based on the orthology relationships extracted from the EnsemblCompara database [34]. As described by Moyers and Zhang, there are biases in the gene age annotation inferred from BLAST-like alignments, especially in de novo gene identification and gene age determination for short proteins [57, 58]. The TreeBeST pipeline used to construct EnsemblCompara employed a synteny metric that provides a measure of gene order conservation [34]. With this approach, the potential bias in de novo gene identification introduced by BLAST-like alignments can be well controlled [59]. To avoid the gene age determination bias for short proteins, we selected the short proteins (< 100 a.a) to run BLASTp against all of the protein sequences from the species included in Ensembl database. When determining the homology relationship, we did not judge only by *E*-value, but also considered the matched percentage and identity values. This way, the short old proteins could be assigned correct ages. For example, Sarcolipin (ENSMUSP00000036950 encoded by ENSMUSG00000042045) is a very short protein (31 amino acids). According to the homology relationship annotation in EnsemblCompara (V75), Sarcolipin of mouse only has one ortholog in *Rattus norvegicus*. However, with our modified method, we can find the orthologs of Sarcolipin in *Macropus eugenii*, *Sus scrofa*, *Homo sapiens*, *Oryctolagus cuniculus* and *Rattus norvegicus*. Thus, the age of Sarcolipin should be defined as Theria-specific. In total, 611 protein-coding genes of mouse were assigned with a modified age. The gene age order (the oldest genes have the smallest order value, see Additional file 1: Table S13 for the detailed information) was used to partly represent each gene’s novelty and was named GOT_Ens. To simplify the classification, all the PCGs in each species were divided into four grades (Additional file 1: Table S7). In this study, the young genes are the (super)phylum-specific genes for each species. For example, for *M. musculus*, *G. gallus* and *D. rerio*, Chordata-specific genes are regarded as the young genes. For *D. melanogaster* and *C. elegans*, Ecdysozoa-specific genes are regarded as the young genes.

We also introduced last duplication time (LDT) as a novelty factor. Most of the duplicated genes evolved new or sub-functions after duplication [60–62]. Some genes originated very early, but were duplicated recently. These genes have novel properties, but if we used the GOT alone to measure novelty, they would be classified as old genes. Thus, it was necessary to take the last duplication time into account when measuring novelty. The LDT of one gene was determined by the paralogy annotation in the Ensembl database retrieved by BioMart [63]. The values assigned to LDT were also based on the evolutionary time order (Additional file 1: Table S13). We assumed that the duplication events took place after its origination. Furthermore, we assumed that singletons were the remnants of the two duplicates, one of which was lost during evolution. Thus, the LDT of the singleton was assigned with GOT minus 0.5. According to LDT, all of the PCGs can be partitioned into four or five classes (Additional file 1: Table S7).

The domain age was assigned according to its phylogenetic distribution using the taxonomy information in the Pfam database [64, 65] (http://ftp://ftp.ebi.ac.uk/pub/databases/Pfam/). As in our previous study [5], the domain age characteristics of a protein are represented by the youngest domain within the protein, named as the DOT (domain origin time) of the protein. To simplify the classification, all of the mouse PCGs were divided into five or six groups according to the ancestors of domain origination (Additional file 1: Table S7).

### The complexity and age degree for microRNA genes

The complexity of miRNA gene is represented by the number of its target genes. Target gene information is obtained based on two databases, respectively: miRTarBase [66] and PITA (PITA score < − 10) [67]. The age degree of miRNA gene is inferred from the miRNA family database (miFam.dat) in miRbase. The origin time of miRNA genes was defined by the most recent common ancestor (MRCA) of the species containing the gene based on miFam.

### Statistical analysis

All of the correlations were defined on the nonparametric Spearman rank correlation, which assesses how well the relationship between two variables can be described using a monotonic function. Spearman rank correlation was performed using MATLAB 7.11.0.

The difference tests of the stage-specificity of the genes in different categories in our analysis were performed using the Wilcoxon rank-sum test, a nonparametric statistical hypothesis test for assessing whether two independent samples of observations have equally large values. We performed Wilcoxon rank-sum test using MATLAB 7.11.0, in which the *P* values will be adjusted using ‘normal_approximation’ method for the large samples (n > 10).

The over- or under-representation analysis is based on hypergeometric distribution model, and the *P* values were corrected using the Benjamini–Hochberg method. The over- or under- representation strengths are represented by − log(*P*) or log(*P*), respectively. When the heat maps were used to represent the over-/under-representation strengths, the values of ∓log(*P*) were transformed into 14 grades (− 7 to 7): − 7, log(*P*) ≤ − 30; − 6, − 30 < log(*P*) ≤ − 15; − 5, − 15 < log(*P*) ≤ − 10; − 4, − 10 < log(*P*) ≤ − 5; − 3, − 5<log(*P*) ≤ − 2; − 2, − 2<log(*P*) ≤ − 1.301; − 0.25, − 1.301 < log(*P*) ≤ 0; 0.25, 0 < −log(*P*) < 1.301; 2, 1.301 ≤ −log(*P*) < 2; 3, 2 ≤ −log(*P*) < 5; 4, 5 ≤ −log(*P*) < 10; 5, 10 ≤ −log(*P*) < 15; 6, 15 ≤ −log(*P*) < 30; 7, −log(*P*) ≥ 30.

The Venn diagrams were drawn by a tool named ‘Venny’ (http://bioinfogp.cnb.csic.es/tools/venny/index.html).

## Additional files

**Additional file 1.** The supplemental descriptions of part of the results and the supplemental methods, tables and figures.

**Additional file 2: Table S2.** The values of the gene complexity and age degree of all PCGs.

**Additional file 3: Table S4. ** Short genes anticipating signal transduction in mouse.

**Additional file 4: Table S5. ** The over- or under-representation analysis of the relationship between gene complexity and phenotype count.

**Additional file 5: Table S6.** The significantly over/under-represented KEGG pathways in the complex or simple genes.

**Additional file 6: Table S8. ** Raw data of the distribution of complex genes across different age categories in the five species (related to figure 2).

**Additional file 7: Table S9. ** The raw data for the over/under-representation analysis of the widely expressed or stage-specific genes among different categories (related to Figure 5b and Figure 6b).

**Additional file 8: Table S10. ** The significantly over-represented Mammalian Phenotype terms in widely expressed/SOTC-specific complex/simple genes.

**Additional file 9: Table S11. ** The significantly over/under-represented KEGG pathways in the widely expressed/SOTC-specific complex/simple genes.

## Abbreviations

CRMN: *cis*-regulatory module number
DNIR: domain number including repeats in a protein
DOT: protein domain origin time
GL: gene length
GOT: gene origin time
LDT: last duplication time
OTC: tissues and cell type
PCG: protein-coding gene
PL: protein length
SOTC-S: stage, organ, tissue, cell type-specific
SS: stage-specific
WE: widely expressed

## References

[1] Hahn MW, Wray GA (2002). The g-value paradox. Evol Dev.

[2] Schad E, Tompa P, Hegyi H (2011). The relationship between proteome size, structural disorder and organism complexity. Genome Biol.

[3] Szathmary E, Jordan F, Pal C (2001). Molecular biology and evolution. Can genes explain biological complexity?. Science.

[4] Taft RJ, Pheasant M, Mattick JS (2007). The relationship between non-protein-coding DNA and eukaryotic complexity. BioEssays.

[5] Yang D (2012). General trends in the utilization of structural factors contributing to biological complexity. Mol Biol Evol.

[6] He X, Zhang J (2005). Gene complexity and gene duplicability. Curr Biol.

[7] Lynch M, Conery JS (2003). The origins of genome complexity. Science.

[8] Alhindi T (2017). Protein interaction evolution from promiscuity to specificity with reduced flexibility in an increasingly complex network. Sci Rep.

[9] Kryukov K (2012). A new database (GCD) on genome composition for eukaryote and prokaryote genome sequences and their initial analyses. Genome Biol Evol.

[10] Abzhanov A (2013). von Baer’s law for the ages: lost and found principles of developmental evolution. Trends Genet.

[11] Okasha S (2010). Does diversity always grow?. Nature.

[12] Oakley TH, Rivera AS (2008). Genomics and the evolutionary origins of nervous system complexity. Curr Opin Genet Dev.

[13] Carroll SB (2001). Chance and necessity: the evolution of morphological complexity and diversity. Nature.

[14] von Baer KE. Uber Entwickelungsgeschichte der Thiere: Beobachtung und Reflektion. 1828.

[15] Piasecka B (2013). The hourglass and the early conservation models—co-existing patterns of developmental constraints in vertebrates. PLoS Genet.

[16] Roux J, Robinson-Rechavi M (2008). Developmental constraints on vertebrate genome evolution. PLoS Genet.

[17] Duboule D (1994). Temporal colinearity and the phylotypic progression: a basis for the stability of a vertebrate Bauplan and the evolution of morphologies through heterochrony. Development.

[18] Quint M (2012). A transcriptomic hourglass in plant embryogenesis. Nature.

[19] Irie N, Kuratani S (2011). Comparative transcriptome analysis reveals vertebrate phylotypic period during organogenesis. Nat Commun.

[20] Kalinka AT (2010). Gene expression divergence recapitulates the developmental hourglass model. Nature.

[21] Domazet-Loso T, Tautz D (2010). A phylogenetically based transcriptome age index mirrors ontogenetic divergence patterns. Nature.

[22] Capra JA (2013). How old is my gene?. Trends Genet.

[23] Kaessmann H (2010). Origins, evolution, and phenotypic impact of new genes. Genome Res.

[24] Kawashima T (2009). Domain shuffling and the evolution of vertebrates. Genome Res.

[25] Lespinet O (2002). The role of lineage-specific gene family expansion in the evolution of eukaryotes. Genome Res.

[26] Bailey JA (2002). Recent segmental duplications in the human genome. Science.

[27] Zhang W (2015). New genes drive the evolution of gene interaction networks in the human and mouse genomes. Genome Biol.

[28] Chen S, Krinsky BH, Long M (2013). New genes as drivers of phenotypic evolution. Nat Rev Genet.

[29] Lu ZX, Huang Q, Su B (2009). Functional characterization of the human-specific (type II) form of kallikrein 8, a gene involved in learning and memory. Cell Res.

[30] Ferretti V (2007). PReMod: a database of genome-wide mammalian cis-regulatory module predictions. Nucleic Acids Res.

[31] Vogel C, Chothia C (2006). Protein family expansions and biological complexity. PLoS Comput Biol.

[32] Xu A (2015). Evolutionary characteristics of missing proteins: insights into the evolution of human chromosomes related to missing-protein-encoding genes. J Proteome Res.

[33] Liebeskind BJ, McWhite CD, Marcotte EM (2016). Towards consensus gene ages. Genome Biol Evol.

[34] Vilella AJ (2009). EnsemblCompara GeneTrees: complete, duplication-aware phylogenetic trees in vertebrates. Genome Res.

[35] Capra JA, Williams AG, Pollard KS (2012). ProteinHistorian: tools for the comparative analysis of eukaryote protein origin. PLoS Comput Biol.

[36] Prat Y (2009). Codon usage is associated with the evolutionary age of genes in metazoan genomes. BMC Evol Biol.

[37] Mattick JS (2001). Non-coding RNAs: the architects of eukaryotic complexity. EMBO Rep.

[38] Guo H (2010). Mammalian microRNAs predominantly act to decrease target mRNA levels. Nature.

[39] Catalanotto C, Cogoni C, Zardo G (2016). MicroRNA in control of gene expression: an overview of nuclear functions. Int J Mol Sci.

[40] Kalinka AT, Tomancak P (2012). The evolution of early animal embryos: conservation or divergence?. Trends Ecol Evol.

[41] Vinogradov AE, Anatskaya OV (2007). Organismal complexity, cell differentiation and gene expression: human over mouse. Nucleic Acids Res.

[42] Prochnik SE (2010). Genomic analysis of organismal complexity in the multicellular green alga Volvox carteri. Science.

[43] Schiffer PH (2018). The gene regulatory program of *Acrobeloides nanus* reveals conservation of phylum-specific expression. Proc Natl Acad Sci USA.

[44] Barrett T (2009). NCBI GEO: archive for high-throughput functional genomic data. Nucleic Acids Res.

[45] Rustici G (2013). ArrayExpress update—trends in database growth and links to data analysis tools. Nucleic Acids Res.

[46] Hubbell E, Liu WM, Mei R (2002). Robust estimators for expression analysis. Bioinformatics.

[47] Kim D, Langmead B, Salzberg SL (2015). HISAT: a fast spliced aligner with low memory requirements. Nat Methods.

[48] Anders S, Pyl PT, Huber W (2015). HTSeq—a python framework to work with high-throughput sequencing data. Bioinformatics.

[49] Hamburger V, Hamilton HL (1951). A series of normal stages in the development of the chick embryo. J Morphol.

[50] Levin M (2016). The mid-developmental transition and the evolution of animal body plans. Nature.

[51] Graveley BR (2011). The developmental transcriptome of *Drosophila melanogaster*. Nature.

[52] Levin M (2012). Developmental milestones punctuate gene expression in the *Caenorhabditis embryo*. Dev Cell.

[53] Kasprzyk A (2011). BioMart: driving a paradigm change in biological data management. Database.

[54] Weirauch MT (2014). Determination and inference of eukaryotic transcription factor sequence specificity. Cell.

[55] Imrichova H (2015). i-cisTarget 2015 update: generalized cis-regulatory enrichment analysis in human, mouse and fly. Nucleic Acids Res.

[56] Zhao G (2012). Conserved motifs and prediction of regulatory modules in *Caenorhabditis elegans*. G3.

[57] Moyers BA, Zhang J (2015). Phylostratigraphic bias creates spurious patterns of genome evolution. Mol Biol Evol.

[58] Moyers BA, Zhang J (2016). Evaluating phylostratigraphic evidence for widespread de novo gene birth in genome evolution. Mol Biol Evol.

[59] Chen JY (2015). Emergence, retention and selection: a trilogy of origination for functional de novo proteins from ancestral LncRNAs in primates. PLoS Genet.

[60] Assis R, Bachtrog D (2013). Neofunctionalization of young duplicate genes in Drosophila. Proc Natl Acad Sci USA.

[61] Qian W, Zhang J (2014). Genomic evidence for adaptation by gene duplication. Genome Res.

[62] Han MV (2009). Adaptive evolution of young gene duplicates in mammals. Genome Res.

[63] Kinsella RJ (2011). Ensembl BioMarts: a hub for data retrieval across taxonomic space. Database.

[64] Punta M (2012). The Pfam protein families database. Nucleic Acids Res.

[65] Winstanley HF, Abeln S, Deane CM (2005). How old is your fold?. Bioinformatics.

[66] Chou CH (2016). miRTarBase 2016: updates to the experimentally validated miRNA-target interactions database. Nucleic Acids Res.

[67] Kertesz M (2007). The role of site accessibility in microRNA target recognition. Nat Genet.

