# Supplementary material for: A two-level model for the role of complex and young genes in the formation of organism complexity and new insights into the relationship between evolution and development
Source: EvoDevo. 2018 Nov 12;9:22. doi: 10.1186/s13227-018-0111-4 (PMC6231269; doi:10.1186/s13227-018-0111-4)
Supplement: Supplementary file 1 — Additional file 1. The supplemental descriptions of part of the results and the supplemental methods, tables and figures. [file 13227_2018_111_MOESM1_ESM.pdf]

A two-level model for the role of complex and young genes in the formation of organism complexity and new insights into the relationship between evolution and development

(Supporting information)

Contents

Supplemental description of part of the results

- Functional comparison among the four types of complex genes and the three types of young genes
- Some interesting results related to the general utilization pattern of each gene category classified according to DOT at certain temporal-spatial states
- The general utilization patterns of the gene complexity/novelty factors at certain adult OTCs
- The significantly over-represented knockout phenotype (Mammalian Phenotype) terms in widely expressed/SOTC-specific complex/simple genes

Supplemental methods

- Determining the complexity of organisms or tissues
- Gene knockout phenotype annotation data

Supplemental tables

Table S1. Overview of the expression data sets used in the analyses.

Table S2. The values of the gene complexity and age degree of all PCGs (in the file 'Additional file 2-Table S2.xlsx').

Table S3. The detailed information of the cell type number and gene complexity factors used in the figure S1.

Table S4. Short genes anticipating signal transduction in mouse (in the file 'Additional file 3-Table S4.xlsx').

Table S5. The over- or under-representation analysis of the relationship between gene

complexity and phenotype count (in the file ‘Additional file 4-Table S5.xlsx’).

Table S6. The significantly over/under-represented KEGG pathways in the complex or simple genes (in the file ‘Additional file 5-Table S6.xlsx’).

Table S7. The detailed information for the names and abbreviations of the gene age grades.

Table S8. Raw data of the distribution of complex genes across different age categories in the five species (related to figure 2) (in the file ‘Additional file 6-Table S8.xls’).

Table S9. The raw data for the over/under-representation analysis of the widely expressed or stage-specific genes among different categories (related to Figure 5b and Figure 6b) (in the file ‘Additional file 7-Table S9.xls’).

Table S10. The significantly over-represented Mammalian Phenotype terms in widely expressed/SOTC-specific complex/simple genes (in the file ‘Additional file 8-Table S10.xls’).

Table S11. The significantly over/under-represented KEGG pathways in the widely expressed/SOTC-specific complex/simple genes. (in the file ‘Additional file 9-Table S11.xlsx’).

Table S12. The methods and conclusions of studies supporting ‘funnel-like’ model or ‘hourglass’ model.

Table S13. Orders of evolutionary time.

Supplemental figures

Figure S1. The correlation between the mean value of gene complexity factors in one genome and the organism complexity, and the functional characteristics of complex genes compared with simple genes.

Figure S2. Comparison among the four types of complex genes and the three types of young genes.

Figure S3. Schematic diagram for the potential reasons why genes originating from the common ancestor of Bilateria have the largest proportion of highly complex genes.

Figure S4. The relationships between miRNA gene age and its target gene count.

Figure S5. Functional characteristics of the complex genes with different age degrees.

Figure S6. Over- or under-representation strengths of each gene category classified by the 3 gene complexity factors during the development of 5 species.

Figure S7. Over- or under-representation strengths of each gene category classified by

the 3 gene age grade factors during the development of 5 species.

Figure S8. Over- or under-representation strengths of each gene category classified by the combination of 4 gene complexity factors and 4 degrees of gene age during embryonic development.

Figure S9. Over- or under-representation strengths of each gene category classified by 4 gene complexity factors during mouse organs development.

Figure S10. Over- or under-representation strengths of each gene category classified by 3 gene age factors during mouse organs development.

Figure S11. Over- or under-representation strengths of each gene category classified by gene complexity/age degree factors in the mouse adult organs, tissues and cell types (OTCs).

Figure S12. Over- or under-representation strengths of each miRNA category classified by gene complexity/age degree factors in the mouse organs, tissues and cell types (OTCs).

Figure S13. Stage-specificity of the expression of each gene category classified by 3 gene complexity factors.

Figure S14. Stage -specificity of the expression of each gene category classified by 3 gene age degree factors.

Figure S15. OTC-specificity of the expression of each gene category classified by gene complexity/age degree factors.

Figure S16. Developmental stage-specificity or OTC-specificity of the expression of each gene category classified by the combination of 4 gene complexity factors and 4 degrees of gene age during embryonic development.

Figure S17. OTC-specificity of the expression of each miRNA category classified by gene complexity/age degree factors.

Figure S18. Supplemental results of the expression and functional characteristics of complex genes.

Figure S19. Supplemental results of the expression and functional characteristics of young genes.

Supplemental description of part of the results

Functional comparison among the four types of complex genes and the three types of young genes

The GO over/under-representation analysis revealed that the complex genes specific to each of the four complexity factors (GL, CRMN, PL and DNIR) have distinct specific functional features (Fig. S2c). The gene-length-specific complex genes (GL>53000, CRMN<=10, PL<=720, DNIR<=2) tend to anticipate the metabolic processes of lipid, carbohydrate and alcohol, and the regulation of transport. The CRMN-specific complex genes (CRMN>10, GL<=53000, PL<=720, DNIR<=2) tend to anticipate reproduction and the regulation of cell proliferation, death, apoptosis. The PL-specific complex genes (PL>720, GL<=53000, CRMN<=10, DNIR<=2) tend to be involved in cell cycle and protein localization and transport. The DNIR-specific complex genes (DNIR>2, GL<=53000, CRMN<=10, PL<=720) tend to take part in mRNA metabolic process, transmembrane transport, and the responses to stress and external stimulus. Further specific investigation of these special functional features will give new insights into the knowledge of these special complex genes and the related biological processes. The three types of young genes also have distinct specific functional features (Fig. S2f)

Some interesting results related to the general utilization pattern of each gene category classified according to DOT at certain temporal-spatial states

In the view of DOT, proteins only containing old domains ("cellular organisms" grade and Eukaryota grade) are over-represented in all stages of embryonic development. Interestingly, the over-representation strength of Eukaryota grade genes is higher than that of "cellular organisms" grade. Proteins containing young domains (Chordata and Mammalia grades) or domains with middle age (Metazoa grade) are under-represented in all stages of embryonic development (Fig. 3h). These general trends are also observed during certain organ development (Fig. S9i, j, k, l), certain

adult OTCs (Fig. S11h), and are also valid based on the similar analyses of other 4 species (Fig. S13b, d, f, h).

An interesting phenomenon that the representation-strength exchanges along with the birth was found in the liver development data (Fig. S10j). In the embryonic stages of liver, the over-representation strength of Eukaryota grade genes is higher than that of "cellular organisms" grade, which is similar to the whole embryo. But after birth, the over-representation strengths of the two class are exchanged, that is, genes only containing oldest domains are mostly over-represented in mouse liver after birth, which may be related to the functional transition of fetal liver and adult liver. As we have known, the main physiological function of adult liver is metabolism which is the oldest type of function originating from cellular organisms.

The general utilization patterns of the gene complexity/novelty factors at certain adult OTCs

The main conclusions about the general utilization patterns of the gene complexity/novelty factors at certain adult OTCs are the same to the results obtained based on the analyses of embryonic development data. The complex genes are significantly over-represented in each OTC, especially in the OTCs of the neural system (Fig. S11a, b, c, d). The young genes are significantly under-represented in each OTC (Fig. S11e, f, g, h). These results are consistent with our previous study[1] in which we only focused on the protein domain related parameters.

The significantly over-represented knockout phenotype (Mammalian Phenotype) terms in widely expressed/SOTC-specific complex/simple genes

The gene knockout phenotypes can represent the biological functions of the gene. To further confirm the functional characteristics of the four categories (WE_complex, WE_simple, SOTC-S_ complex, and SOTC-S_ simple,) observed in the analyses based on GO annotation (Fig. 5c), the significantly over-represented gene knockout phenotype terms for these four categories were calculated (Additional file 8: Table S10). The widely expressed complex genes tend to have the general, essential and complex

knockout phenotypes, such as mortality/aging, growth/size/body region phenotype, behavior/neurological phenotype, homeostasis/metabolism phenotype, nervous system phenotype, *etc*. On the contrary, the SOTC-specific complex genes tend to have the knockout phenotypes specific to a given system or organ, such as the phenotypes of behavior, skeleton, cardiovascular system, digestive/alimentary system, immune system, limbs/digits/tail, reproductive system, respiratory system, vision/eye, *etc*. These results confirmed the main conclusion of figure s18c in the view of knockout phenotype.

Supplemental methods

Determining the complexity of organisms or tissues

Organism complexity was defined by the numbers of different cell types constituting it, which are obtained from the reference[2]. Notably, the measurement of organism complexity is not simply equal to the measure of cell types. For example, the connection between cells and the complexity of each type of cells also contribute to the organismal complexity. But currently, there are no a more complicated measurement to obtain the complexity of tens of organisms than cell type number.

Gene knockout phenotype annotation data

The knockout phenotype annotation for mouse protein-coding genes (PCGs) was retrieved from the web site of International Mouse Phenotyping Consortium (IMPC, ftp://ftp.ebi.ac.uk/pub/databases/impc/, updated at August 15, 2016). There are 2364 PCGs of mouse with knockout phenotype annotation in this database. The Mammalian Phenotype (MP) terms were used to calculate the knockout phenotype count which can approximately present the functional complexity of the gene. MP terms were also used to the over/under-representation analysis to investigate the significantly over-represented knockout phenotype terms in widely expressed/SOTC-specific complex/simple genes. The phenotype annotation data of other species were obtained from these databases: ZFIN (http://zfin.org/) for *Danio rerio*, Flybase (http://flybase.org/) for *Drosophila melanogaster* and Wormbase (https://wormbase.org/) for *Caenorhabditis elegans*.

Supplemental tables

Table S1 Overview of the expression data sets used in the analyses

Species	Datasets (ID)	Journal	Description
M. musculus	GSE7309	Development[3]	embryos at day 1.5 (cont. 1.5, 2-cell stage), day 2.5 (cont. 2.5, 4- to 8-cell), and day 3.5 (cont. 3.5, morula to blastocyst)
	GSE28389	Nature Communications[4]	whole embryos at 8 different stages (Stages: E7.5, E8.5, E9.5, E10.5, E12.5, E14.5, E16.5, E18.5)
	GSE13149	Genomics[5]	14 time points across the C57/B6 mouse liver development
	GSE8091	Proteomics[6]	3 embryonic stages (E9.5, E11.5, E13.5) of mouse brain development
	GSE35366	*PLoS Genet*[7]	3 developmental stages (E14, P0, P14) of mouse brain development
	GSE1479	----	C57BL/6 Benchmark Set for Early Cardiac Development
	GSE20954	*PLoS One*[8]	mRNA expression profile in mouse lung development
	GSE10246	Immunome Res[9]	91 OTCs (organs, tissues, or cell types) were taken from naïve male C57BL6 mice. In this study, we select 68 normal OTCs excluding the cell lines or cells treated with LPS or chemicals.
	-----	Cell[10]	A mammalian microRNA expression atlas based on small RNA library sequencing (Miltenyi Biotec miRNA microarray). The miRNA expression data of 68 mouse OTCs under various conditions was provided.
	GSE40499	Genome Res[11]	5 adult tissues (brain, cerebellum, heart, kidney and testis), and 3 samples from spermatogenic cells in adult mouse testis (Sertoli cells, spermatocytes and spermatids)

G. gallus	E-MTAB-366	Nature Communications[4]	G.gallus whole embryos at 15 different stages (Stages:HH1,2,4,6,8,9,11,14,16,19,24,27,32,34,38)
D. rerio	GSE60619	Nature[12]	The 106 samples (from 40 to 4240 minutes after fertilization) were grouped into 28 groups and divided the developmental process into 6 stages: Cleavage, Blastula, Gastrula, Segmentation, Pharyngula and Hatching
D. melanogaster	SRA009364	Nature[13]	The data of 12 timepoints of embryonic development stages and 6 samples of larvae stages were selected for this analysis..
C. elegans	GSE31422-GPL14144	Developmental Cell[14]	10 time points of the development of *C. elegans* embryos from the 4-cell stage to the first stage larva.

Table S3. The detailed information of the cell type number and gene complexity factors used in the figure S1.

Clade	Species	Complexity (Cell type number)	Mean gene length (bp)
Deuterostomia	*Homo sapiens*	169	60197.84
	Pan troglodytes	169	51788.90
	Bos taurus	159	46857.26
	Canis familiaris	159	49473.94
	Felis silvestris cattus (Felis silvestris catus)	159	46177.84
	Mus musculus	159	41148.64
	Rattus norvegicus	159	36255.84
	Tupaia belangeri	159	55549.86
	Gallus gallus	154	28823.35
	Anolis carolinensis	140	42969.42
	Xenopus tropicalis	129.5	29446.92
	Danio rerio	119.5	28041.94
	Takifugu rubripes	119.5	8577.41
	Tetraodon nigroviridis	119.5	879.31
	Branchiostoma floridae	100	9389.18
	Ciona intestinalis	74	5941.78
Protostomia	*Drosophila melanogaster*	64	6538.54
	Anopheles gambiae	64	5872.80
	Caenorhabditis elegans	28.5	2817.45
Sponges and cnidarians	*Hydra magnipapillata*	22	15044.30
	Nematostella vectensis	22	4608.72
	Amphimedon queenslandica	16	4033.62
Protozoa	*Leishmania major*	7.85	1716.01
	Trypanosoma brucei	7.85	1347.31
	Plasmodium falciparum	7.85	2464.39
	Theileria annulata	7.85	1778.83
	Dictyostelium discoideum	4.65	1716.15
Plant	*Populus balsamifera*	28.5	853.73
	Arabidopsis thaliana	27.25	2228.17
	Brachypodium distachyon	27.25	3757.22
	Sorghum bicolor	27.25	2794.09
	Vitis vinifera	27.25	6697.62
	Oryza sativa	27.25	3948.42
	Zea mays	27.25	4585.65

	Selaginella moellendorfii	25	1705.13
	Physcomitrella patens	22	3934.76
	Chlamydomonas reinhardtii	12.5	4375.18
	Ostreococcus tauri	12.5	1347.88
	Ostreococcus lucimarinus	12.5	1301.69
Fungi	*Neurospora crassa*	5.55	2263.15
	Schizosaccharomyces pombe	4.35	1846.17
	Ustilago maydis	4.35	1801.08
	Encephalitozoon cuniculi	3.35	1068.07
	Saccharomyces cerevisiae	3.05	1369.19
	Kluyveromyces lactis	3.05	1406.08
	Yarrowia lipolytica	3.05	1381.72

Clade	Species	Complexity (Cell type number)	Mean protein length (a.a)	Mean proportion of multi-domain proteins
Deuterostomia	*Homo sapiens*	169	553.43	0.18
	Pan troglodytes	169	535.89	0.17
	Bos taurus	159	535.66	0.17
	Canis familiaris	159	547.08	0.18
	Felis silvestris cattus (Felis silvestris catus)	159	544.72	0.12
	Mus musculus	159	520.19	0.18
	Rattus norvegicus	159	481.43	0.15
	Tupaia belangeri	159	526.81	0.11
	Gallus gallus	154	483.93	0.16
	Anolis carolinensis	140	509.36	0.16
	Xenopus tropicalis	129.5	543.00	0.19
	Danio rerio	119.5	533.16	0.18
	Takifugu rubripes	119.5	563.94	0.20
	Tetraodon nigroviridis	119.5	504.91	0.17
	Branchiostoma floridae	100	463.17	0.13
	Ciona intestinalis	74	321.95	0.07

Protostomia	*Drosophila melanogaster*	64	534.08	0.11
	Caenorhabditis elegans	28.5	410.35	0.06
Sponges and cnidarians	*Hydra magnipapillata*	22	423.27	0.08
	Nematostella vectensis	22	334.73	0.07
	Amphimedon queenslandica	16	533.87	0.14
Protozoa	*Theileria annulata*	7.85	534.13	0.05
Plant	*Arabidopsis thaliana*	27.25	405.35	0.10
	Brachypodium distachyon	27.25	441.60	0.10
	Sorghum bicolor	27.25	383.49	0.07
	Vitis vinifera	27.25	462.68	0.12
	Zea mays	27.25	413.46	0.03
	Selaginella moellendorfii	25	393.41	0.09
	Physcomitrella patens	22	508.58	0.10
	Chlamydomonas reinhardtii	12.5	453.94	0.05
	Ostreococcus tauri	12.5	424.52	0.07
Fungi	*Schizosaccharomyces pombe*	4.35	464.23	0.10
	Saccharomyces cerevisiae	3.05	449.82	0.08
	Kluyveromyces lactis	3.05	484.29	0.09

Table S7. The detailed information for the names and abbreviations of the gene age grades

	Grade	*M. musculus*	*G. gallus*	*D. rerio*	*D. melanogaster*	*C. elegans*
GOT_Ens	I	Opisthokonta (Op)	Opisthokonta (Op)	Opisthokonta (Op)	Opisthokonta (Op)	Opisthokonta (Op)
	II	Bilateria(Bi)	Bilateria(Bi)	Bilateria(Bi)	Bilateria(Bi)	Bilateria(Bi)
	III	Chordata (Ch)	Chordata (Ch)	Chordata (Ch)	Ecdysozoa (Ecd)	Ecdysozoa (Ecd)
	IV	Mammalia (Ma)	Sauropsida (Sa)	Euteleostomi (Eut)	*D. melanogaster* (Dm)	*C. elegans* (Cele)
GOT_Mode	I	cellular organisms (Ce)	cellular organisms (Ce)	cellular organisms (Ce)	cellular organisms (Ce)	cellular organisms (Ce)
	II	Eukaryota (Eu)	Eukaryota (Eu)	Eukaryota (Eu)	Eukaryota (Eu)	Eukaryota (Eu)
	III	Eumetazoa (EuMe)	Eumetazoa (EuMe)	Eumetazoa (EuMe)	Eumetazoa (EuMe)	Eumetazoa (EuMe)
	IV	Vertebrata (Ve)	Vertebrata (Ve)	Vertebrata (Ve)	Arthropoda (Ar)	Ecdysozoa (Ecd)
	V	Mammalia (Ma)	*G. Gallus* (Ga)	Teleostomi (Tel)	*D. melanogaster* (Dm)	*C. elegans* (Cele)
LDT	I	Opisthokonta (Op)	Opisthokonta (Op)	Opisthokonta (Op)	Opisthokonta (Op)	Opisthokonta (Op)
	II	Chordata (Ch)	Chordata (Ch)	Bilateria(Bi)	Bilateria(Bi)	Bilateria(Bi)
	III	Euteleostomi (Eut)	Euteleostomi (Eut)	Euteleostomi (Eut)	Ecdysozoa (Ecd)	Ecdysozoa (Ecd)
	IV	Mammalia (Ma)	*G. Gallus* (Ga)	Otophysi (Ot)	*D. melanogaster* (Dm)	*C. elegans* (Cele)
	V	Mus (Mus)				
DOT	I	cellular organisms (Ce)	cellular organisms (Ce)	cellular organisms (Ce)	cellular organisms (Ce)	cellular organisms (Ce)
	II	Eukaryota (Eu)	Eukaryota (Eu)	Eukaryota (Eu)	Eukaryota (Eu)	Eukaryota (Eu)
	III	Metazoa (Me)	Metazoa (Me)	Metazoa (Me)	Metazoa (Me)	Metazoa (Me)
	IV	Chordata (Ch)	Chordata (Ch)	Chordata (Ch)	Insecta (In)	Nematoda (Ne)
	V	Mammalia (Ma)	NoD	NoD	NoD	NoD
	VI	NoD				

Table S12. The methods and conclusions of studies supporting ‘funnel-like’ model or ‘hourglass’ model.

Model	Studies Journal. (Author. Year)	Analysis Methods	Data type	Conclusions
funnel-like model (early-stage conserved model)	This study	Over- or under-representation analysis	qualitative	There is an obvious correlation between the timepoint-changing of the over-/under-representation strength and the order of gene age.
	PLoS genetics[15]	Correlation analysis (between gene proportion and developmental timing)	qualitative	Genes expressed early in development (1) have a more dramatic effect of knock-out or mutation and (2) are more likely to revert to single copy after whole genome duplication, relative to genes expressed late. (*Figure 1, 6 in the original paper*)
	PloS Genetics[16]	Measures of transcriptome age (not considering expression level); Over- or under-representation analysis	qualitative	(1) The age of genes expressed in early stage tend to be old.<br>(2) Gene duplication and birth were the most rare for genes expressed in early development
Hourglass model (mid-stage conserved model)	Nature[17]	Measures of transcriptome age (considering expression level)	quantitative	The phylotypic stage express the oldest transcriptome set and that younger sets are expressed during early and late development.
	Nature[18]	Expression level divergence among 6 Drosophila species	quantitative	Expression is maximally conserved during the phylotypic period.
	Nat Commun.[4]	Correlation analysis to evaluate transcriptome similarity	quantitative	The transcriptome at pharyngula stage is most conserved among several model vertebrate embryos.

	Nature[19]	Measures of transcriptome age (considering expression level)	quantitative	Provide phylotranscriptomic evidence for a molecular embryonic hourglass in Arabidopsis thaliana.
	PloS Genetics[16]	examining the presence of highly conserved non-coding elements (HCNEs)	quantitative	Sequences of regulatory regions being most conserved for genes expressed in mid-development

Table S13. Orders of evolutionary time. The evolutionary time was represented by the ancestors in the phylogeny tree. Each ancestor was given a matched order according to the divergent time in the phylogeny tree based on the Taxonomy database in NCBI. To represent the gene novelty, the oldest ancestor has the smallest order value. The red records are involved in the GOT, LDT or DOT determination in this study.

For the genes of *Mus musculus*		
**Ancestors**	**Order**	**Taxonomy_id**
Cellular organisms	1	131567
Eukaryota	2	2759
Opisthokonta	3	33154
Metazoa	4	33208
Eumetazoa	5	6072
Bilateria	6	33213
Deuterostomia	7	33511
Chordata	8	7711
Craniata	9	115366
Vertebrata	10	1261581
Gnathostomata	11	35060
Teleostomi	12	117570
Euteleostomi	13	117571
Sarcopterygii	14	8287
Tetrapoda	15	32523
Amniota	16	32524
Mammalia	17	40674
Theria	18	32525
Eutheria	19	9347
Boreoeutheria	20	1437010
Euarchontoglires	21	314146
Glires	22	314147
Rodentia	23	9989
Sciurognathi	24	33553
Muroidea	25	337687
Muridae	26	10066
Murinae	27	39107
Mus	28	10088
Mus musculus	29	10090
**For the genes of *Gallus gallus***		
**Ancestors**	**Order**	**Taxonomy_id**
cellular organisms	1	131567
Eukaryota	2	2759

Opisthokonta	3	33154
Metazoa	4	33208
Eumetazoa	5	6072
Bilateria	6	33213
Deuterostomia	7	33511
Chordata	8	7711
Craniata	9	115366
Vertebrata	10	7742
Gnathostomata	11	7776
Teleostomi	12	117570
Euteleostomi	13	117571
Sarcopterygii	14	8287
Dipnotetrapodomorpha	15	1338369
Tetrapoda	16	32523
Amniota	17	32524
Sauropsida	18	8457
Sauria	19	32561
Archelosauria	20	1329799
Archosauria	21	8492
Dinosauria	22	436486
Saurischia	23	436489
Theropoda	24	436491
Coelurosauria	25	436492
Aves	26	8782
Neognathae	27	8825
Galloanserae	28	1549675
Galliformes	29	8976
Phasianidae	30	9005
Phasianinae	31	9072
Gallus	32	9030
Gallus gallus	33	9031

For the genes of *Danio rerio*

**Ancestors**	**Order**	**Taxonomy_id**
cellular organisms	1	131567
Eukaryota	2	2759
Opisthokonta	3	33154
Metazoa	4	33208
Eumetazoa	5	6072
Bilateria	6	33213
Deuterostomia	7	33511
Chordata	8	7711
Craniata	9	115366

Vertebrata	10	7742
Gnathostomata	11	7776
Teleostomi	12	117570
Euteleostomi	13	117571
Actinopterygii	14	7898
Actinopteri	15	186623
Neopterygii	16	41665
Teleostei	17	32443
Osteoglossocephalai	18	1489341
Clupeocephala	19	186625
Otomorpha	20	186634
Ostariophysi	21	32519
Otophysi	22	186626
Cypriniphysae	23	186627
Cypriniformes	24	7952
Cyprinoidea	25	30727
Cyprinidae	26	7953
Danio	27	7954
Danio rerio	28	7955

For the genes of *Drosophila melanogaster*

**Ancestors**	**Order**	**Taxonomy_id**
cellular organisms	1	131567
Eukaryota	2	2759
Opisthokonta	3	33154
Metazoa	4	33208
Eumetazoa	5	6072
Bilateria	6	33213
Protostomia	7	33317
Ecdysozoa	8	1206794
Panarthropoda	9	88770
Arthropoda	10	6656
Mandibulata	11	197563
Pancrustacea	12	197562
Hexapoda	13	6960
Insecta	14	50557
Dicondylia	15	85512
Pterygota	16	300552
Neoptera	17	33340
Holometabola	18	33392
Diptera	19	7147
Brachycera	20	7203
Muscomorpha	21	43733

Eremoneura	22	480118
Cyclorrhapha	23	480117
Schizophora	24	43738
Acalyptratae	25	43741
Ephydroidea	26	43746
Drosophilidae	27	7214
Drosophilinae	28	43845
Drosophilini	29	46877
Drosophila	30	7215
Sophophora	31	32341
Drosophila melanogaster	32	7227

For the genes of *Caenorhabditis elegans*

Ancestors	Order	Taxonomy_id
cellular organisms	1	131567
Eukaryota	2	2759
Opisthokonta	3	33154
Metazoa	4	33208
Eumetazoa	5	6072
Bilateria	6	33213
Protostomia	7	33317
Ecdysozoa	8	1206794
Nematoda	9	6231
Chromadorea	10	119089
Rhabditida	11	6236
Rhabditoidea	12	55879
Rhabditidae	13	6243
Peloderinae	14	55885
Caenorhabditis	15	6237
Caenorhabditis elegans	16	6239

Supplemental figure legends

Figure S1. The correlation between the mean value of gene complexity factors in one genome and the organism complexity, and the functional characteristics of complex genes compared with simple genes. Organism complexity is measured by the number of cell types constituting it. (**a**), correlation between the mean GL (gene length) and the organism complexity. (**b**), correlation between the mean PL (protein length) and the organism complexity. (**c**), correlation between the proportion of multi-domain proteins in the genome and the organism complexity. (**d**) Over-/under-representation analysis of the biological processes for the genes with different complexity degrees (all the PCGs as the background). The over-/under-representation strengths are shown by 14 grades (-7~7, see Methods for the detailed information). (**e1-e4**) Over-/under-representation analysis of the knockout phenotype terms (Mammalian Phenotype terms, abbreviated as "MP") for the genes with different complexity degrees. All the PCGs with MP annotation as the background. (**f1-f4**) Numbers of KEGG pathways in which the genes with different complexity degrees are involved are shown by cumulative probability. The differences between the neighboring classes and between the two classes with the largest difference are examined by Mann–Whitney U rank sum test, and the P values are shown in each panel.

Figure S2. Comparison among the four types of complex genes and the three types of young genes. (**a**), Venn diagrams of the four classes of complex genes. GL, gene length; CRMN, cis-regulatory module number; PL, protein length; DNIR, domain number including the repeats in a same protein. (**b**), Shown are the percentages of each categories classified by the number of classes the gene belongs to in each class of complex genes. For example, '4' represents that the gene belongs to 4 classes of complex genes, whereas '1' represents that the gene only belongs to 1 class of complex genes. (**c**), Over-/under-representation analysis of the biological processes for the genes belonging to 4 classes of complex genes (shown as 'Common complex' in the figure), class-specific complex genes (e. g. the complex genes only belonging to 1 class of complex genes), and the genes which are not complex (shown as 'Not complex' in the figure). The over-/under-representation strengths are shown by 14 grades (-7~7, see Methods for the detailed information). (**d**), Venn diagrams of the three classes of young genes. IV grade for the gene origin time (GOT) means the genes are mammalian-specific. V grade for the last duplication time (LDT) means the genes experienced duplication after the common ancestor of mammalian. V grade for the domain origin time (DOT) means the genes encoding at least one mammalian-specific protein domain. NoD means there isn't known conserved domains in the protein sequence. (**e**), Shown are the percentages of each categories classified by the number of classes the gene belongs to in each class of young genes. For example, '3' represents that the gene belongs to 3 classes of young genes, whereas '1' represents

that the gene only belongs to 1 class of young genes. (**f**), Over-/under-representation analysis of the biological processes for the genes belonging to 3 classes of young genes (shown as 'Common young' in the figure), class-specific young genes (e. g. the young genes only belonging to 1 class of young genes), and the genes which are not young (shown as 'Not young' in the figure). The methods for the calculation of over-/under-representation strengths are the same as panel c.

Figure S3. Schematic diagram for the potential reasons why genes originating from the common ancestor of Bilateria have the largest proportion of highly complex genes. The bars with different colors represent different genes. The different shapes embedded in each gene represent the different units of the gene. The length of the gene and the number of units in the gene can represent the gene complexity. The balance of two different trends are shown here. One trend is genes will become more and more complex during evolution. The other one is complex organisms, for example, the common ancestor of Bilateria, produced new complex genes.

Figure S4. The relationships between miRNA gene age and its target gene count. The relationships between miRNA gene complexity (measured by the target gene count) and age grade are shown as the proportions of the genes of each complexity grade in different age degree categories. The data of target gene count are based on miRTarBase (**a**) and PITA database (**b**). The percentage values are shown in the histograms. The abbreviations for the miRNA age grades: I, Metazoa-Chordata; II, Vertebrata-Amniota; III, Mammalia-Glires; IV, Rodentia-Murinae; V, Mus.

Figure S5. Functional characteristics of the complex genes with different age degrees. (**a**) Over-/under-representation analysis of the biological processes for the complex genes with different age degrees classified by the gene origin time (GOT). All the complex genes were regarded as the background when calculating. The methods for the calculation of over-/under-representation strengths are the same as Figure S3c. (**b1-b4**) Over-/under-representation analysis of the knockout phenotype terms (Mammalian Phenotype terms, abbreviated as "MP") for the complex genes with different age degrees. All the PCGs with MP annotation as the background. (**c1-c4**) Numbers of KEGG pathways in which the complex genes with different age degrees are involved are shown by cumulative probability. The differences between the neighboring classes are examined by Mann–Whitney U rank sum test, and the P values are shown in each panel. The abbreviations for the age grades: I, Opisthokonta-Eumetazoa; II, Bilateria-Deuterostomia; III, Chordata-Amniota; IV, Mammalia-Mus.

Figure S6. Over- or under-representation strengths of each gene category classified by the 3 gene complexity factors during the development of 5 species. Over- and under- representation are represented by -log(P) or log(P), respectively (see Methods for details). The red/blue dashed line represents the -/+log(P) value corresponding to significant over- or under-representation. The grey

shaded area represents the presumptive phylotypic phase. PCG refers to ‘protein-coding gene’. Developmental stages are separated by dashed light green lines and marked at the bottom of each panel. For *M. musculus*: Cleavage, Blastula (B), Neurula (N), Organogenesis, and Fetus (F). For *G. gallus*: Primitive streak (P.S.), Neurula, early Organogenesis (Organog.), and late Organogenesis (Organog.). For *D. rerio* Cleavage (C), Blastula (B), Gastrula (G), Segmentation (Segment.), Pharyngula (Pharyn.), and Hatching (Hat.). For *Drosophila melanogaster*: Cleavage (C), Blastoderm+Gastrulation (B.G.), Germ band elongation and retraction (Germ band.), Early of head involution (H), Differentiation, and Larvae. For *C. elegans*: 4-cell stage, E-cell division (E-div), division of the AB lineage (ABdiv), Ventral Enclosure (VE), Comma Stage (CS), Movement (Mov), and First stage larva (L1). The abbreviations for gene age grades: Op, Opisthokonta; Bi, Bilateria; Ch, Chordata; Ma, Mammalia; Eut, Euteleostomi; Ecd, Ecdysozoa; Dm, *D. melanogaster*; Cele, *C. elegans.*

Figure S7. Over- or under-representation strengths of each gene category classified by the 3 gene age grade factors during the development of 5 species. The analysis method and significance of red/blue dashed line are the same as that of figure s5.

Figure S8. Over- or under-representation strengths of each gene category classified by the combination of 4 gene complexity factors and 4 degrees of gene age during embryonic development. The over- or under- representation strengths are represented by -log(P) or log(P) respectively. The significances of red/blue dash line are the same with that of Figure 3. Developmental stages are separated by dashed light green lines and the grey shaded area same to figure 3. The abbreviations for the age grades: I, Opisthokonta-Eumetazoa; II, Bilateria-Deuterostomia; III, Chordata-Amniota; IV, Mammalia-Mus.

Figure S9. Over- or under-representation strengths of each gene category classified by 4 gene complexity factors during mouse organs development. Four organs were selected used in this analysis: brain (**a, e, i, m**), liver (**b, f, j, n**), heart (**c, g, k, o**) and lung (**d, h, l, p**). Gene length (**a-d**), cis-regulatory module number (CRMN, **e-h**), protein length (**i-l**), and domain number including repeats in one protein (DNIR, **m-p**). The analysis method and significance of red/blue dashed line are the same as that of figure 3. The vertical dash blue lines represent the time point of birth.

Figure S10. Over- or under-representation strengths of each gene category classified by 3 gene age factors during mouse organs development. The expression data of the four organs and the significance of red/blue dashed lines, the vertical dash blue lines are the same as that of Figure S7. The gene age factors include GOT (**a-d**), LDT (**e-h**), and DOT (**i-l**). The abbreviations: (**a-d**), Op-Eum, Opisthokonta-Eumetazoa; Bi-Deu, Bilateria-Deuterostomia; Ch-Am, Chordata-Amniota; Ma-Mus, Mammalia-Mus. (**e-h**), Op-Deu Opisthokonta- Deuterostomia; Ch-Tel, Chordata-Teleostomi;

Eut-Am, Euteleostomi-Amniota; Ma-Mur, Mammalia-Murinae. (**i-l**), Ce, Cellular organisms; Eu-Op, Eukaryota-Opisthokonta; Me-Deu, Metazoa-Deuterostomia; Ch-Am, Chordata-Amniota; Ma-Mus, Mammalia-Mus.

Figure S11. Over- or under-representation strengths of each gene category classified by gene complexity/age degree factors in the mouse adult organs, tissues and cell types (OTCs). The over- or under- representation strengths are represented by -log(P) or log(P) respectively (see methods for detail information). Red/blue dashed line represents the -/+log(P) value corresponding significant over- or underrepresentation. Tissues composed primarily of neurons are indicated with a purple bar. Leukomonocytes in peripheral blood or bone marrow are indicated with a green bar. The abbreviations used in the figure are the same as figure S6 and S7.

Figure S12. Stage-specificity of the expression of each gene category classified by 3 gene complexity factors. Developmental stage-specificity (SS) of a given gene is simply represented by the number of stages in which the gene expressed. The values of upper and lower quartile are indicated as upper and lower edges of the box, and the values of median are indicated as a red bar in the box. The differences of SS distribution between the neighboring classes are examined by Mann–Whitney U test. The corrected P values are shown in the top of each panel. The P values marked with red color are those less than 0.05. The abbreviations of the gene age grades are shown in table s7.

Figure S13. Stage-specificity of the expression of each gene category classified by 3 gene age grade factors. The statistical methods and the meanings of the symbols in the figure are the same as figure s12.

Figure S14. Over- or under-representation strengths of each miRNA category classified by gene age degree (a, d) or complexity (b, c, e, f) in the mouse organs, tissues and cell types (OTCs). The abbreviations for the miRNA age grades (a, d): I, Metazoa-Chordata; II, Vertebrata-Amniota; III, Mammalia-Glires; IV, Rodentia-Murinae; V, Mus. The data of target gene count are based on miRTarBase (**b, e**) and PITA database (**c, f**) respectively. The over- or under- representation strengths are represented by -log(P) or log(P) respectively (see methods for detail information).

Figure S15. OTC-specificity of the expression of each gene category classified by gene complexity/age degree factors. The statistical methods and the meanings of the symbols in the figure are the same as figure 4.

Figure S16. Developmental stage-specificity or OTC-specificity of the expression of each gene category classified by the combination of 4 gene complexity factors and 4 degrees of gene age during embryonic development. The 4 degrees of gene age (gene origin time) are the same as that

of figure S6. The statistical methods and the meanings of the symbols in the figure are the same as figure 4.

Figure S17. OTC-specificity of the expression of each miRNA category classified by gene age degree (a, d) or complexity factors (b, c, e, f). The data of miRNA age degree and target gene count are the same as that of figure s12. The statistical methods and the meanings of the symbols in the figure are the same as figure 4.

Figure S18. Supplemental results of the expression and functional characteristics of complex genes.

The detailed gene number of each category classified based on gene complexity grades (5 grades for gene length, CRMN and protein length; 4 grades for DNIR) and 3 grades of gene expression width are shown in panels **a**. The gene number of key categories are shown as red. Venn diagrams of the significantly over-represented GO terms (biological processes, BPs) for the four categories of interest are shown in panel **b1-b4**. **(c)**, functional characteristics of the four categories of interest. The extent of over- and under-representation is shown by 14 grades (-7~7; see Methods for details). The significantly over-represented BP terms of the two main classes, WE and SOTC-S, are separated by the solid green line. Other dashed lines separate the significantly over-represented BP terms of sub classes. (**d1-d9**), Shown are the gene expression and complexity characteristics in nine signaling pathways (the materials used here were permitted by Kanehisa *et al.*, who developed the KEGG database)[20]. Each node in the pathway view is colored by a box with six columns. The first column represents the expression feature of the gene (widely expressed or stage/OTC-specific). The 2nd-5th columns represent the complexity degrees of GL, CRMN, PL and DNIR respectively. The sixth column represents the number of KEGG pathways in which this gene is involved. The abbreviations used here: WE, widely expressed; SOTC-S, Stage, organ, tissue, cell type-specific. The age grades of GOT, LDT and DOT are the same as figure S3.

Figure S19. Supplemental results of the expression and functional characteristics of young genes.

The detailed gene number of each category classified based on gene age grades (4 grades for GOT; 5 grades for LDT and DOT) and 3 grades of gene expression width are shown in panels **a**. The gene number of key categories are shown as red. Venn diagrams of the significantly over-represented GO terms (biological processes, BPs) for the four categories of interest are shown in panel **b1-b4**. (**c**), Functional characteristics of the four interested categories classified by SOTC-specificity and gene age degree. The over-/under-representation strengths are shown by 14 grades (-7~7, see Methods for the detailed information). (**d**), the distribution of OTC-specific genes and OTC-specific young genes among the different OTCs analyzed. ‘1-4’ means the OTC-specific genes refer to the genes

expressed only in 1-4 OTCs. The top 15 OTCs are shown. (**e**), The distribution of the OTC-specific genes and OTC-specific young genes among different OTCs analyzed. "1" means the OTC-specific genes refer to the genes only express in one OTC. The top 15 OTCs are shown. The abbreviations used in this figure: WE, widely expressed; SOTC-S, Stage, organ, tissue, cell type-specific;

Supplemental References

1 Yang, D. *et al.* General trends in the utilization of structural factors contributing to biological complexity. *Molecular biology and evolution* **29**, 1957-1968, doi:10.1093/molbev/mss064 (2012).

2 Vogel, C. & Chothia, C. Protein family expansions and biological complexity. *PLoS Comput Biol* **2**, e48, doi:10.1371/journal.pcbi.0020048 (2006).

3 Maekawa, M., Yamamoto, T., Kohno, M., Takeichi, M. & Nishida, E. Requirement for ERK MAP kinase in mouse preimplantation development. *Development* **134**, 2751-2759, doi:10.1242/dev.003756 (2007).

4 Irie, N. & Kuratani, S. Comparative transcriptome analysis reveals vertebrate phylotypic period during organogenesis. *Nature communications* **2**, 248, doi:10.1038/ncomms1248 (2011).

5 Li, T. *et al.* Multi-stage analysis of gene expression and transcription regulation in C57/B6 mouse liver development. *Genomics* **93**, 235-242, doi:10.1016/j.ygeno.2008.10.006 (2009).

6 Hartl, D. *et al.* Transcriptome and proteome analysis of early embryonic mouse brain development. *Proteomics* **8**, 1257-1265, doi:10.1002/pmic.200700724 (2008).

7 Pramparo, T. *et al.* Global developmental gene expression and pathway analysis of normal brain development and mouse models of human neuronal migration defects. *PLoS genetics* **7**, e1001331, doi:10.1371/journal.pgen.1001331 (2011).

8 Dong, J. *et al.* MicroRNA networks in mouse lung organogenesis. *PloS one* **5**, e10854, doi:10.1371/journal.pone.0010854 (2010).

9 Lattin, J. E. *et al.* Expression analysis of G Protein-Coupled Receptors in mouse macrophages. *Immunome research* **4**, 5, doi:10.1186/1745-7580-4-5 (2008).

10 Landgraf, P. *et al.* A mammalian microRNA expression atlas based on small RNA library sequencing. *Cell* **129**, 1401-1414, doi:10.1016/j.cell.2007.04.040 (2007).

11 Meunier, J. *et al.* Birth and expression evolution of mammalian microRNA genes. *Genome research* **23**, 34-45, doi:10.1101/gr.140269.112 (2013).

12 Levin, M. *et al.* The mid-developmental transition and the evolution of animal body plans. *Nature* **531**, 637-641, doi:10.1038/nature16994 (2016).

13 Graveley, B. R. *et al.* The developmental transcriptome of Drosophila melanogaster. *Nature* **471**, 473-479, doi:10.1038/nature09715 (2011).

14 Levin, M., Hashimshony, T., Wagner, F. & Yanai, I. Developmental milestones punctuate gene expression in the Caenorhabditis embryo. *Dev Cell* **22**, 1101-1108, doi:10.1016/j.devcel.2012.04.004 (2012).

15 Roux, J. & Robinson-Rechavi, M. Developmental constraints on vertebrate genome

evolution. *PLoS genetics* **4**, e1000311, doi:10.1371/journal.pgen.1000311 (2008).
16 Piasecka, B., Lichocki, P., Moretti, S., Bergmann, S. & Robinson-Rechavi, M. The hourglass and the early conservation models--co-existing patterns of developmental constraints in vertebrates. *PLoS genetics* **9**, e1003476, doi:10.1371/journal.pgen.1003476 (2013).
17 Domazet-Loso, T. & Tautz, D. A phylogenetically based transcriptome age index mirrors ontogenetic divergence patterns. *Nature* **468**, 815-818, doi:10.1038/nature09632 (2010).
18 Kalinka, A. T. *et al.* Gene expression divergence recapitulates the developmental hourglass model. *Nature* **468**, 811-814, doi:10.1038/nature09634 (2010).
19 Quint, M. *et al.* A transcriptomic hourglass in plant embryogenesis. *Nature* **490**, 98-101, doi:10.1038/nature11394 (2012).
20 Kanehisa, M., Sato, Y., Kawashima, M., Furumichi, M. & Tanabe, M. KEGG as a reference resource for gene and protein annotation. *Nucleic Acids Res* **44**, D457-462, doi:10.1093/nar/gkv1070 (2016).

Figure S1

a

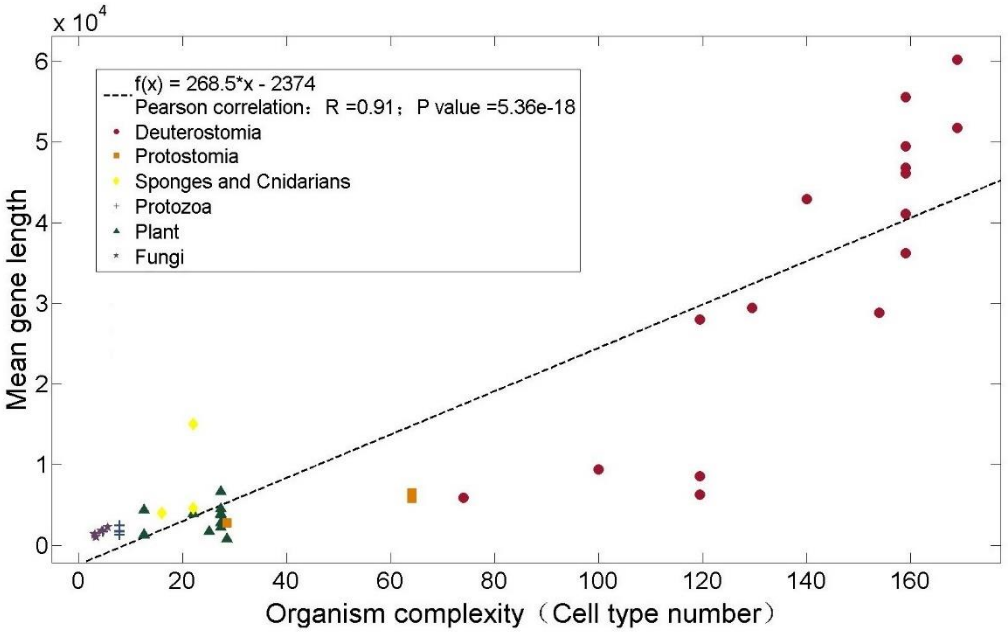
x 10^4
f(x) = 268.5*x - 2374
Pearson correlation：R =0.91；P value =5.36e-18
Deuterostomia
Protostomia
Sponges and Cnidarians
Protozoa
Plant
Fungi
Mean gene length
Organism complexity（Cell type number）
0
1
2
3
4
5
6
0
20
40
60
80
100
120
140
160

b

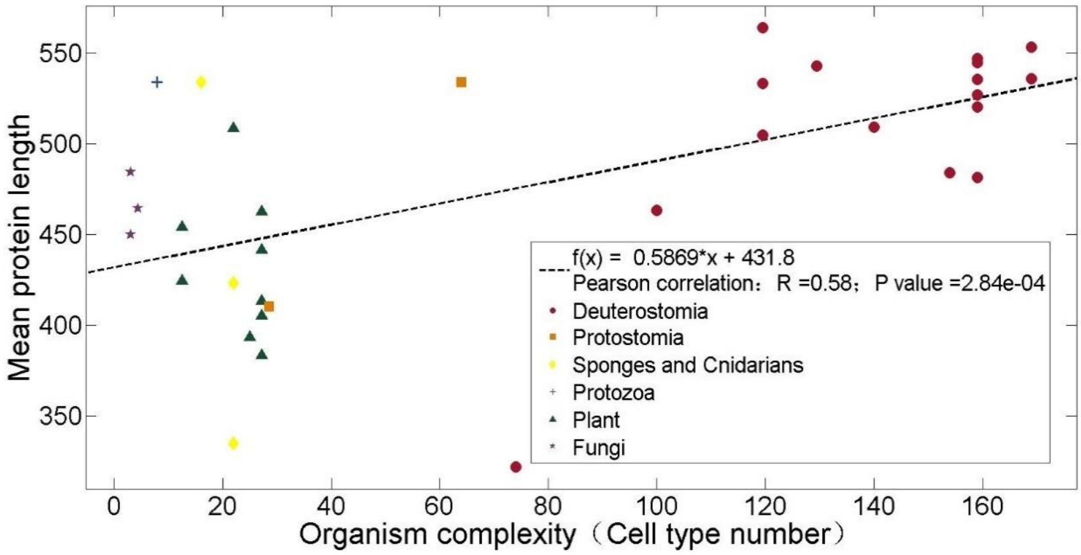
f(x) = 0.5869*x + 431.8
Pearson correlation：R =0.58；P value =2.84e-04
Deuterostomia
Protostomia
Sponges and Cnidarians
Protozoa
Plant
Fungi
Mean protein length
Organism complexity（Cell type number）
350
400
450
500
550
0
20
40
60
80
100
120
140
160

c

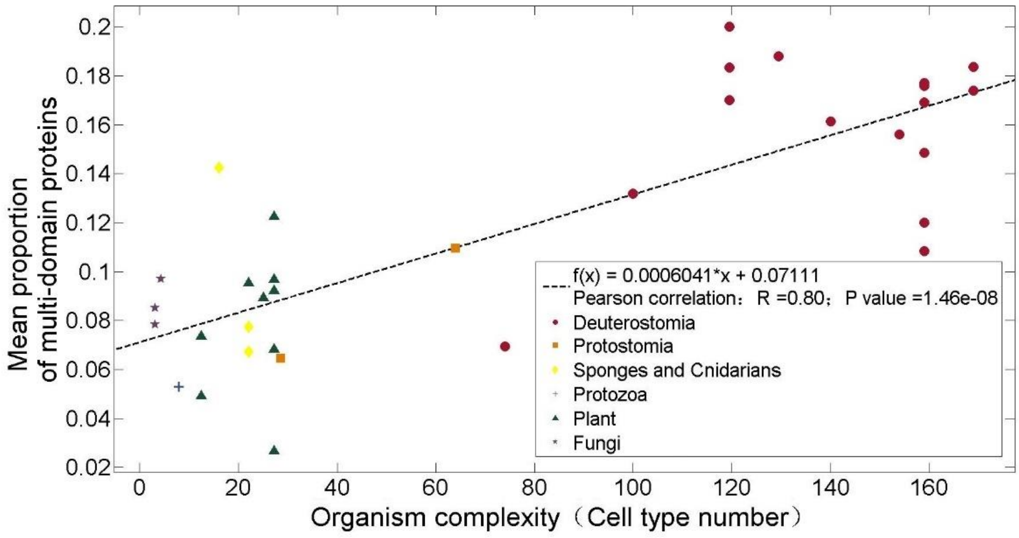

0.2
0.18
0.16
0.14
0.12
0.1
0.08
0.06
0.04
0.02
0
20
40
60
80
100
120
140
160
Mean proportion of multi-domain proteins
Organism complexity（Cell type number）
f(x) = 0.0006041*x + 0.07111
Pearson correlation：R =0.80；P value =1.46e-08
Deuterostomia
Protostomia
Sponges and Cnidarians
Protozoa
Plant
Fungi

d

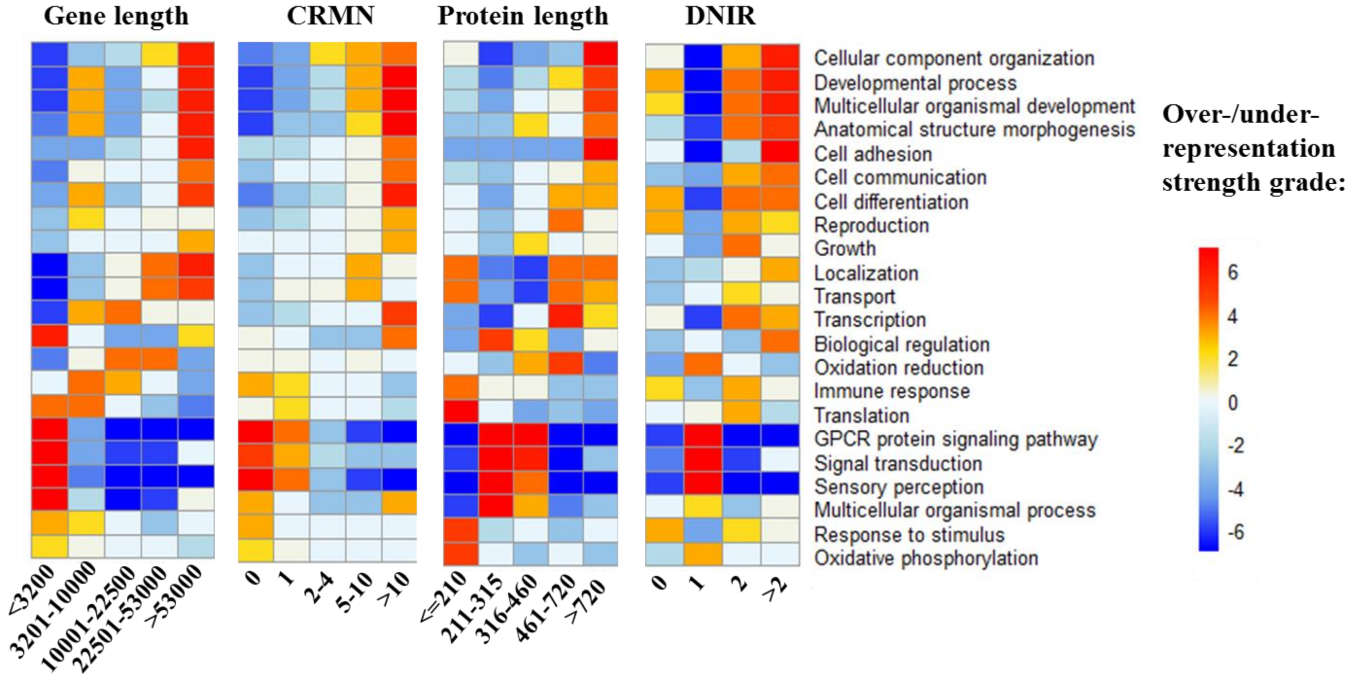

Gene length
CRMN
Protein length
DNIR
Cellular component organization
Developmental process
Multicellular organismal development
Anatomical structure morphogenesis
Cell adhesion
Cell communication
Cell differentiation
Reproduction
Growth
Localization
Transport
Transcription
Biological regulation
Oxidation reduction
Immune response
Translation
GPCR protein signaling pathway
Signal transduction
Sensory perception
Multicellular organismal process
Response to stimulus
Oxidative phosphorylation
<3200
3201-10000
10001-22500
22501-53000
>53000
0
1
2-4
5-10
>10
<=210
211-315
316-460
461-720
>720
0
1
2
>2
Over-/under-representation strength grade:
6
4
2
0
-2
-4
-6

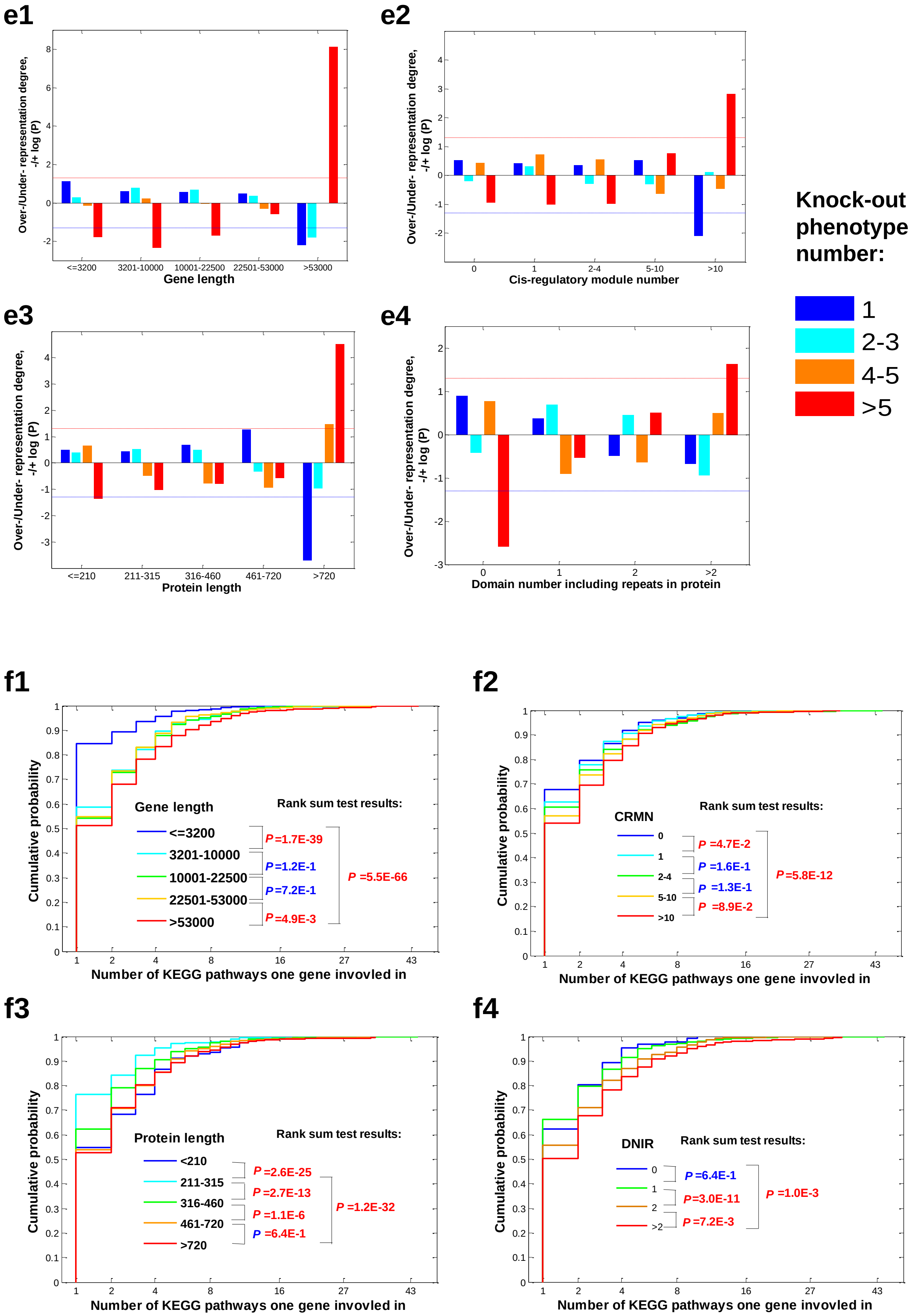

e1
Over-/Under- representation degree, -/+ log (P)
<=3200
3201-10000
10001-22500
22501-53000
>53000
Gene length
e2
Over-/Under- representation degree, -/+ log (P)
0
1
2-4
5-10
>10
Cis-regulatory module number
Knock-out phenotype number:
1
2-3
4-5
>5
e3
Over-/Under- representation degree, -/+ log (P)
<=210
211-315
316-460
461-720
>720
Protein length
e4
Over-/Under- representation degree, -/+ log (P)
0
1
2
>2
Domain number including repeats in protein
f1
Cumulative probability
Gene length
<=3200
3201-10000
10001-22500
22501-53000
>53000
Rank sum test results:
P =1.7E-39
P =1.2E-1
P =7.2E-1
P =4.9E-3
P =5.5E-66
Number of KEGG pathways one gene invovled in
f2
Cumulative probability
CRMN
0
1
2-4
5-10
>10
Rank sum test results:
P =4.7E-2
P =1.6E-1
P =1.3E-1
P =8.9E-2
P =5.8E-12
Number of KEGG pathways one gene invovled in
f3
Cumulative probability
Protein length
<210
211-315
316-460
461-720
>720
Rank sum test results:
P =2.6E-25
P =2.7E-13
P =1.1E-6
P =6.4E-1
P =1.2E-32
Number of KEGG pathways one gene invovled in
f4
Cumulative probability
DNIR
0
1
2
>2
Rank sum test results:
P =6.4E-1
P =3.0E-11
P =7.2E-3
P =1.0E-3
Number of KEGG pathways one gene invovled in

Figure S2

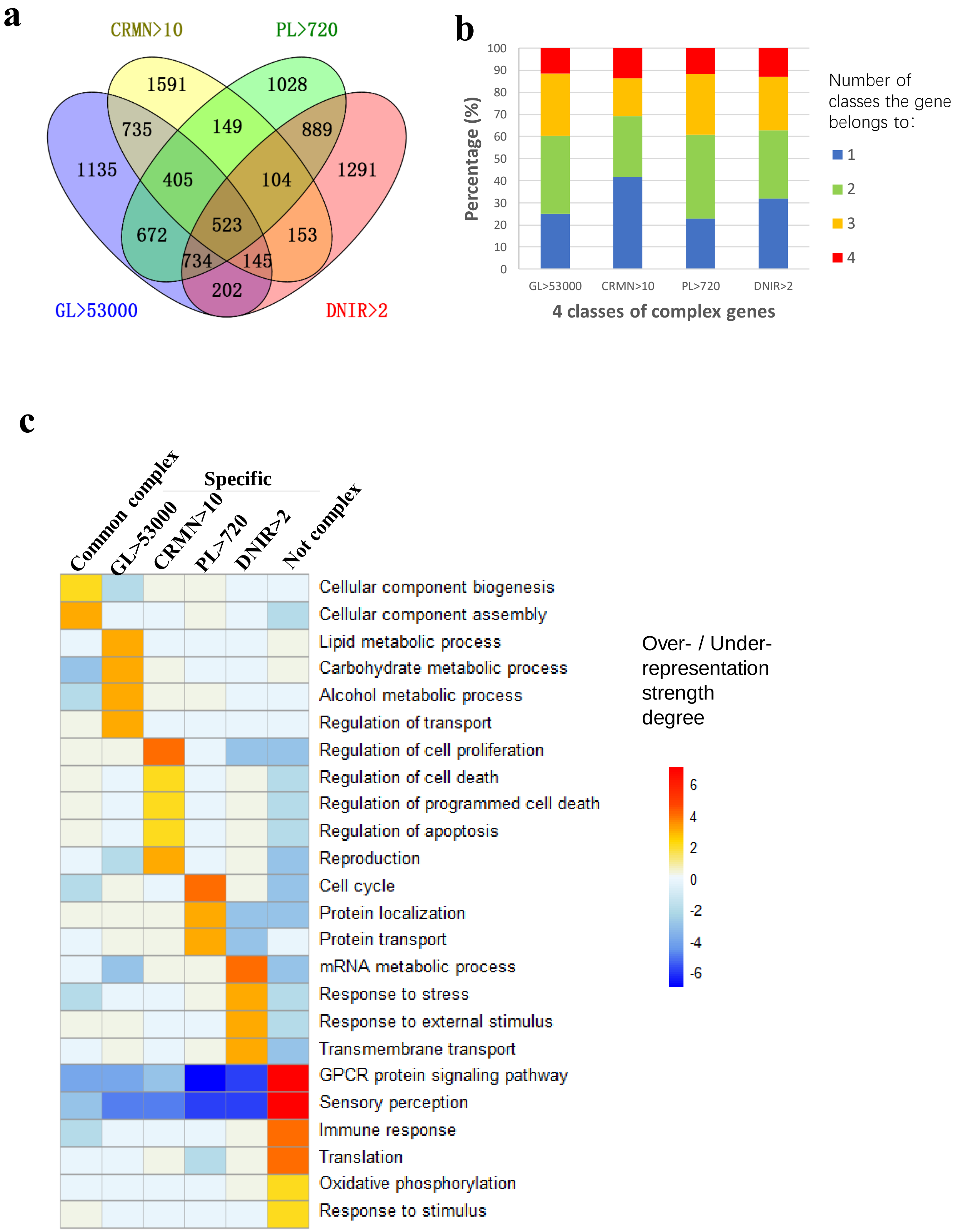
a
CRMN>10
PL>720
GL>53000
DNIR>2
1591
1028
735
149
889
1135
405
104
1291
523
672
153
734
145
202
b
Percentage (%)
100
90
80
70
60
50
40
30
20
10
0
GL>53000
CRMN>10
PL>720
DNIR>2
4 classes of complex genes
Number of classes the gene belongs to:
1
2
3
4
c
Common complex
Specific
GL>53000
CRMN>10
PL>720
DNIR>2
Not complex
Cellular component biogenesis
Cellular component assembly
Lipid metabolic process
Carbohydrate metabolic process
Alcohol metabolic process
Regulation of transport
Regulation of cell proliferation
Regulation of cell death
Regulation of programmed cell death
Regulation of apoptosis
Reproduction
Cell cycle
Protein localization
Protein transport
mRNA metabolic process
Response to stress
Response to external stimulus
Transmembrane transport
GPCR protein signaling pathway
Sensory perception
Immune response
Translation
Oxidative phosphorylation
Response to stimulus
Over- / Under-representation strength degree
6
4
2
0
-2
-4
-6

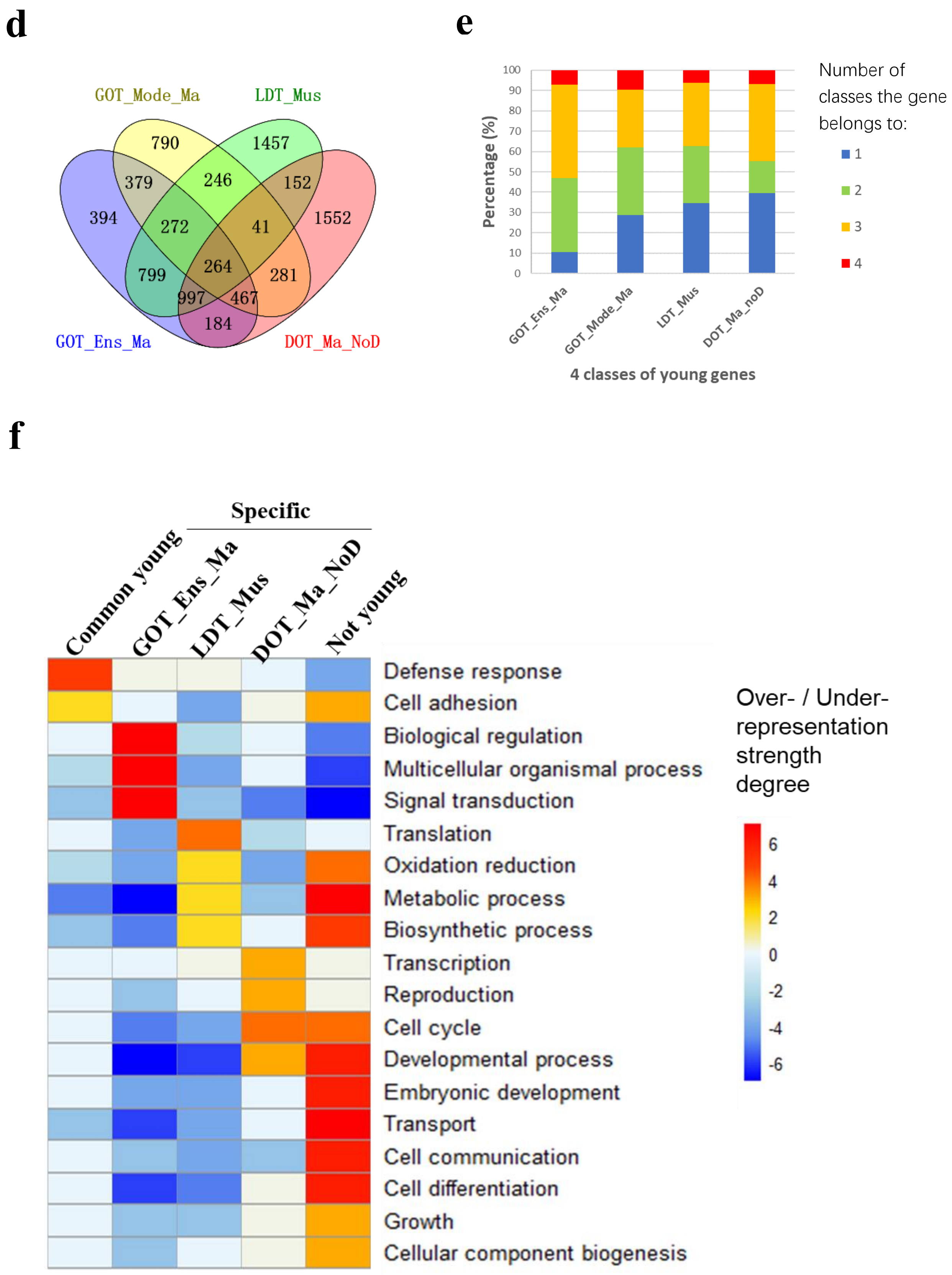
d
GOT_Mode_Ma
LDT_Mus
GOT_Ens_Ma
DOT_Ma_NoD
790
1457
379
246
152
394
272
41
1552
799
264
281
997
467
184
e
Percentage (%)
100
90
80
70
60
50
40
30
20
10
0
GOT_Ens_Ma
GOT_Mode_Ma
LDT_Mus
DOT_Ma_noD
4 classes of young genes
Number of classes the gene belongs to:
1
2
3
4
f
Specific
Common young
GOT_Ens_Ma
LDT_Mus
DOT_Ma_NoD
Not young
Defense response
Cell adhesion
Biological regulation
Multicellular organismal process
Signal transduction
Translation
Oxidation reduction
Metabolic process
Biosynthetic process
Transcription
Reproduction
Cell cycle
Developmental process
Embryonic development
Transport
Cell communication
Cell differentiation
Growth
Cellular component biogenesis
Over- / Under-representation strength degree
6
4
2
0
-2
-4
-6

Figure S3

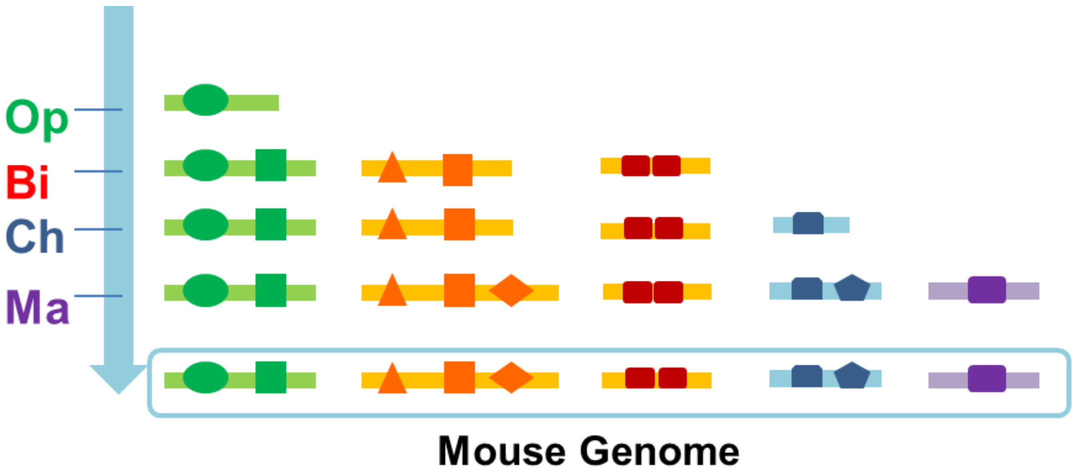

Op	Opisthokonta
Bi	Bilateria
Ch	Chordata
Ma	Mammalia

Figure S4

a

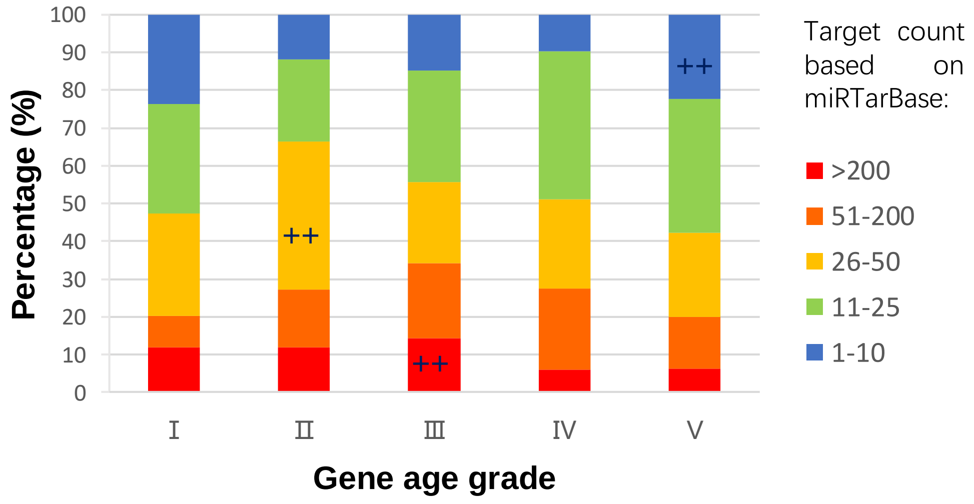

100
90
80
70
60
50
40
30
20
10
0
Percentage (%)
++
++
++
++
I
II
III
IV
V
Gene age grade
Target count based on miRTarBase:
>200
51-200
26-50
11-25
1-10

b

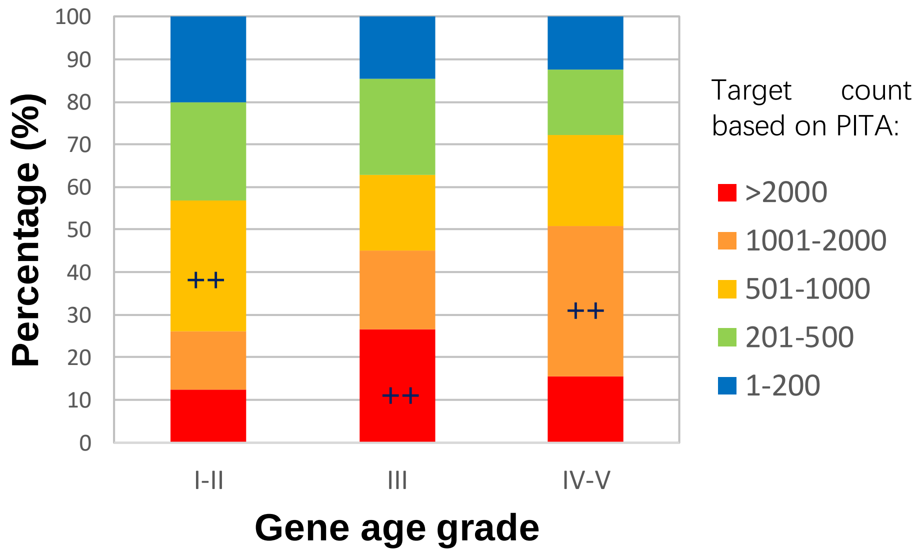

100
90
80
70
60
50
40
30
20
10
0
Percentage (%)
++
++
++
I-II
III
IV-V
Gene age grade
Target count based on PITA:
>2000
1001-2000
501-1000
201-500
1-200

Figure S5

a

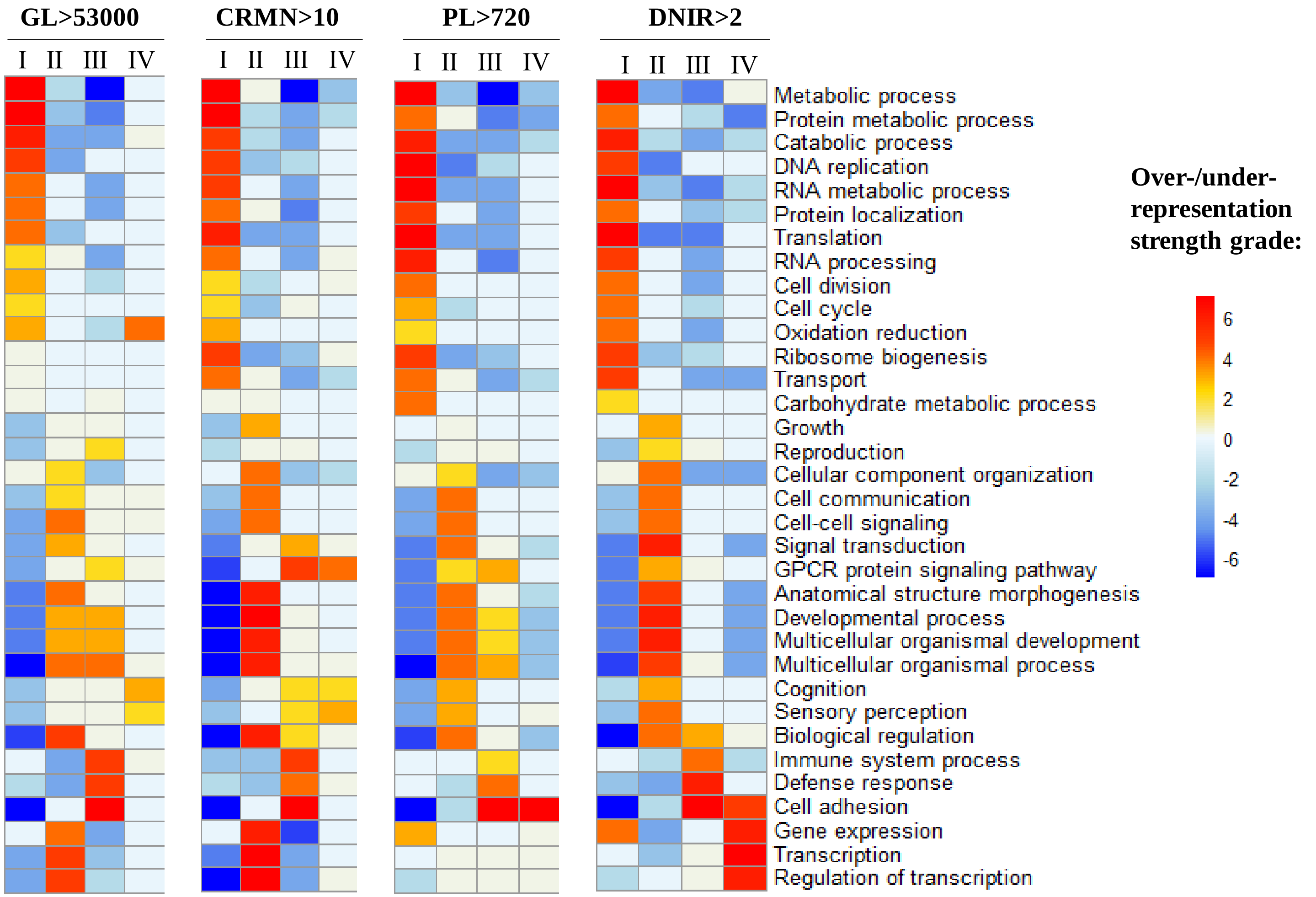
GL>53000
CRMN>10
PL>720
DNIR>2
I II III IV
Metabolic process
Protein metabolic process
Catabolic process
DNA replication
RNA metabolic process
Protein localization
Translation
RNA processing
Cell division
Cell cycle
Oxidation reduction
Ribosome biogenesis
Transport
Carbohydrate metabolic process
Growth
Reproduction
Cellular component organization
Cell communication
Cell-cell signaling
Signal transduction
GPCR protein signaling pathway
Anatomical structure morphogenesis
Developmental process
Multicellular organismal development
Multicellular organismal process
Cognition
Sensory perception
Biological regulation
Immune system process
Defense response
Cell adhesion
Gene expression
Transcription
Regulation of transcription
Over-/under-representation strength grade:
6
4
2
0
-2
-4
-6

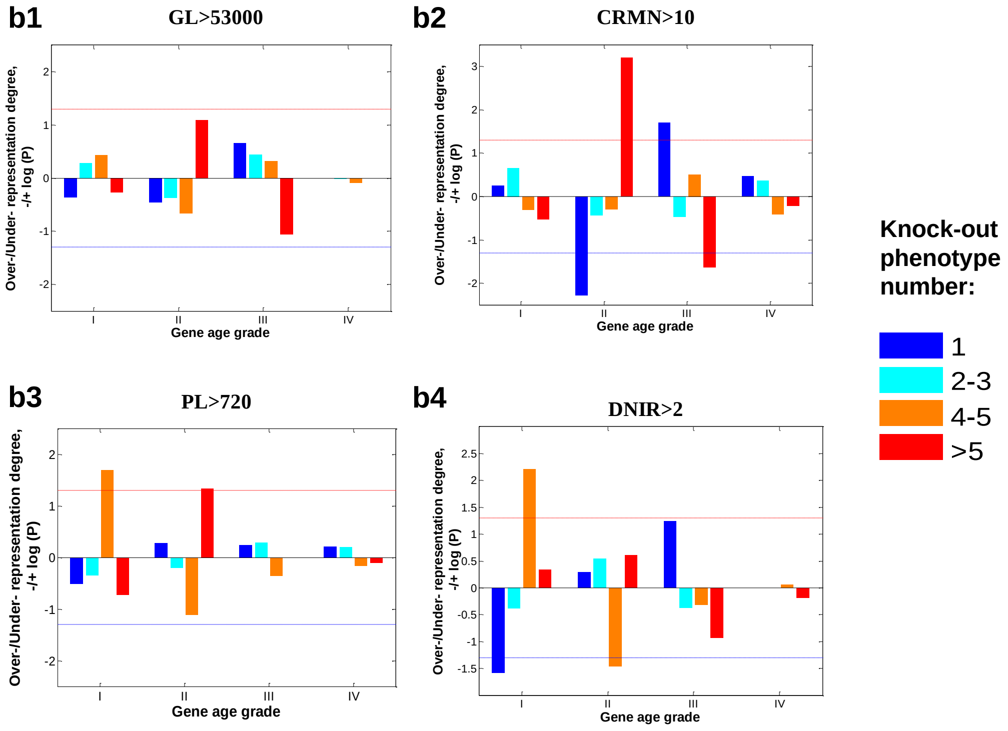
b1
GL>53000
Over-/Under- representation degree,
-/+ log (P)
Gene age grade
b2
CRMN>10
b3
PL>720
b4
DNIR>2
I
II
III
IV
Knock-out phenotype number:
1
2-3
4-5
>5

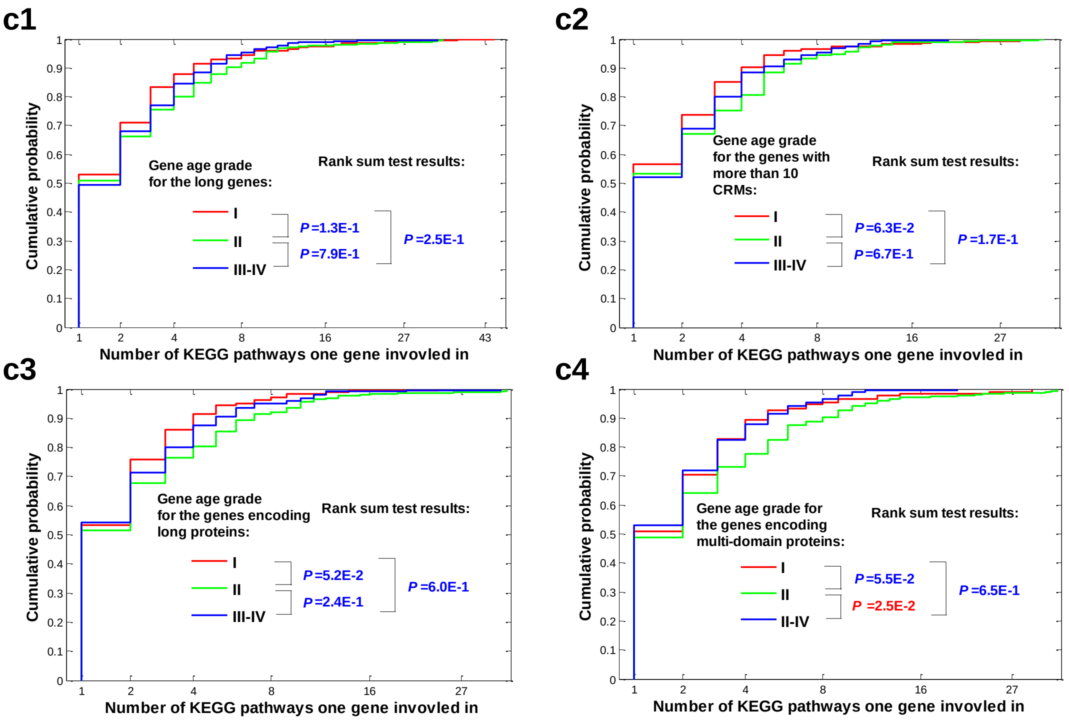
c1
Cumulative probability
Gene age grade for the long genes:
Rank sum test results:
I
II
III-IV
P =1.3E-1
P =7.9E-1
P =2.5E-1
Number of KEGG pathways one gene invovled in
c2
Gene age grade for the genes with more than 10 CRMs:
P =6.3E-2
P =6.7E-1
P =1.7E-1
c3
Gene age grade for the genes encoding long proteins:
P =5.2E-2
P =2.4E-1
P =6.0E-1
c4
Gene age grade for the genes encoding multi-domain proteins:
II-IV
P =5.5E-2
P =2.5E-2
P =6.5E-1

Figure S6

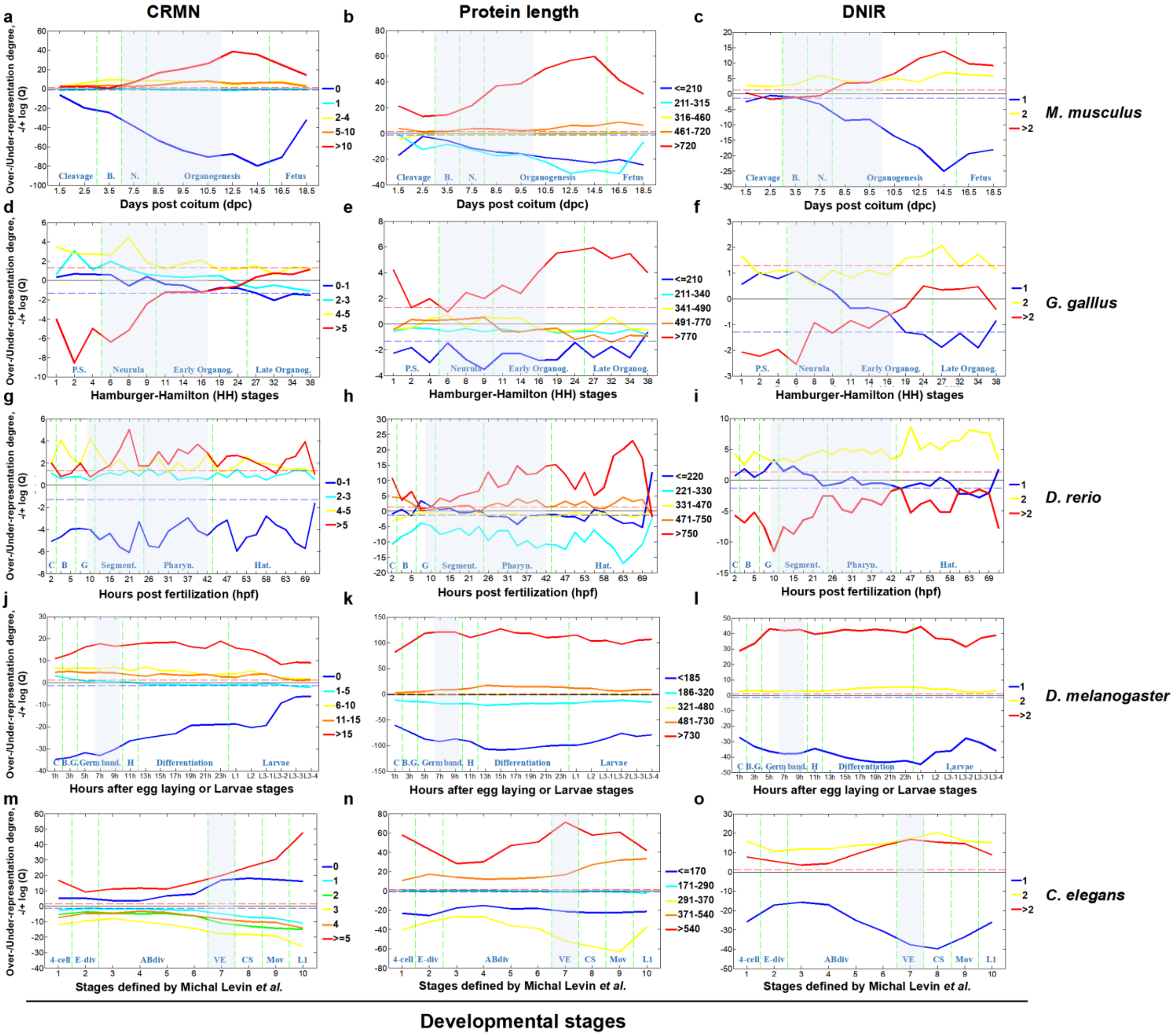

CRMN
Protein length
DNIR
M. musculus
G. galllus
D. rerio
D. melanogaster
C. elegans
Over-/Under-representation degree, -/+ log (Q)
Days post coitum (dpc)
Hamburger-Hamilton (HH) stages
Hours post fertilization (hpf)
Hours after egg laying or Larvae stages
Stages defined by Michal Levin et al.
Developmental stages

Figure S7

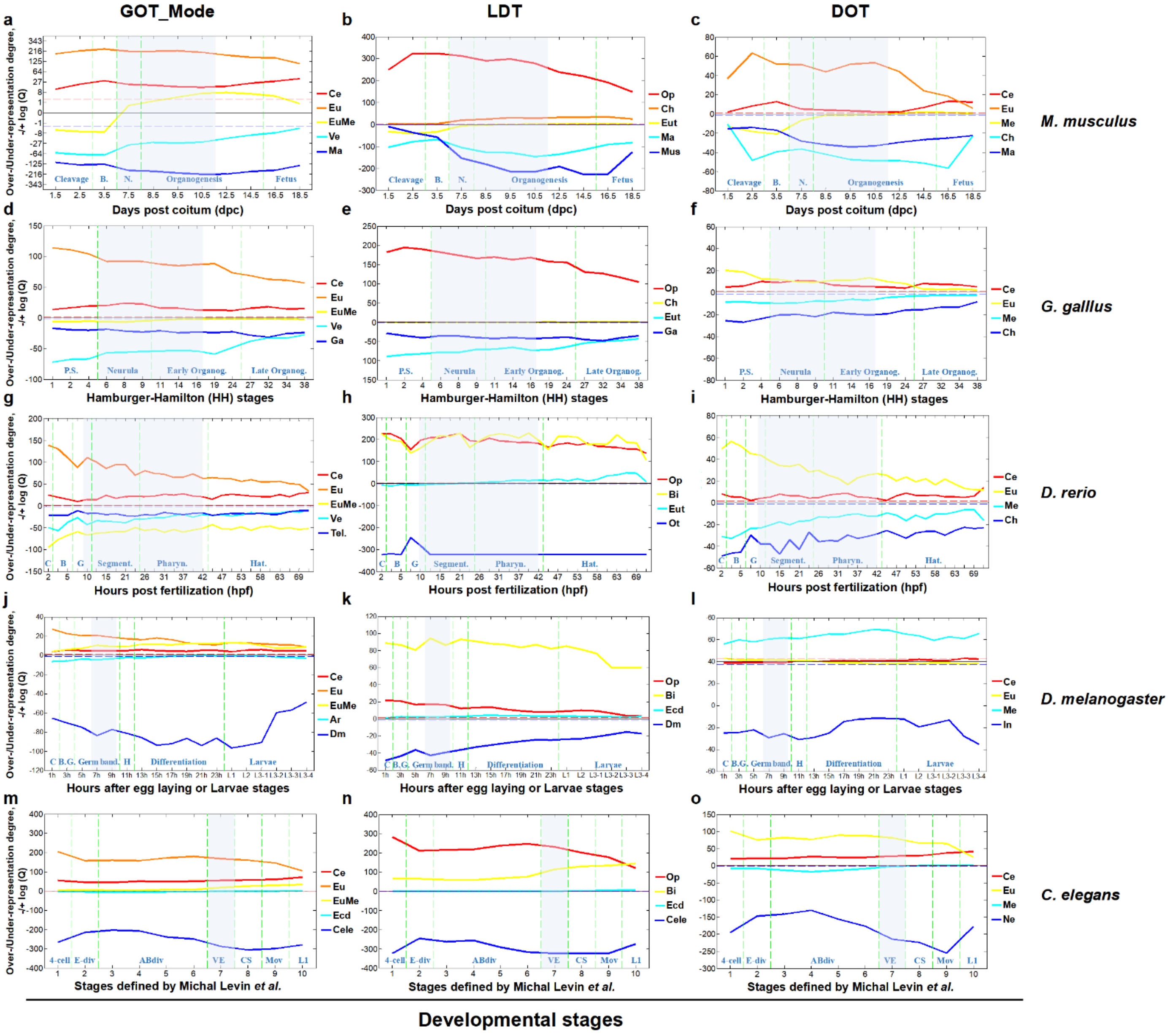
GOT_Mode
LDT
DOT
M. musculus
G. galllus
D. rerio
D. melanogaster
C. elegans
Over-/Under-representation degree, -/+ log (Q)
Days post coitum (dpc)
Hamburger-Hamilton (HH) stages
Hours post fertilization (hpf)
Hours after egg laying or Larvae stages
Stages defined by Michal Levin et al.
Developmental stages

Figure S8

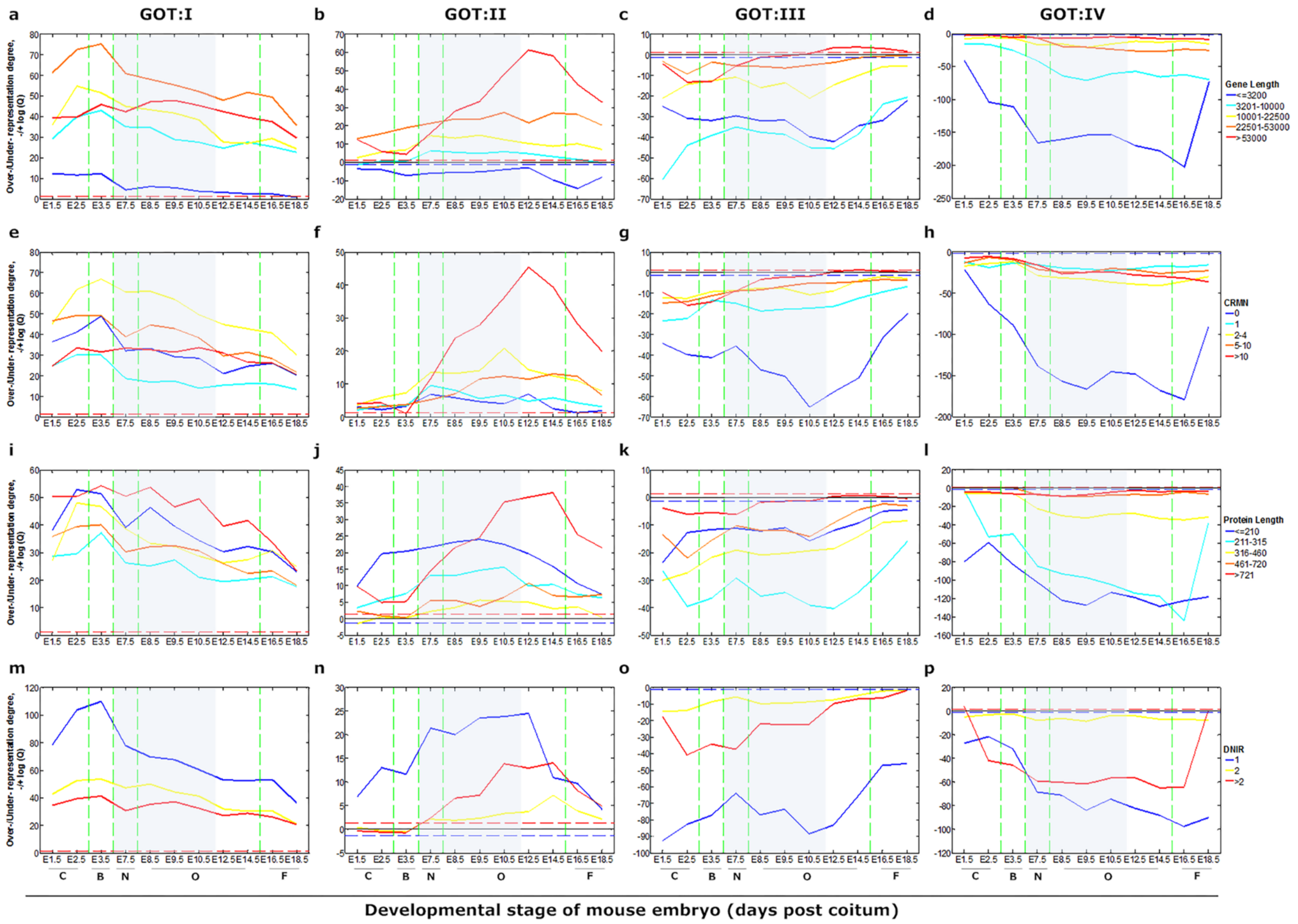

a
GOT:I
b
GOT:II
c
GOT:III
d
GOT:IV
e
f
g
h
i
j
k
l
m
n
o
p
Over-/Under- representation degree, -/+ log (Q)
Gene Length
<=3200
3201-10000
10001-22500
22501-53000
>53000
CRMN
0
1
2-4
5-10
>10
Protein Length
<=210
211-315
316-460
461-720
>721
DNIR
1
2
>2
E1.5 E2.5 E3.5 E7.5 E8.5 E9.5 E10.5 E12.5 E14.5 E16.5 E18.5
C B N O F
Developmental stage of mouse embryo (days post coitum)

Figure S9

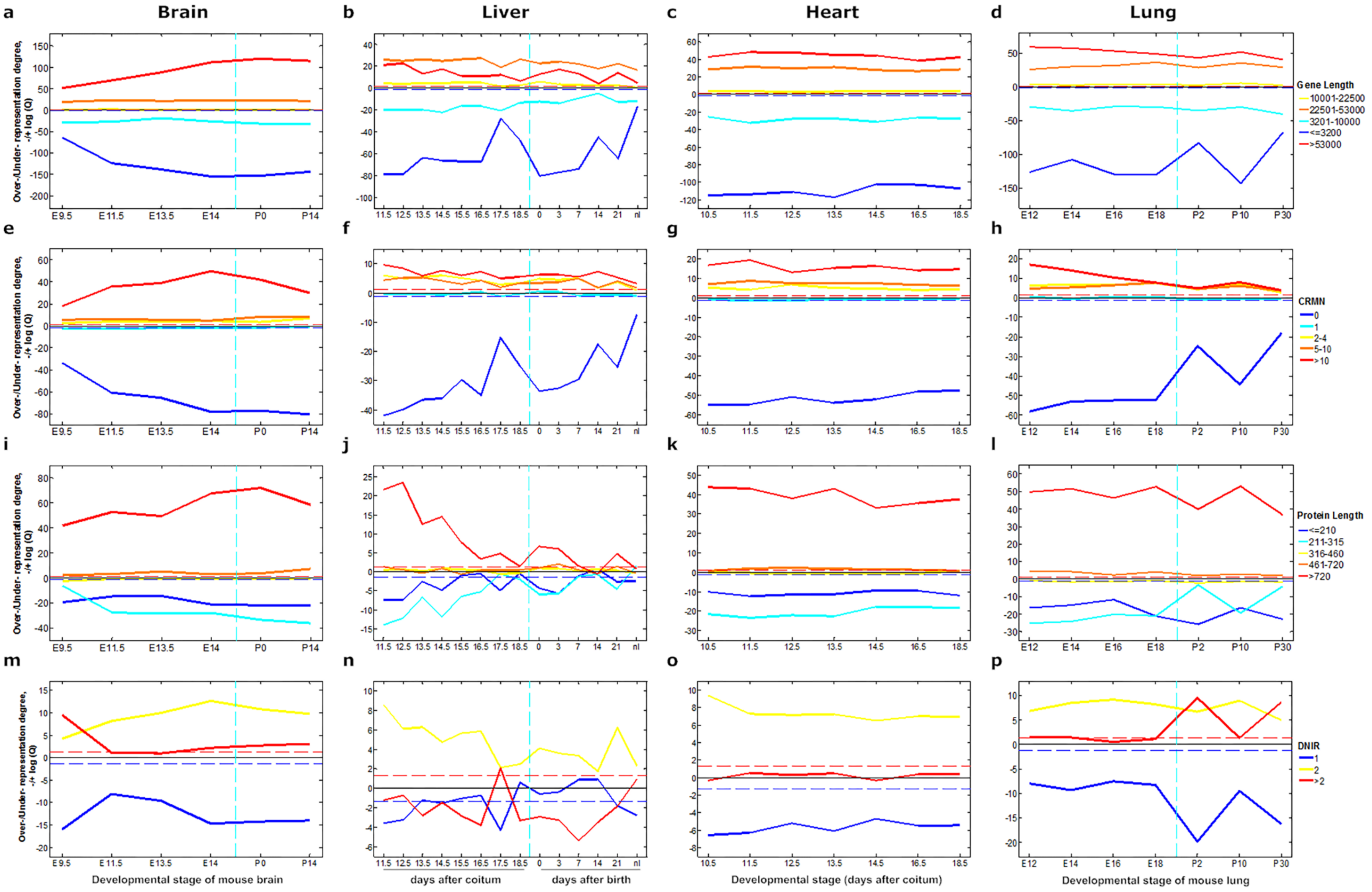
a
Brain
b
Liver
c
Heart
d
Lung
Over-/Under- representation degree, -/+ log (Q)
Gene Length
10001-22500
22501-53000
3201-10000
<=3200
>53000
CRMN
0
1
2-4
5-10
>10
Protein Length
<=210
211-315
316-460
461-720
>720
DNIR
1
2
>2
E9.5
E11.5
E13.5
E14
P0
P14
11.5 12.5 13.5 14.5 15.5 16.5 17.5 18.5 0 3 7 14 21 nl
10.5 11.5 12.5 13.5 14.5 16.5 18.5
E12 E14 E16 E18 P2 P10 P30
Developmental stage of mouse brain
days after coitum
days after birth
Developmental stage (days after coitum)
Developmental stage of mouse lung

Figure S10

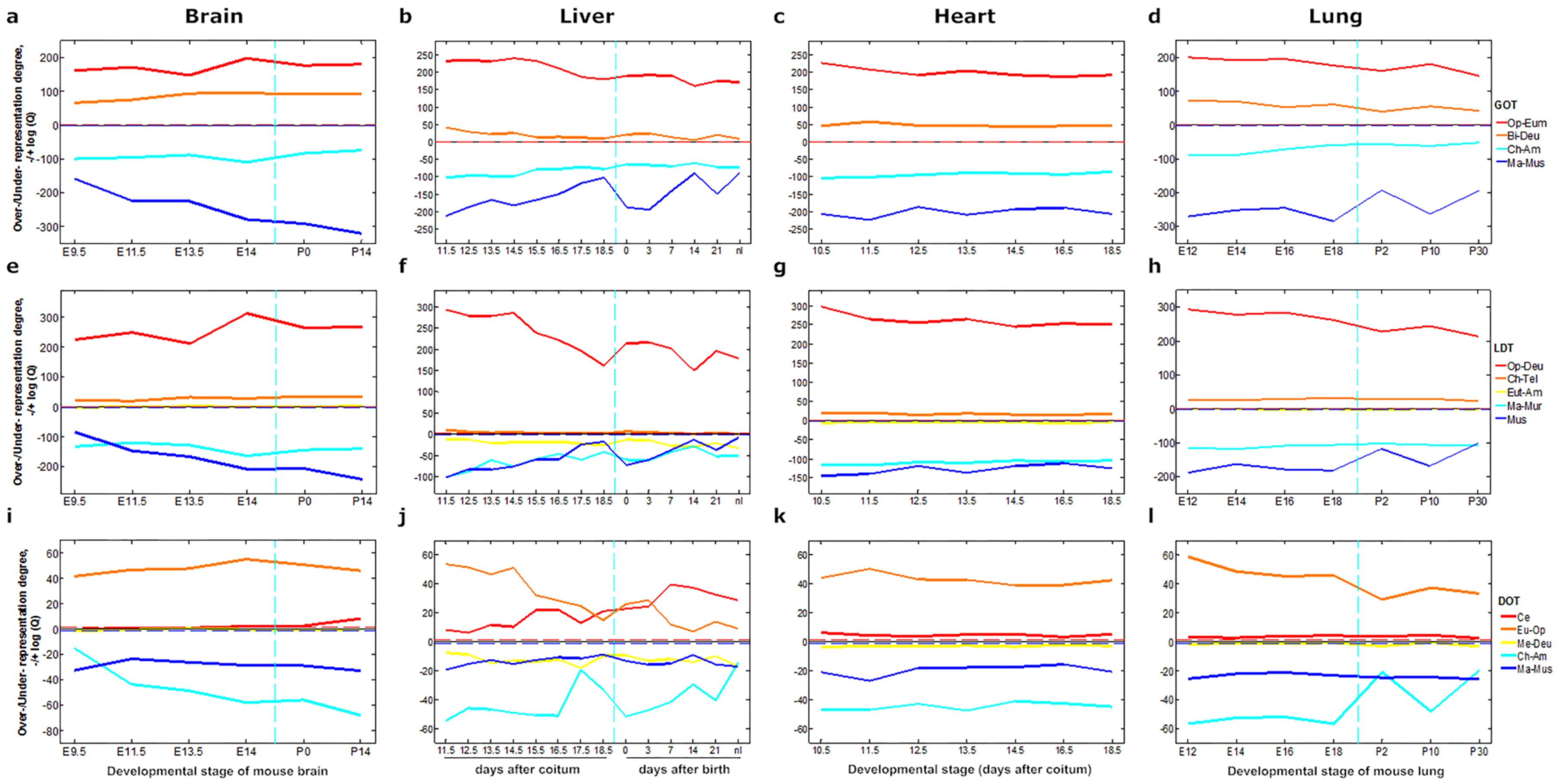
a
Brain
b
Liver
c
Heart
d
Lung
e
f
g
h
i
j
k
l
Over-/Under- representation degree, -/+ log (Q)
GOT
Op-Eum
Bi-Deu
Ch-Am
Ma-Mus
LDT
Op-Deu
Ch-Tel
Eut-Am
Ma-Mur
Mus
DOT
Ce
Eu-Op
Me-Deu
Ch-Am
Ma-Mus
E9.5
E11.5
E13.5
E14
P0
P14
E12
E14
E16
E18
P2
P10
P30
Developmental stage of mouse brain
days after coitum
days after birth
Developmental stage (days after coitum)
Developmental stage of mouse lung

Figure S11

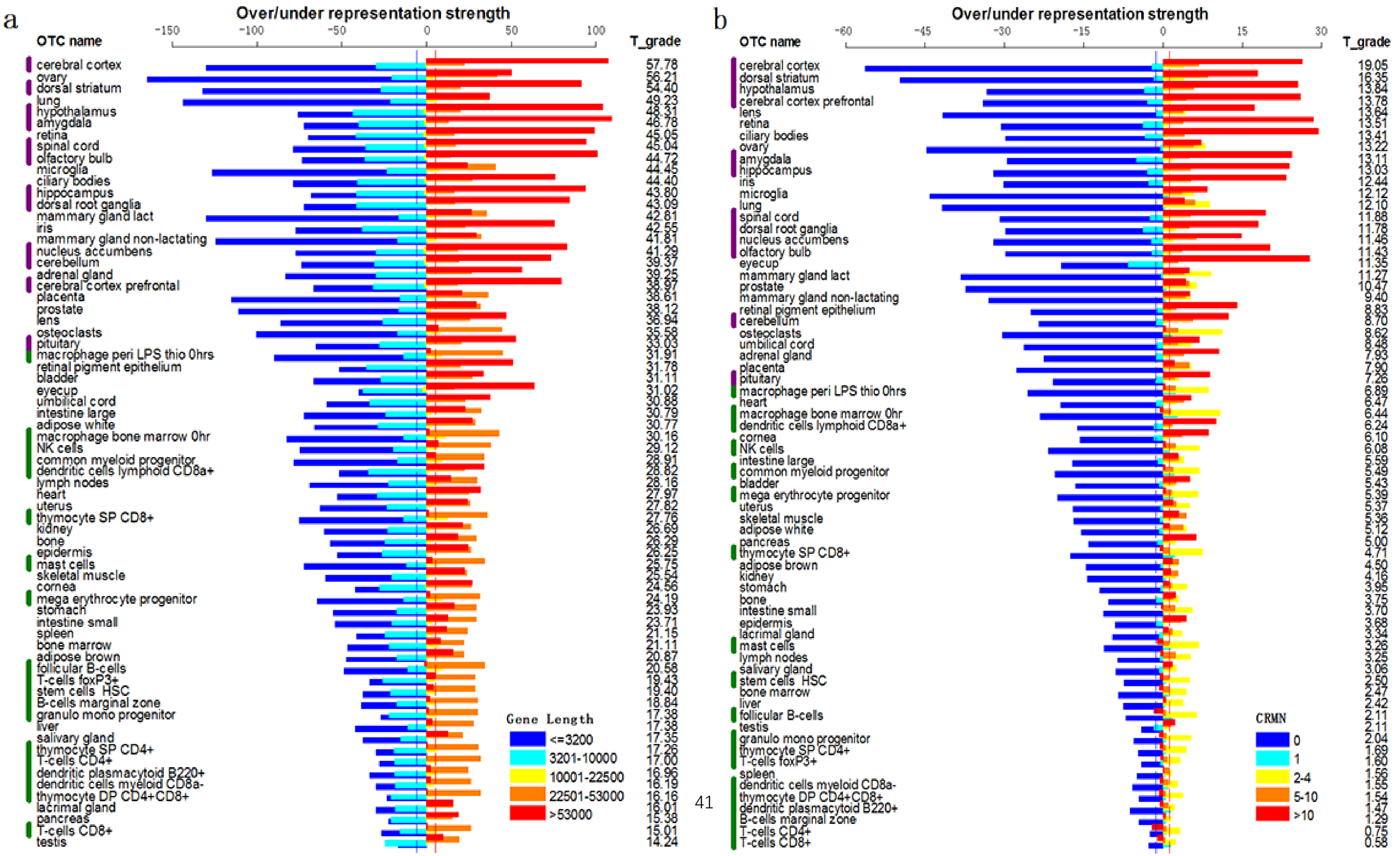
a
Over/under representation strength
OTC name
-150
-100
-50
0
50
100
T_grade
cerebral cortex 57.78
ovary 56.21
dorsal striatum 54.40
lung 49.23
hypothalamus 48.31
amygdala 46.78
retina 45.05
spinal cord 45.04
olfactory bulb 44.72
microglia 44.45
ciliary bodies 44.40
hippocampus 43.80
dorsal root ganglia 43.09
mammary gland lact 42.81
iris 42.55
mammary gland non-lactating 41.81
nucleus accumbens 41.29
cerebellum 39.37
adrenal gland 39.25
cerebral cortex prefrontal 38.97
placenta 38.61
prostate 38.12
lens 36.94
osteoclasts 35.58
pituitary 33.03
macrophage peri LPS thio 0hrs 31.91
retinal pigment epithelium 31.78
bladder 31.11
eyecup 31.02
umbilical cord 30.88
intestine large 30.79
adipose white 30.77
macrophage bone marrow 0hr 30.16
NK cells 29.12
common myeloid progenitor 28.91
dendritic cells lymphoid CD8a+ 28.82
lymph nodes 28.16
heart 27.97
uterus 27.82
thymocyte SP CD8+ 27.76
kidney 26.69
bone 26.29
epidermis 26.25
mast cells 25.75
skeletal muscle 25.54
cornea 24.56
mega erythrocyte progenitor 24.19
stomach 23.93
intestine small 23.71
spleen 21.15
bone marrow 21.11
adipose brown 20.87
follicular B-cells 20.58
T-cells foxP3+ 19.43
stem cells HSC 19.40
B-cells marginal zone 18.84
granulo mono progenitor 17.38
liver 17.38
salivary gland 17.35
thymocyte SP CD4+ 17.26
T-cells CD4+ 17.00
dendritic plasmacytoid B220+ 16.96
dendritic cells myeloid CD8a- 16.19
thymocyte DP CD4+CD8+ 16.16
lacrimal gland 16.01
pancreas 15.38
T-cells CD8+ 15.01
testis 14.24
Gene Length
<=3200
3201-10000
10001-22500
22501-53000
>53000
b
Over/under representation strength
OTC name
-60
-45
-30
-15
0
15
30
T_grade
cerebral cortex 19.05
dorsal striatum 16.35
hypothalamus 13.84
cerebral cortex prefrontal 13.78
lens 13.64
retina 13.51
ciliary bodies 13.41
ovary 13.22
amygdala 13.11
hippocampus 13.03
iris 12.44
microglia 12.12
lung 12.10
spinal cord 11.88
dorsal root ganglia 11.78
nucleus accumbens 11.46
olfactory bulb 11.43
eyecup 11.35
mammary gland lact 11.27
prostate 10.47
mammary gland non-lactating 9.40
retinal pigment epithelium 8.83
cerebellum 8.70
osteoclasts 8.62
umbilical cord 8.48
adrenal gland 7.93
placenta 7.90
pituitary 7.26
macrophage peri LPS thio 0hrs 6.89
heart 6.47
macrophage bone marrow 0hr 6.44
dendritic cells lymphoid CD8a+ 6.24
cornea 6.10
NK cells 6.08
intestine large 5.59
common myeloid progenitor 5.49
bladder 5.43
mega erythrocyte progenitor 5.39
uterus 5.37
skeletal muscle 5.36
adipose white 5.12
pancreas 5.00
thymocyte SP CD8+ 4.71
adipose brown 4.50
kidney 4.16
stomach 3.95
bone 3.75
intestine small 3.70
epidermis 3.68
lacrimal gland 3.34
mast cells 3.26
lymph nodes 3.25
salivary gland 3.06
stem cells HSC 2.50
bone marrow 2.47
liver 2.42
follicular B-cells 2.11
testis 2.11
granulo mono progenitor 2.04
thymocyte SP CD4+ 1.69
T-cells foxP3+ 1.60
spleen 1.56
dendritic cells myeloid CD8a- 1.55
thymocyte DP CD4+CD8+ 1.54
dendritic plasmacytoid B220+ 1.47
B-cells marginal zone 1.29
T-cells CD4+ 0.75
T-cells CD8+ 0.58
CRMN
0
1
2-4
5-10
>10

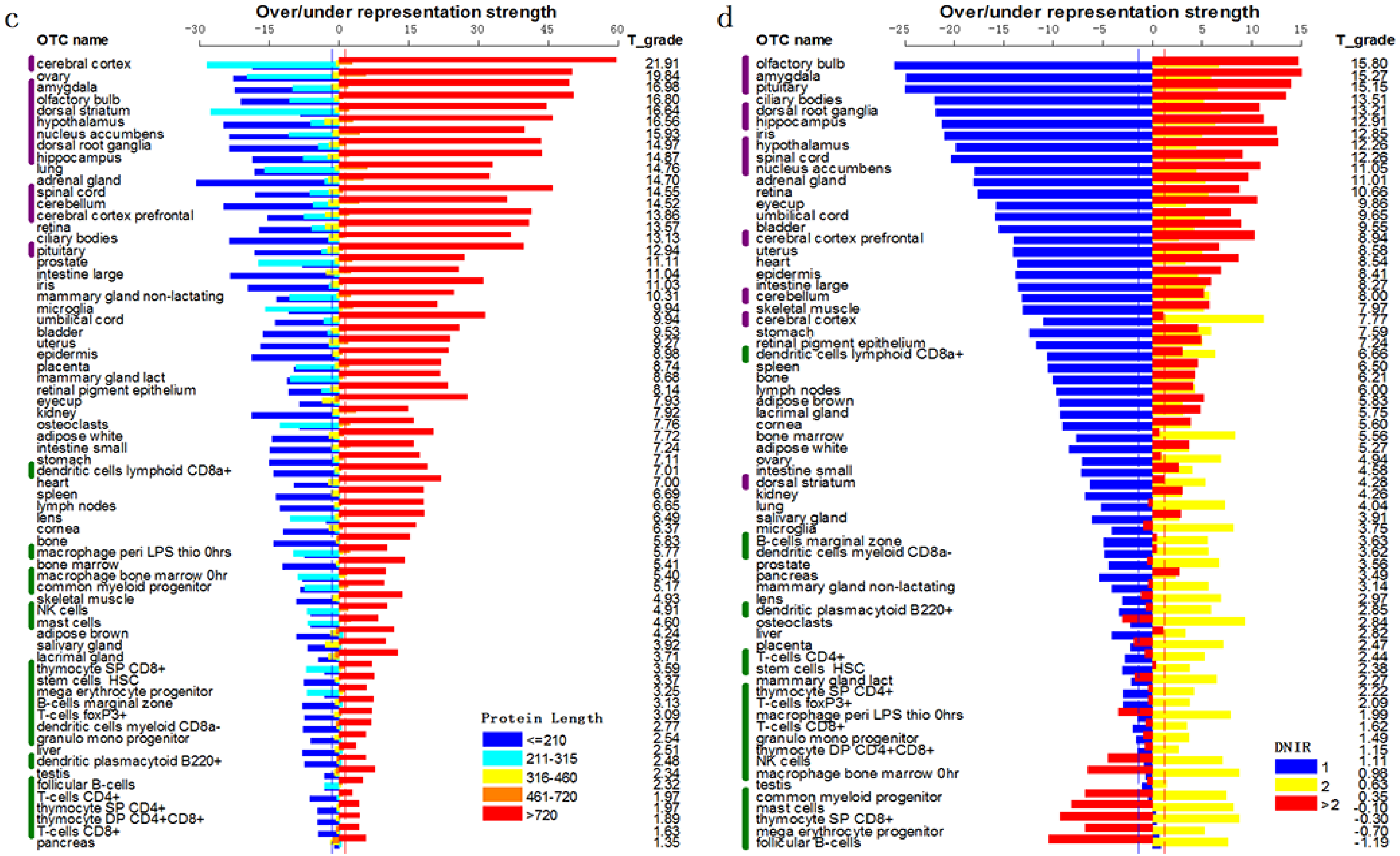
c
Over/under representation strength
OTC name
-30 -15 0 15 30 45 60
T_grade
cerebral cortex 21.91
ovary 19.84
amygdala 16.98
olfactory bulb 16.80
dorsal striatum 16.64
hypothalamus 16.56
nucleus accumbens 15.93
dorsal root ganglia 14.97
hippocampus 14.87
lung 14.76
adrenal gland 14.70
spinal cord 14.55
cerebellum 14.52
cerebral cortex prefrontal 13.86
retina 13.57
ciliary bodies 13.13
pituitary 12.94
prostate 11.11
intestine large 11.04
iris 11.03
mammary gland non-lactating 10.31
microglia 9.94
umbilical cord 9.94
bladder 9.53
uterus 9.27
epidermis 8.98
placenta 8.74
mammary gland lact 8.68
retinal pigment epithelium 8.14
eyecup 7.93
kidney 7.92
osteoclasts 7.76
adipose white 7.72
intestine small 7.24
stomach 7.11
dendritic cells lymphoid CD8a+ 7.01
heart 7.00
spleen 6.69
lymph nodes 6.65
lens 6.49
cornea 6.37
bone 5.83
macrophage peri LPS thio 0hrs 5.77
bone marrow 5.41
macrophage bone marrow 0hr 5.40
common myeloid progenitor 5.17
skeletal muscle 4.93
NK cells 4.91
mast cells 4.60
adipose brown 4.24
salivary gland 3.92
lacrimal gland 3.71
thymocyte SP CD8+ 3.59
stem cells HSC 3.37
mega erythrocyte progenitor 3.25
B-cells marginal zone 3.13
T-cells foxP3+ 3.09
dendritic cells myeloid CD8a- 2.77
granulo mono progenitor 2.54
liver 2.51
dendritic plasmacytoid B220+ 2.48
testis 2.34
follicular B-cells 2.32
T-cells CD4+ 1.97
thymocyte SP CD4+ 1.97
thymocyte DP CD4+CD8+ 1.89
T-cells CD8+ 1.63
pancreas 1.35
Protein Length
<=210
211-315
316-460
461-720
>720
d
Over/under representation strength
OTC name
-25 -20 -15 -10 -5 0 5 10 15
T_grade
olfactory bulb 15.80
amygdala 15.27
pituitary 15.15
ciliary bodies 13.51
dorsal root ganglia 13.21
hippocampus 12.91
iris 12.85
hypothalamus 12.26
spinal cord 12.26
nucleus accumbens 11.05
adrenal gland 11.01
retina 10.66
eyecup 9.86
umbilical cord 9.65
bladder 9.55
cerebral cortex prefrontal 8.94
uterus 8.58
heart 8.54
epidermis 8.41
intestine large 8.27
cerebellum 8.00
skeletal muscle 7.97
cerebral cortex 7.77
stomach 7.59
retinal pigment epithelium 7.24
dendritic cells lymphoid CD8a+ 6.66
spleen 6.50
bone 6.21
lymph nodes 6.00
adipose brown 5.83
lacrimal gland 5.75
cornea 5.60
bone marrow 5.56
adipose white 5.27
ovary 4.94
intestine small 4.58
dorsal striatum 4.28
kidney 4.26
lung 4.04
salivary gland 3.91
microglia 3.75
B-cells marginal zone 3.63
dendritic cells myeloid CD8a- 3.62
prostate 3.56
pancreas 3.49
mammary gland non-lactating 3.14
lens 2.97
dendritic plasmacytoid B220+ 2.85
osteoclasts 2.84
liver 2.82
placenta 2.47
T-cells CD4+ 2.44
stem cells HSC 2.38
mammary gland lact 2.27
thymocyte SP CD4+ 2.22
T-cells foxP3+ 2.09
macrophage peri LPS thio 0hrs 1.99
T-cells CD8+ 1.62
granulo mono progenitor 1.49
thymocyte DP CD4+CD8+ 1.15
NK cells 1.11
macrophage bone marrow 0hr 0.98
testis 0.63
common myeloid progenitor 0.35
mast cells -0.10
thymocyte SP CD8+ -0.30
mega erythrocyte progenitor -0.70
follicular B-cells -1.19
DNIR
1
2
>2

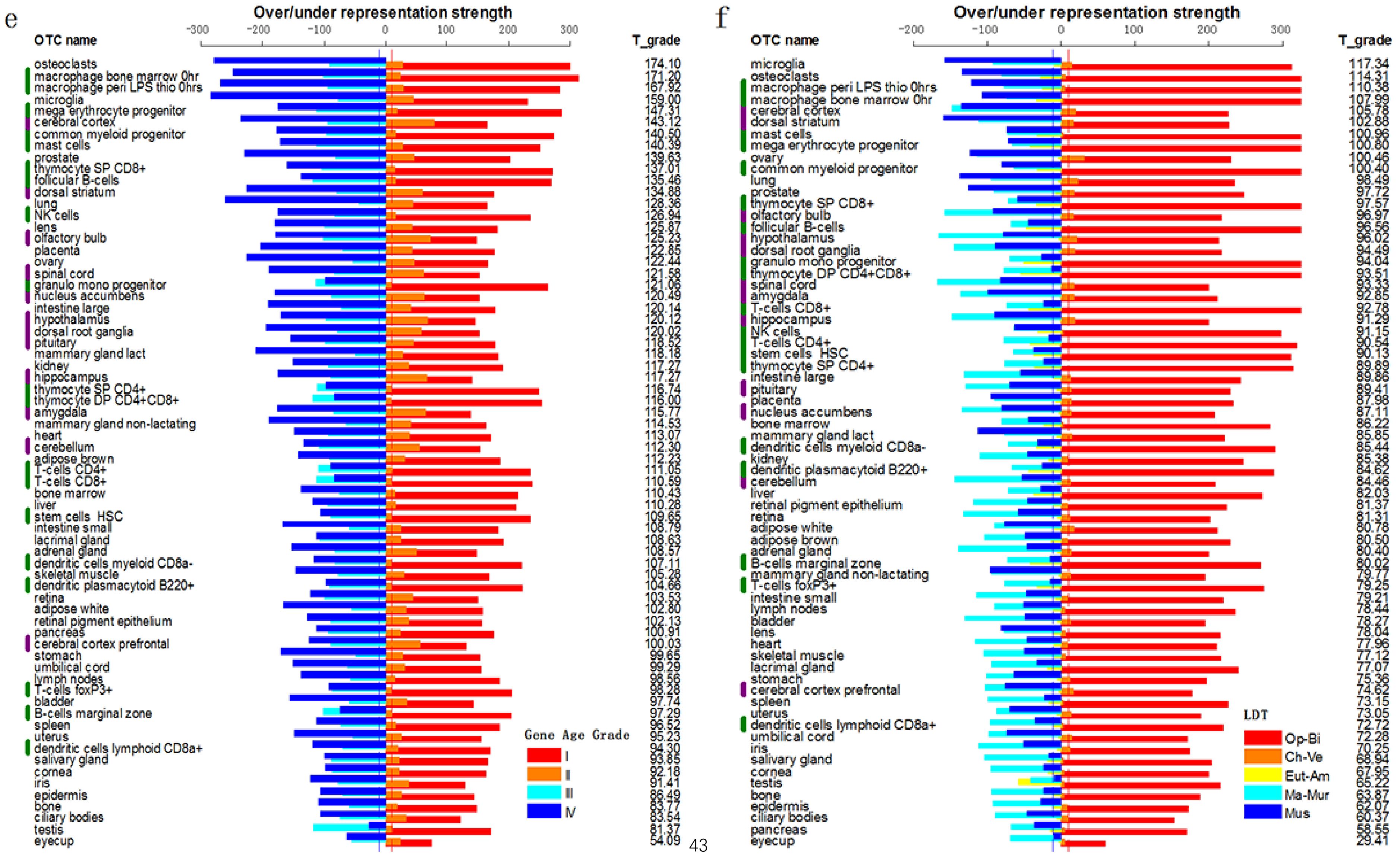
e
Over/under representation strength
OTC name
-300 -200 -100 0 100 200 300
T_grade
osteoclasts 174.10
macrophage bone marrow 0hr 171.20
macrophage peri LPS thio 0hrs 167.92
microglia 159.00
mega erythrocyte progenitor 147.31
cerebral cortex 143.12
common myeloid progenitor 140.50
mast cells 140.39
prostate 139.63
thymocyte SP CD8+ 137.01
follicular B-cells 135.46
dorsal striatum 134.88
lung 128.36
NK cells 126.94
lens 125.87
olfactory bulb 125.23
placenta 122.85
ovary 122.44
spinal cord 121.58
granulo mono progenitor 121.06
nucleus accumbens 120.49
intestine large 120.14
hypothalamus 120.12
dorsal root ganglia 120.02
pituitary 118.52
mammary gland lact 118.18
kidney 117.27
hippocampus 117.27
thymocyte SP CD4+ 116.74
thymocyte DP CD4+CD8+ 116.00
amygdala 115.77
mammary gland non-lactating 114.53
heart 113.07
cerebellum 112.30
adipose brown 112.23
T-cells CD4+ 111.05
T-cells CD8+ 110.59
bone marrow 110.43
liver 110.28
stem cells HSC 109.65
intestine small 108.79
lacrimal gland 108.63
adrenal gland 108.57
dendritic cells myeloid CD8a- 107.11
skeletal muscle 105.28
dendritic plasmacytoid B220+ 104.66
retina 103.53
adipose white 102.80
retinal pigment epithelium 102.13
pancreas 100.91
cerebral cortex prefrontal 100.03
stomach 99.65
umbilical cord 99.29
lymph nodes 98.56
T-cells foxP3+ 98.28
bladder 97.74
B-cells marginal zone 97.29
spleen 96.52
uterus 95.23
dendritic cells lymphoid CD8a+ 94.30
salivary gland 93.85
cornea 92.18
iris 91.41
epidermis 86.49
bone 83.77
ciliary bodies 83.54
testis 81.37
eyecup 54.09
Gene Age Grade
I
II
III
IV
f
Over/under representation strength
OTC name
-200 -100 0 100 200 300
T_grade
microglia 117.34
osteoclasts 114.31
macrophage peri LPS thio 0hrs 110.38
macrophage bone marrow 0hr 107.99
cerebral cortex 105.78
dorsal striatum 102.88
mast cells 100.96
mega erythrocyte progenitor 100.80
ovary 100.46
common myeloid progenitor 100.40
lung 98.49
prostate 97.72
thymocyte SP CD8+ 97.57
olfactory bulb 96.97
follicular B-cells 96.56
hypothalamus 96.02
dorsal root ganglia 94.49
granulo mono progenitor 94.04
thymocyte DP CD4+CD8+ 93.51
spinal cord 93.33
amygdala 92.85
T-cells CD8+ 92.78
hippocampus 91.29
NK cells 91.15
T-cells CD4+ 90.54
stem cells HSC 90.13
thymocyte SP CD4+ 89.89
intestine large 89.86
pituitary 89.41
placenta 87.98
nucleus accumbens 87.11
bone marrow 86.22
mammary gland lact 85.85
dendritic cells myeloid CD8a- 85.44
kidney 85.38
dendritic plasmacytoid B220+ 84.62
cerebellum 84.46
liver 82.03
retinal pigment epithelium 81.37
retina 81.31
adipose white 80.78
adipose brown 80.50
adrenal gland 80.40
B-cells marginal zone 80.02
mammary gland non-lactating 79.77
T-cells foxP3+ 79.25
intestine small 79.21
lymph nodes 78.44
bladder 78.27
lens 78.04
heart 77.96
skeletal muscle 77.12
lacrimal gland 77.07
stomach 75.36
cerebral cortex prefrontal 74.62
spleen 73.15
uterus 73.05
dendritic cells lymphoid CD8a+ 72.72
umbilical cord 72.28
iris 70.25
salivary gland 68.94
cornea 67.95
testis 65.22
bone 63.87
epidermis 62.07
ciliary bodies 60.37
pancreas 58.55
eyecup 29.41
LDT
Op-Bi
Ch-Ve
Eut-Am
Ma-Mur
Mus

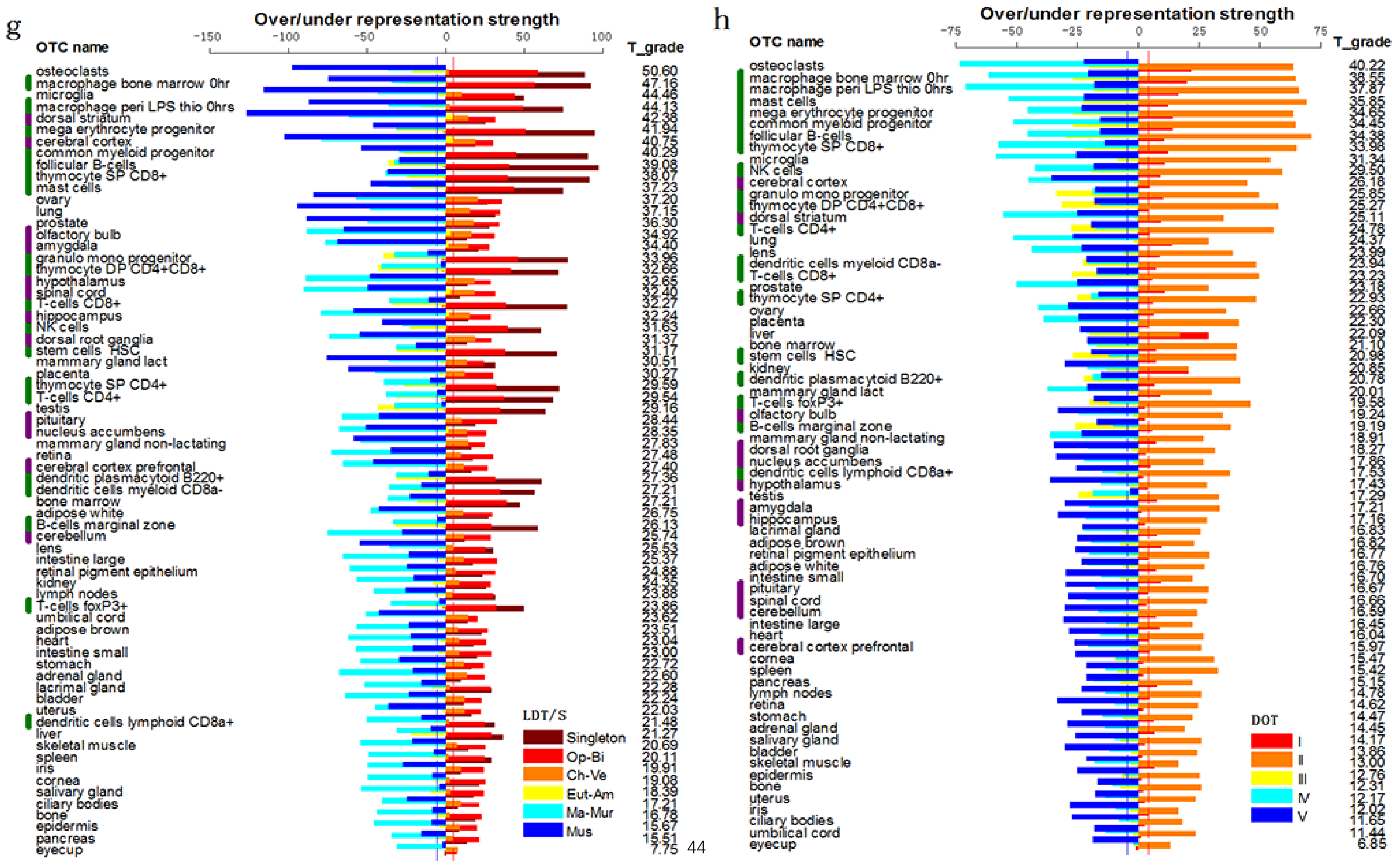
g
Over/under representation strength
OTC name
-150 -100 -50 0 50 100
T_grade
osteoclasts
macrophage bone marrow 0hr
microglia
macrophage peri LPS thio 0hrs
dorsal striatum
mega erythrocyte progenitor
cerebral cortex
common myeloid progenitor
follicular B-cells
thymocyte SP CD8+
mast cells
ovary
lung
prostate
olfactory bulb
amygdala
granulo mono progenitor
thymocyte DP CD4+CD8+
hypothalamus
spinal cord
T-cells CD8+
hippocampus
NK cells
dorsal root ganglia
stem cells HSC
mammary gland lact
placenta
thymocyte SP CD4+
T-cells CD4+
testis
pituitary
nucleus accumbens
mammary gland non-lactating
retina
cerebral cortex prefrontal
dendritic plasmacytoid B220+
dendritic cells myeloid CD8a-
bone marrow
adipose white
B-cells marginal zone
cerebellum
lens
intestine large
retinal pigment epithelium
kidney
lymph nodes
T-cells foxP3+
umbilical cord
adipose brown
heart
intestine small
stomach
adrenal gland
lacrimal gland
bladder
uterus
dendritic cells lymphoid CD8a+
liver
skeletal muscle
spleen
iris
cornea
salivary gland
ciliary bodies
bone
epidermis
pancreas
eyecup
50.60
47.16
44.46
44.13
42.38
41.94
40.75
40.29
39.08
38.07
37.23
37.20
37.15
36.30
34.92
34.40
33.96
32.66
32.65
32.40
32.27
32.24
31.63
31.37
31.17
30.51
30.27
29.59
29.54
29.16
28.44
28.35
27.83
27.48
27.40
27.36
27.21
27.21
26.75
26.13
25.74
25.53
25.37
24.88
24.35
23.88
23.86
23.62
23.51
23.04
23.00
22.72
22.60
22.28
22.24
22.03
21.48
21.27
20.69
20.11
19.91
19.08
18.39
17.21
16.78
15.67
15.51
7.75
LDT/S
Singleton
Op-Bi
Ch-Ve
Eut-Am
Ma-Mur
Mus
h
Over/under representation strength
OTC name
-75 -50 -25 0 25 50 75
T_grade
osteoclasts
macrophage bone marrow 0hr
macrophage peri LPS thio 0hrs
mast cells
mega erythrocyte progenitor
common myeloid progenitor
follicular B-cells
thymocyte SP CD8+
microglia
NK cells
cerebral cortex
granulo mono progenitor
thymocyte DP CD4+CD8+
dorsal striatum
T-cells CD4+
lung
lens
dendritic cells myeloid CD8a-
T-cells CD8+
prostate
thymocyte SP CD4+
ovary
placenta
liver
bone marrow
stem cells HSC
kidney
dendritic plasmacytoid B220+
mammary gland lact
T-cells foxP3+
olfactory bulb
B-cells marginal zone
mammary gland non-lactating
dorsal root ganglia
nucleus accumbens
dendritic cells lymphoid CD8a+
hypothalamus
testis
amygdala
hippocampus
lacrimal gland
adipose brown
retinal pigment epithelium
adipose white
intestine small
pituitary
spinal cord
cerebellum
intestine large
heart
cerebral cortex prefrontal
cornea
spleen
pancreas
lymph nodes
retina
stomach
adrenal gland
salivary gland
bladder
skeletal muscle
epidermis
bone
uterus
iris
ciliary bodies
umbilical cord
eyecup
40.22
38.55
37.87
35.85
34.65
34.45
34.38
33.98
31.34
29.50
26.18
25.85
25.27
25.11
24.78
24.37
23.99
23.94
23.23
23.18
22.93
22.66
22.30
22.09
21.10
20.98
20.85
20.78
20.01
19.58
19.24
19.19
18.91
18.27
17.86
17.53
17.43
17.29
17.21
17.16
16.83
16.82
16.77
16.76
16.70
16.67
16.66
16.59
16.45
16.04
15.97
15.47
15.42
15.15
14.78
14.62
14.47
14.45
14.17
13.86
13.00
12.76
12.31
12.17
12.02
11.65
11.44
6.85
DOT
I
II
III
IV
V

Figure S12

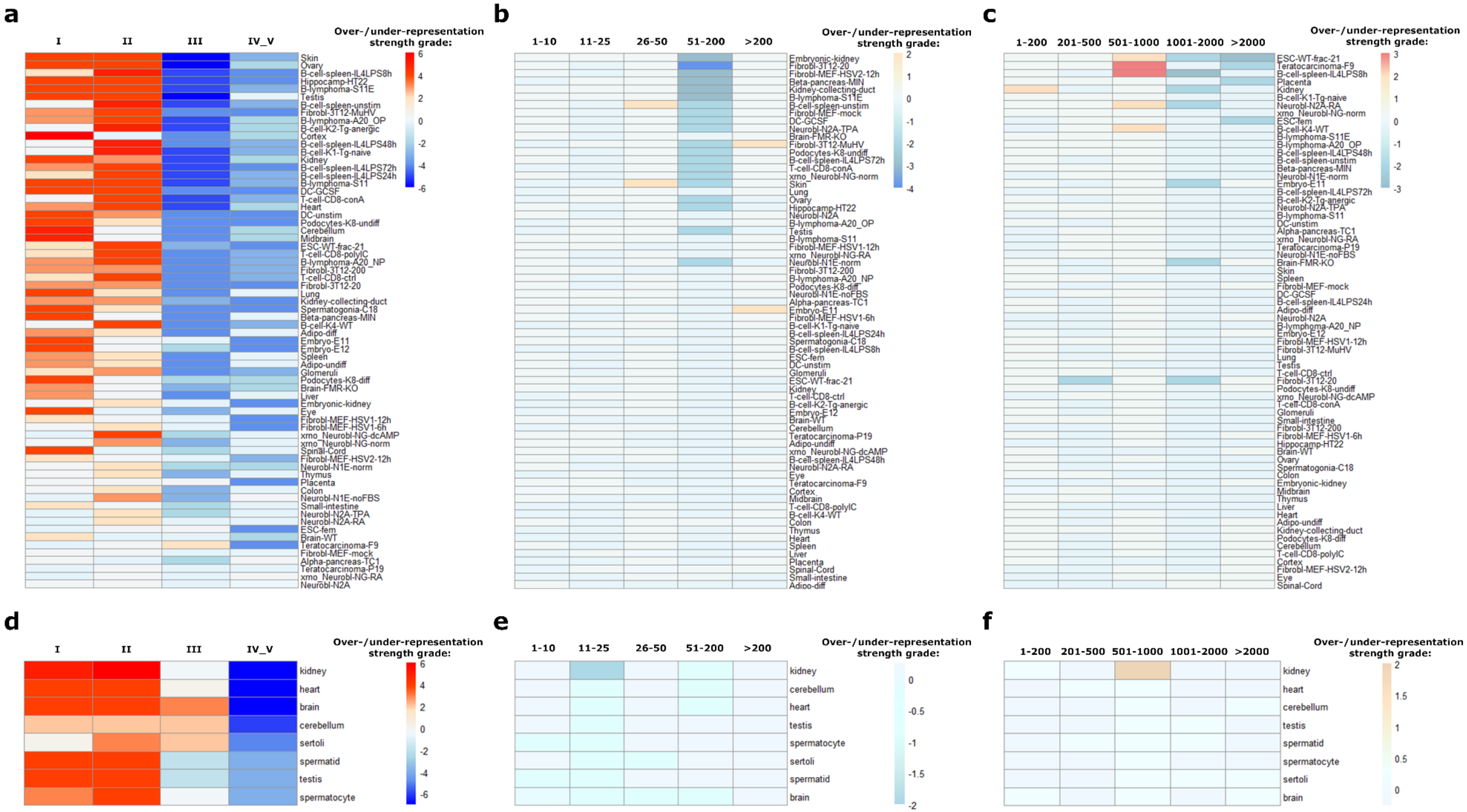

a
I
II
III
IV_V
Over-/under-representation strength grade:
6
4
2
0
-2
-4
-6
Skin
Ovary
B-cell-spleen-IL4LPS8h
Hippocamp-HT22
B-lymphoma-S11E
Testis
B-cell-spleen-unstim
Fibrobl-3T12-MuHV
B-lymphoma-A20_OP
B-cell-K2-Tg-anergic
Cortex
B-cell-spleen-IL4LPS48h
B-cell-K1-Tg-naive
Kidney
B-cell-spleen-IL4LPS72h
B-cell-spleen-IL4LPS24h
B-lymphoma-S11
DC-GCSF
T-cell-CD8-conA
Heart
DC-unstim
Podocytes-K8-undiff
Cerebellum
Midbrain
ESC-WT-frac-21
T-cell-CD8-polyIC
B-lymphoma-A20_NP
Fibrobl-3T12-200
T-cell-CD8-ctrl
Fibrobl-3T12-20
Lung
Kidney-collecting-duct
Spermatogonia-C18
Beta-pancreas-MIN
B-cell-K4-WT
Adipo-diff
Embryo-E11
Embryo-E12
Spleen
Adipo-undiff
Glomeruli
Podocytes-K8-diff
Brain-FMR-KO
Liver
Embryonic-kidney
Eye
Fibrobl-MEF-HSV1-12h
Fibrobl-MEF-HSV1-6h
xrno_Neurobl-NG-dcAMP
xrno_Neurobl-NG-norm
Spinal-Cord
Fibrobl-MEF-HSV2-12h
Neurobl-N1E-norm
Thymus
Placenta
Colon
Neurobl-N1E-noFBS
Small-intestine
Neurobl-N2A-TPA
Neurobl-N2A-RA
ESC-fem
Brain-WT
Teratocarcinoma-F9
Fibrobl-MEF-mock
Alpha-pancreas-TC1
Teratocarcinoma-P19
xrno_Neurobl-NG-RA
Neurobl-N2A
b
1-10
11-25
26-50
51-200
>200
Over-/under-representation strength grade:
2
1
0
-1
-2
-3
-4
Embryonic-kidney
Fibrobl-3T12-20
Fibrobl-MEF-HSV2-12h
Beta-pancreas-MIN
Kidney-collecting-duct
B-lymphoma-S11E
B-cell-spleen-unstim
Fibrobl-MEF-mock
DC-GCSF
Neurobl-N2A-TPA
Brain-FMR-KO
Fibrobl-3T12-MuHV
Podocytes-K8-undiff
B-cell-spleen-IL4LPS72h
T-cell-CD8-conA
xrno_Neurobl-NG-norm
Skin
Lung
Ovary
Hippocamp-HT22
Neurobl-N2A
B-lymphoma-A20_OP
Testis
B-lymphoma-S11
Fibrobl-MEF-HSV1-12h
xrno_Neurobl-NG-RA
Neurobl-N1E-norm
Fibrobl-3T12-200
B-lymphoma-A20_NP
Podocytes-K8-diff
Neurobl-N1E-noFBS
Alpha-pancreas-TC1
Embryo-E11
Fibrobl-MEF-HSV1-6h
B-cell-K1-Tg-naive
B-cell-spleen-IL4LPS24h
Spermatogonia-C18
B-cell-spleen-IL4LPS8h
ESC-fem
DC-unstim
Glomeruli
ESC-WT-frac-21
Kidney
T-cell-CD8-ctrl
B-cell-K2-Tg-anergic
Embryo-E12
Brain-WT
Cerebellum
Teratocarcinoma-P19
Adipo-undiff
xrno_Neurobl-NG-dcAMP
B-cell-spleen-IL4LPS48h
Neurobl-N2A-RA
Eye
Teratocarcinoma-F9
Cortex
Midbrain
T-cell-CD8-polyIC
B-cell-K4-WT
Colon
Thymus
Heart
Spleen
Liver
Placenta
Spinal-Cord
Small-intestine
Adipo-diff
c
1-200
201-500
501-1000
1001-2000
>2000
Over-/under-representation strength grade:
3
2
1
0
-1
-2
-3
ESC-WT-frac-21
Teratocarcinoma-F9
B-cell-spleen-IL4LPS8h
Placenta
Kidney
B-cell-K1-Tg-naive
Neurobl-N2A-RA
xrno_Neurobl-NG-norm
ESC-fem
B-cell-K4-WT
B-lymphoma-S11E
B-lymphoma-A20_OP
B-cell-spleen-IL4LPS48h
B-cell-spleen-unstim
Beta-pancreas-MIN
Neurobl-N1E-norm
Embryo-E11
B-cell-spleen-IL4LPS72h
B-cell-K2-Tg-anergic
Neurobl-N2A-TPA
B-lymphoma-S11
DC-unstim
Alpha-pancreas-TC1
xrno_Neurobl-NG-RA
Teratocarcinoma-P19
Neurobl-N1E-noFBS
Brain-FMR-KO
Skin
Spleen
Fibrobl-MEF-mock
DC-GCSF
B-cell-spleen-IL4LPS24h
Adipo-diff
Neurobl-N2A
B-lymphoma-A20_NP
Embryo-E12
Fibrobl-MEF-HSV1-12h
Fibrobl-3T12-MuHV
Lung
Testis
T-cell-CD8-ctrl
Fibrobl-3T12-20
Podocytes-K8-undiff
xrno_Neurobl-NG-dcAMP
T-cell-CD8-conA
Glomeruli
Small-intestine
Fibrobl-3T12-200
Fibrobl-MEF-HSV1-6h
Hippocamp-HT22
Brain-WT
Ovary
Spermatogonia-C18
Colon
Embryonic-kidney
Midbrain
Thymus
Liver
Heart
Adipo-undiff
Kidney-collecting-duct
Podocytes-K8-diff
Cerebellum
T-cell-CD8-polyIC
Cortex
Fibrobl-MEF-HSV2-12h
Eye
Spinal-Cord
d
I
II
III
IV_V
Over-/under-representation strength grade:
6
4
2
0
-2
-4
-6
kidney
heart
brain
cerebellum
sertoli
spermatid
testis
spermatocyte
e
1-10
11-25
26-50
51-200
>200
Over-/under-representation strength grade:
0
-0.5
-1
-1.5
-2
kidney
cerebellum
heart
testis
spermatocyte
sertoli
spermatid
brain
f
1-200
201-500
501-1000
1001-2000
>2000
Over-/under-representation strength grade:
2
1.5
1
0.5
0
kidney
heart
cerebellum
testis
spermatid
spermatocyte
sertoli
brain

Figure S13

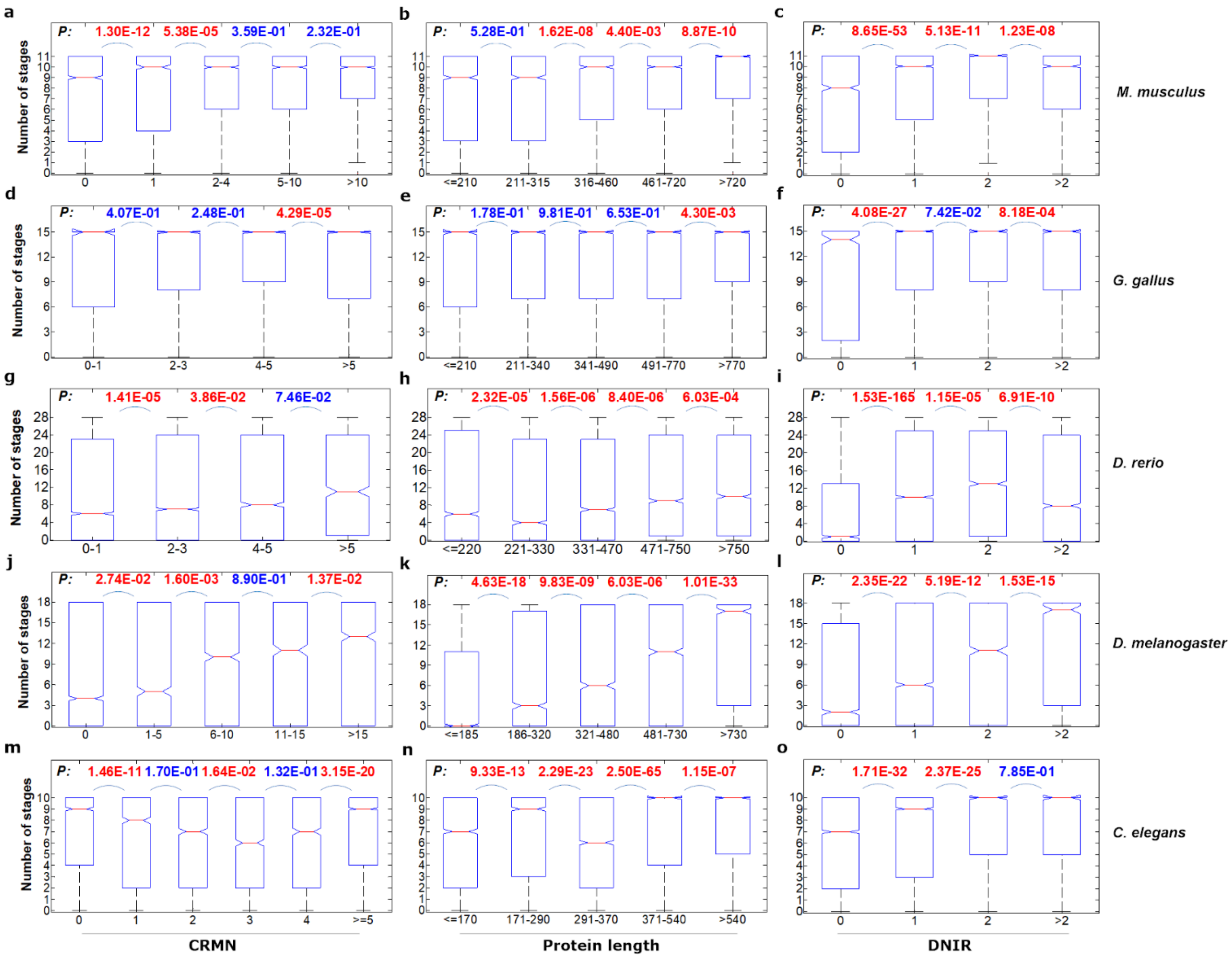
a
b
c
d
e
f
g
h
i
j
k
l
m
n
o
P:
1.30E-12
5.38E-05
3.59E-01
2.32E-01
5.28E-01
1.62E-08
4.40E-03
8.87E-10
8.65E-53
5.13E-11
1.23E-08
4.07E-01
2.48E-01
4.29E-05
1.78E-01
9.81E-01
6.53E-01
4.30E-03
4.08E-27
7.42E-02
8.18E-04
1.41E-05
3.86E-02
7.46E-02
2.32E-05
1.56E-06
8.40E-06
6.03E-04
1.53E-165
1.15E-05
6.91E-10
2.74E-02
1.60E-03
8.90E-01
1.37E-02
4.63E-18
9.83E-09
6.03E-06
1.01E-33
2.35E-22
5.19E-12
1.53E-15
1.46E-11
1.70E-01
1.64E-02
1.32E-01
3.15E-20
9.33E-13
2.29E-23
2.50E-65
1.15E-07
1.71E-32
2.37E-25
7.85E-01
Number of stages
M. musculus
G. gallus
D. rerio
D. melanogaster
C. elegans
CRMN
Protein length
DNIR

Figure 14

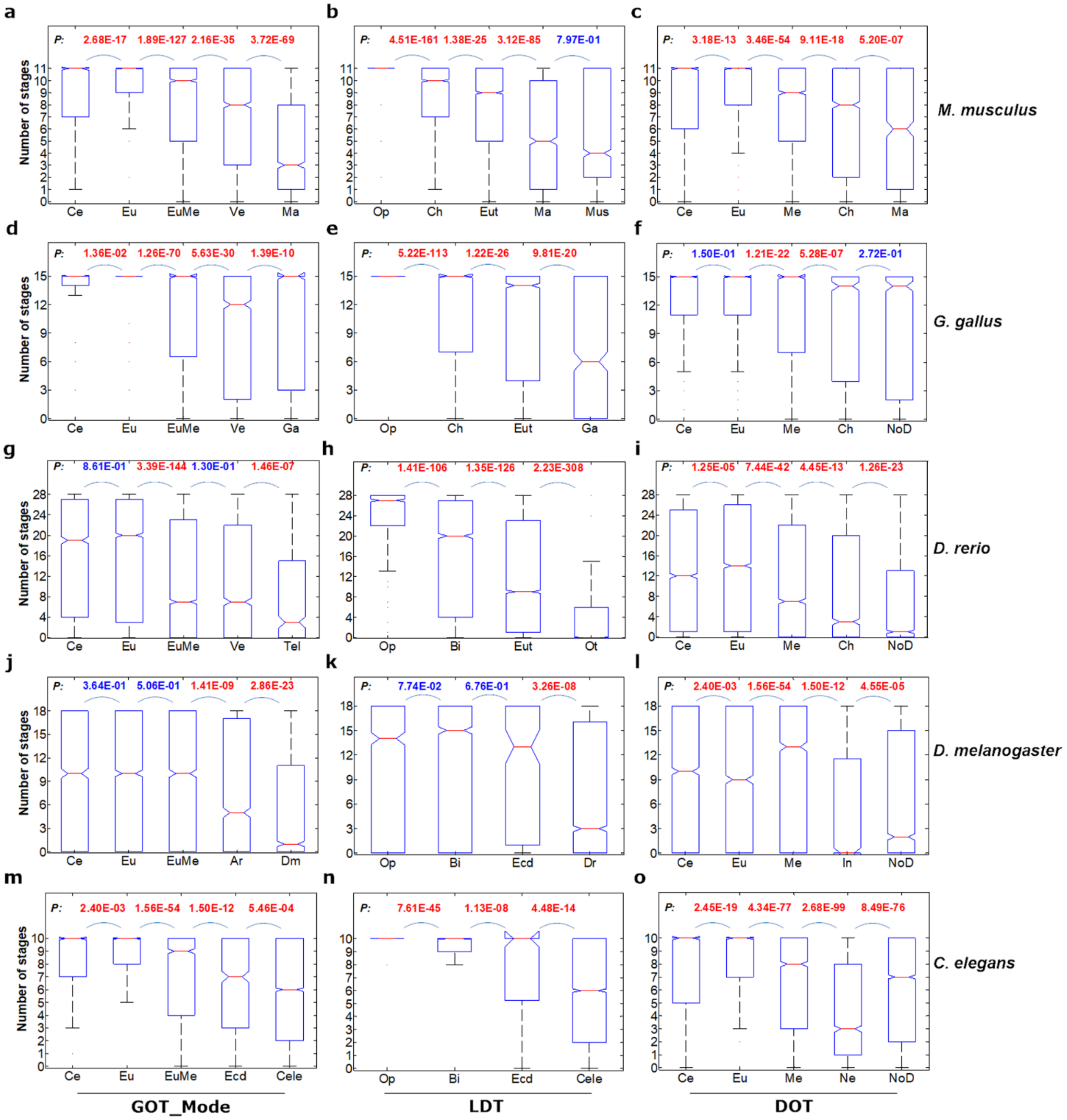
a
P: 2.68E-17 1.89E-127 2.16E-35 3.72E-69
Number of stages
Ce Eu EuMe Ve Ma
b
P: 4.51E-161 1.38E-25 3.12E-85 7.97E-01
Op Ch Eut Ma Mus
c
P: 3.18E-13 3.46E-54 9.11E-18 5.20E-07
Ce Eu Me Ch Ma
M. musculus
d
P: 1.36E-02 1.26E-70 5.63E-30 1.39E-10
Ce Eu EuMe Ve Ga
e
P: 5.22E-113 1.22E-26 9.81E-20
Op Ch Eut Ga
f
P: 1.50E-01 1.21E-22 5.28E-07 2.72E-01
Ce Eu Me Ch NoD
G. gallus
g
P: 8.61E-01 3.39E-144 1.30E-01 1.46E-07
Ce Eu EuMe Ve Tel
h
P: 1.41E-106 1.35E-126 2.23E-308
Op Bi Eut Ot
i
P: 1.25E-05 7.44E-42 4.45E-13 1.26E-23
Ce Eu Me Ch NoD
D. rerio
j
P: 3.64E-01 5.06E-01 1.41E-09 2.86E-23
Ce Eu EuMe Ar Dm
k
P: 7.74E-02 6.76E-01 3.26E-08
Op Bi Ecd Dr
l
P: 2.40E-03 1.56E-54 1.50E-12 4.55E-05
Ce Eu Me In NoD
D. melanogaster
m
P: 2.40E-03 1.56E-54 1.50E-12 5.46E-04
Ce Eu EuMe Ecd Cele
n
P: 7.61E-45 1.13E-08 4.48E-14
Op Bi Ecd Cele
o
P: 2.45E-19 4.34E-77 2.68E-99 8.49E-76
Ce Eu Me Ne NoD
C. elegans
GOT_Mode
LDT
DOT

Figure S15

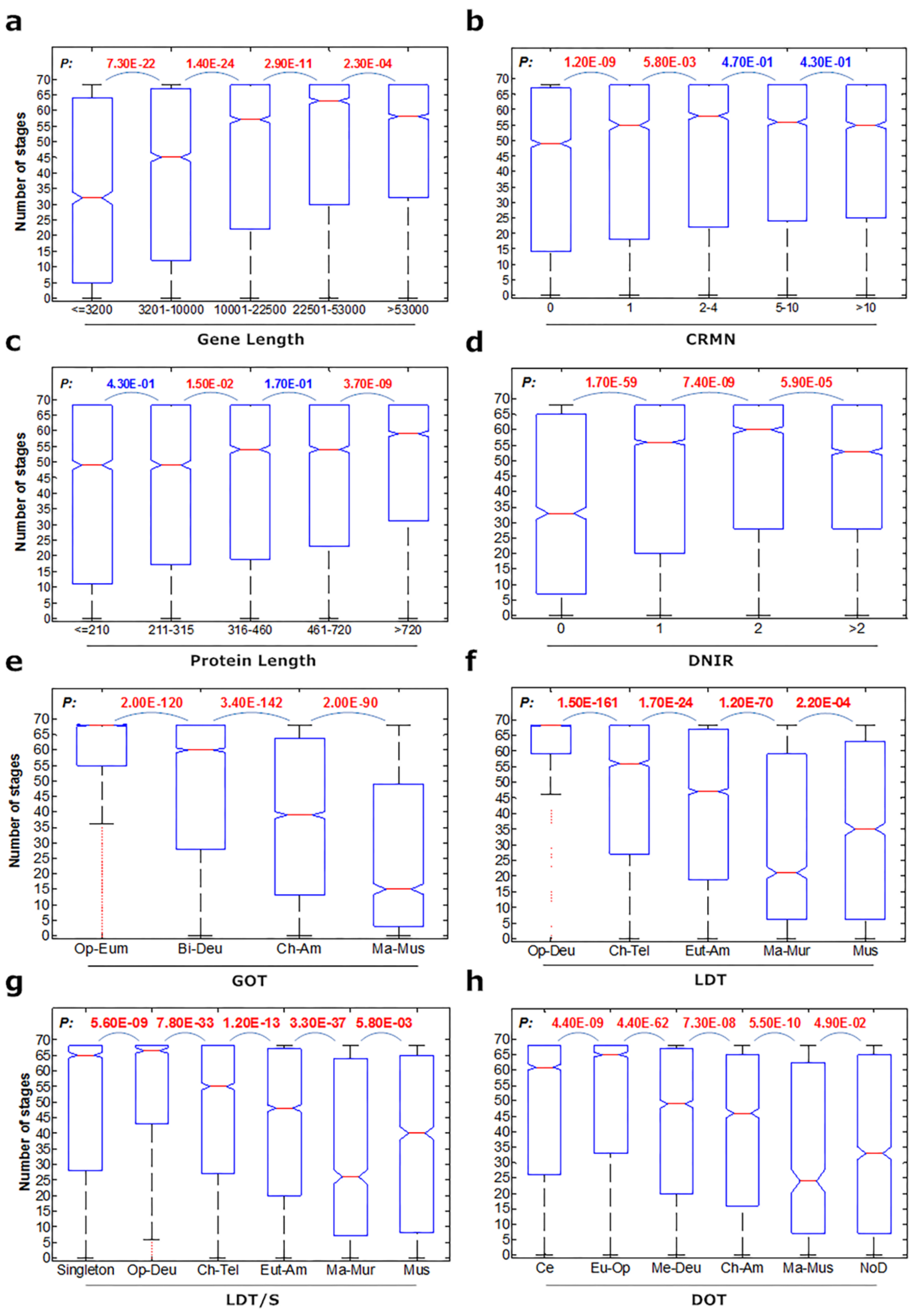
a
P: 7.30E-22 1.40E-24 2.90E-11 2.30E-04
Number of stages
<=3200 3201-10000 10001-22500 22501-53000 >53000
Gene Length
b
P: 1.20E-09 5.80E-03 4.70E-01 4.30E-01
0 1 2-4 5-10 >10
CRMN
c
P: 4.30E-01 1.50E-02 1.70E-01 3.70E-09
Number of stages
<=210 211-315 316-460 461-720 >720
Protein Length
d
P: 1.70E-59 7.40E-09 5.90E-05
0 1 2 >2
DNIR
e
P: 2.00E-120 3.40E-142 2.00E-90
Number of stages
Op-Eum Bi-Deu Ch-Am Ma-Mus
GOT
f
P: 1.50E-161 1.70E-24 1.20E-70 2.20E-04
Op-Deu Ch-Tel Eut-Am Ma-Mur Mus
LDT
g
P: 5.60E-09 7.80E-33 1.20E-13 3.30E-37 5.80E-03
Number of stages
Singleton Op-Deu Ch-Tel Eut-Am Ma-Mur Mus
LDT/S
h
P: 4.40E-09 4.40E-62 7.30E-08 5.50E-10 4.90E-02
Ce Eu-Op Me-Deu Ch-Am Ma-Mus NoD
DOT

Figure S16

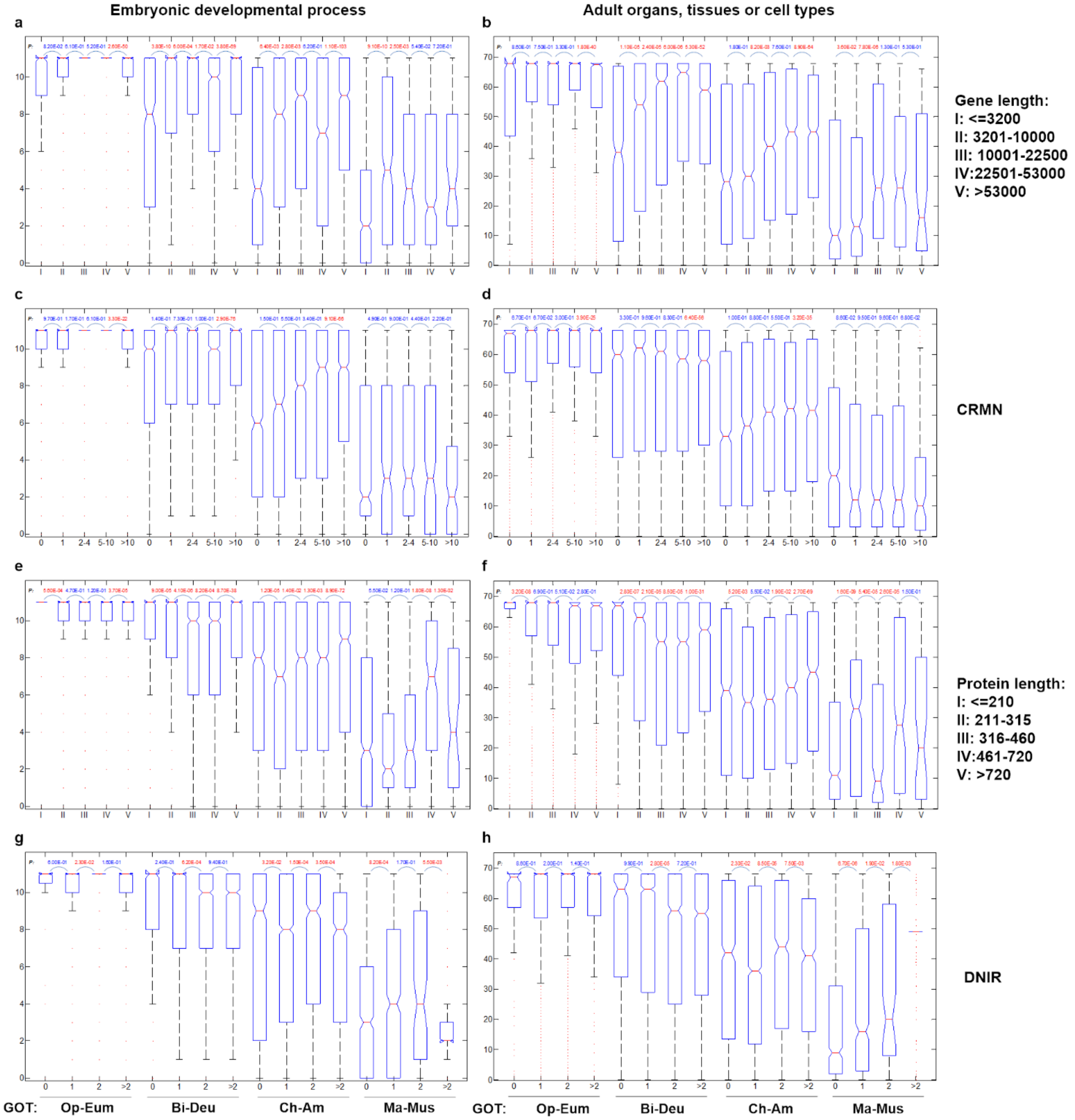
Embryonic developmental process
Adult organs, tissues or cell types
a
b
c
d
e
f
g
h
Gene length:
I: <=3200
II: 3201-10000
III: 10001-22500
IV:22501-53000
V: >53000
CRMN
Protein length:
I: <=210
II: 211-315
III: 316-460
IV:461-720
V: >720
DNIR
GOT:
Op-Eum
Bi-Deu
Ch-Am
Ma-Mus

Figure S17

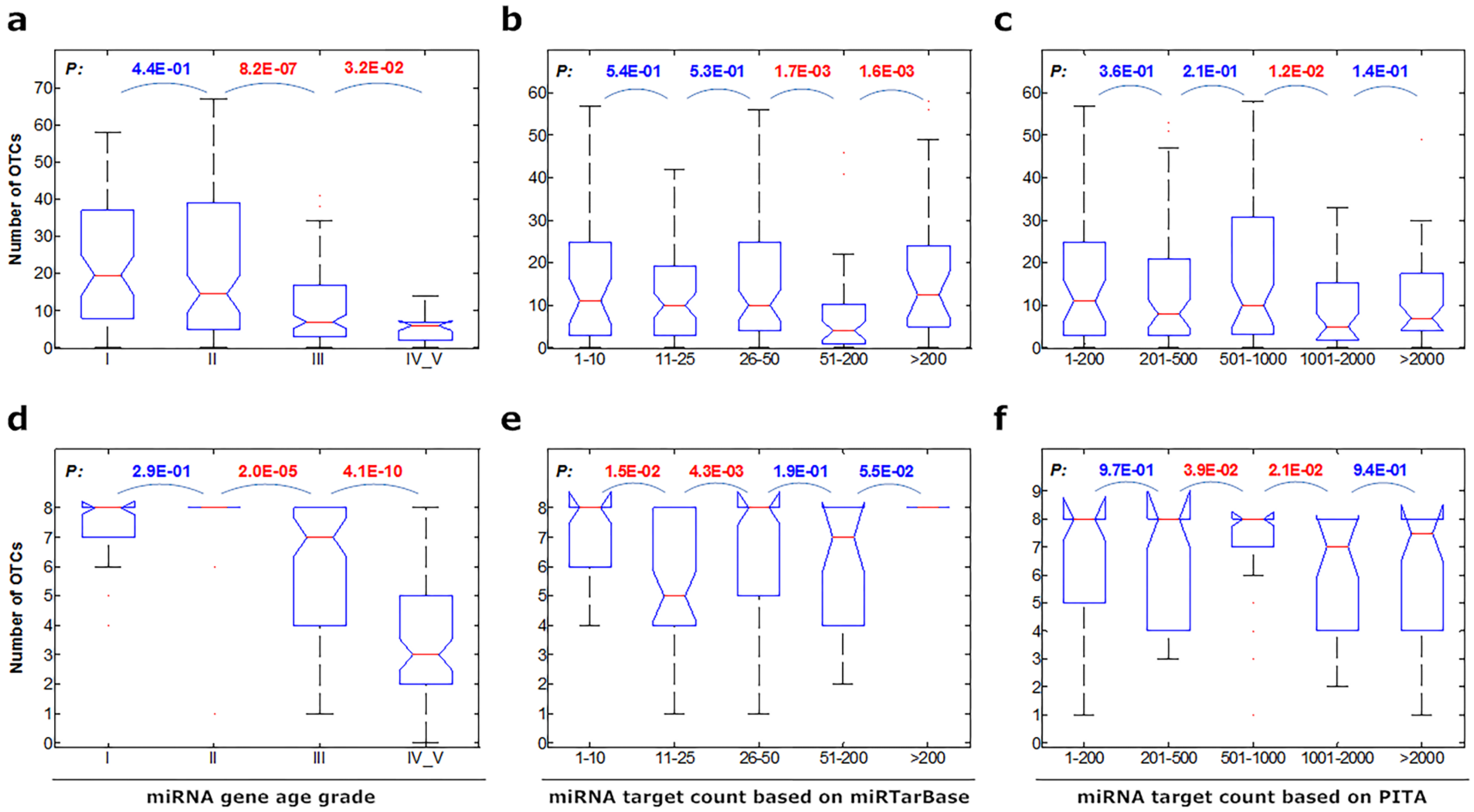
a
b
c
d
e
f
P:
4.4E-01
8.2E-07
3.2E-02
5.4E-01
5.3E-01
1.7E-03
1.6E-03
3.6E-01
2.1E-01
1.2E-02
1.4E-01
2.9E-01
2.0E-05
4.1E-10
1.5E-02
4.3E-03
1.9E-01
5.5E-02
9.7E-01
3.9E-02
2.1E-02
9.4E-01
Number of OTCs
I
II
III
IV_V
1-10
11-25
26-50
51-200
>200
1-200
201-500
501-1000
1001-2000
>2000
miRNA gene age grade
miRNA target count based on miRTarBase
miRNA target count based on PITA

Figure S18

a

Gene length

	<=3200	3201-10000	10001-22500	22501-53000	>53000
WE	403	873	1204	1418	1184
Other	1370	2562	2641	2552	2928
SOTCs	430	384	233	175	155

CRMN

	0	1	2-4	5-10	>10
WE	1023	682	1313	1032	1032
Other	3134	1533	2767	2314	2305
SOTCs	513	205	264	213	182

Protein length

	<=210	211-315	316-460	461-720	>720
WE	976	776	1014	1092	1224
Other	1982	2027	2477	2848	2719
SOTCs	377	299	270	252	179

DNIR

	0	1	2	>2
WE	351	2737	1048	946
Other	1438	5966	2137	2512
SOTCs	351	692	159	175

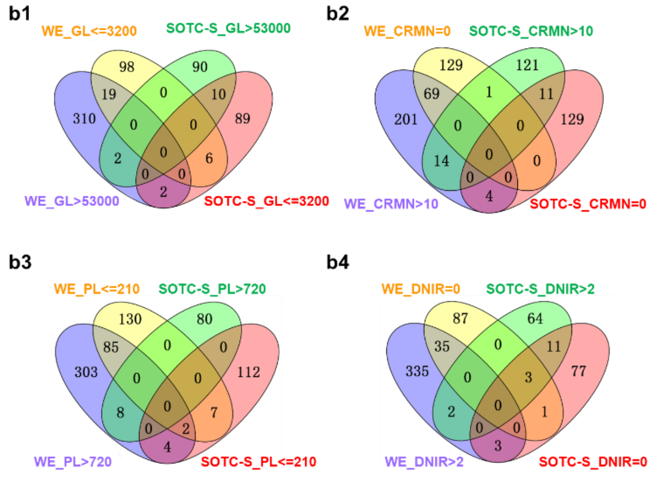

c

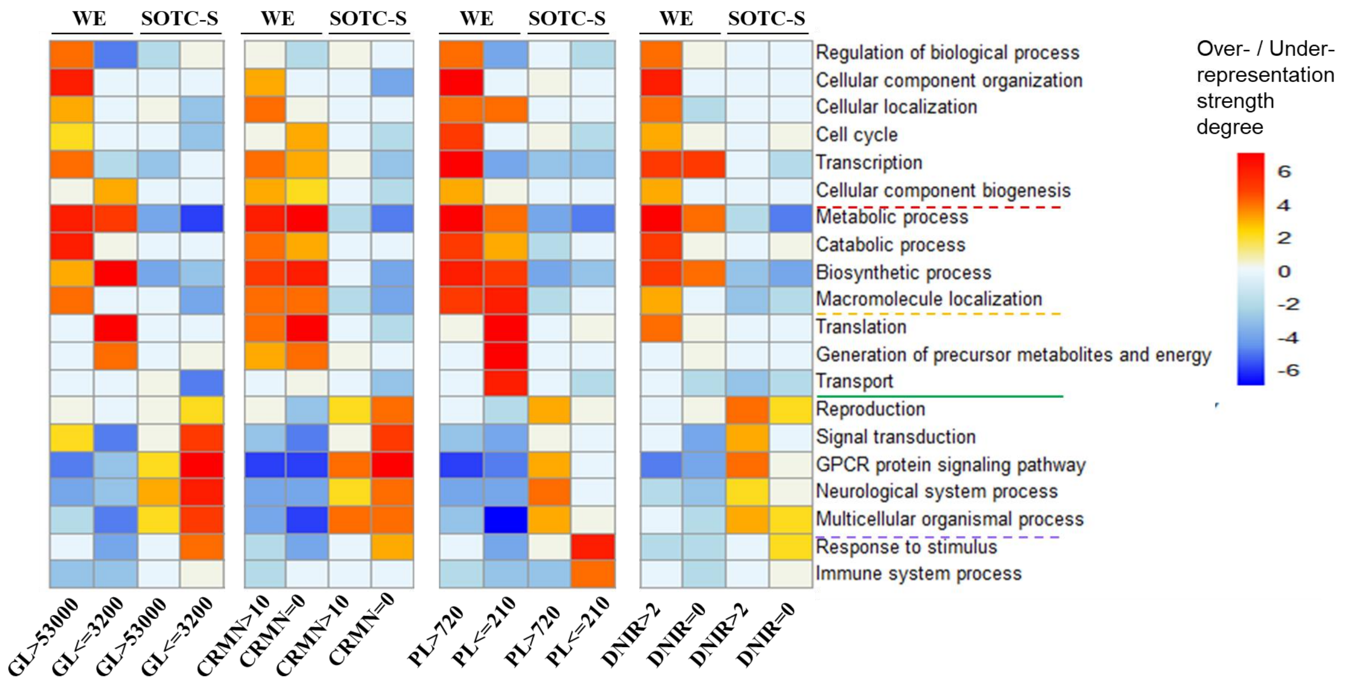

d1

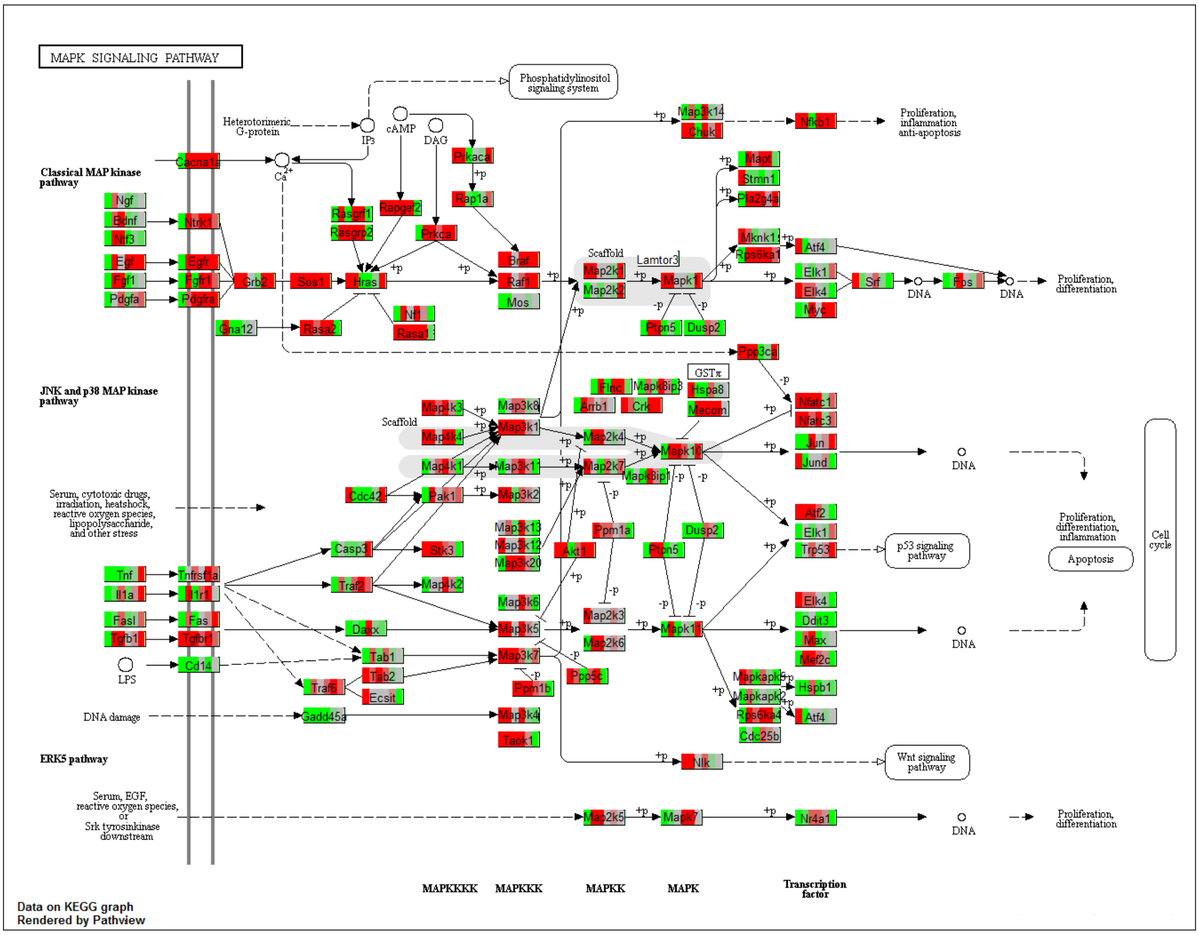

Column:	1	2	3	4	5	6
	HK/SOTC-S	GL	CRMN	PL	DNIR	Pathway_count
	HK	>53000	>10	>720	>2	>10
	----	22500-53000	5-10	461-720	2	5-10
	No data	10001-22500	2-4	316-460	----	3-4
	----	3201-10000	1	211-315	1	2
	SOTC-S	<=3200	0	<=210	0	1

d2

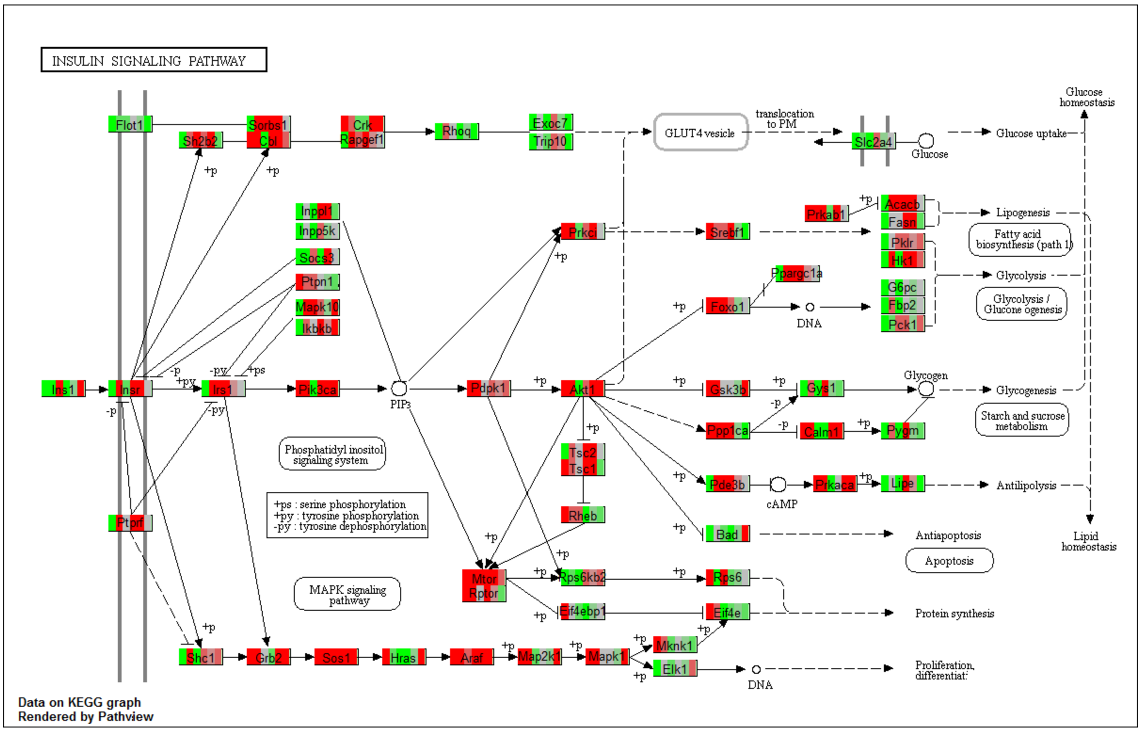

INSULIN SIGNALING PATHWAY
Flot1
Sorbs1
Cbl
Sh2b2
Crk
Rapgef1
Rhoq
Exoc7
Trip10
GLUT4 vesicle
translocation to PM
Slc2a4
Glucose
Glucose uptake
Glucose homeostasis
Inpp1
Inpp5k
Socs3
Ptpn1
Mapk10
Ikbkb
Prkci
Srebf1
Prkab1
Acacb
Fasn
Pklr
Hk1
Lipogenesis
Fatty acid biosynthesis (path 1)
Ppargc1a
Foxo1
DNA
G6pc
Fbp2
Pck1
Glycolysis
Glycolysis / Gluconeogenesis
Ins1
Insr
Irs1
Pik3ca
PIP3
Pdpk1
Akt1
Gsk3b
Gys1
Glycogen
Glycogenesis
Ppp1ca
Calm1
Pygm
Starch and sucrose metabolism
Tsc2
Tsc1
Pde3b
cAMP
Prkaca
Lipe
Antilipolysis
Lipid homeostasis
Rheb
Bad
Antiapoptosis
Apoptosis
Ptprf
Phosphatidyl inositol signaling system
+ps : serine phosphorylation
+py : tyrosine phosphorylation
-py : tyrosine dephosphorylation
MAPK signaling pathway
Mtor
Rptor
Rps6kb2
Rps6
Eif4ebp1
Eif4e
Protein synthesis
Shc1
Grb2
Sos1
Hras
Araf
Map2k1
Mapk1
Mknk1
Elk1
Proliferation, differentiat:
Data on KEGG graph
Rendered by Pathview

d3

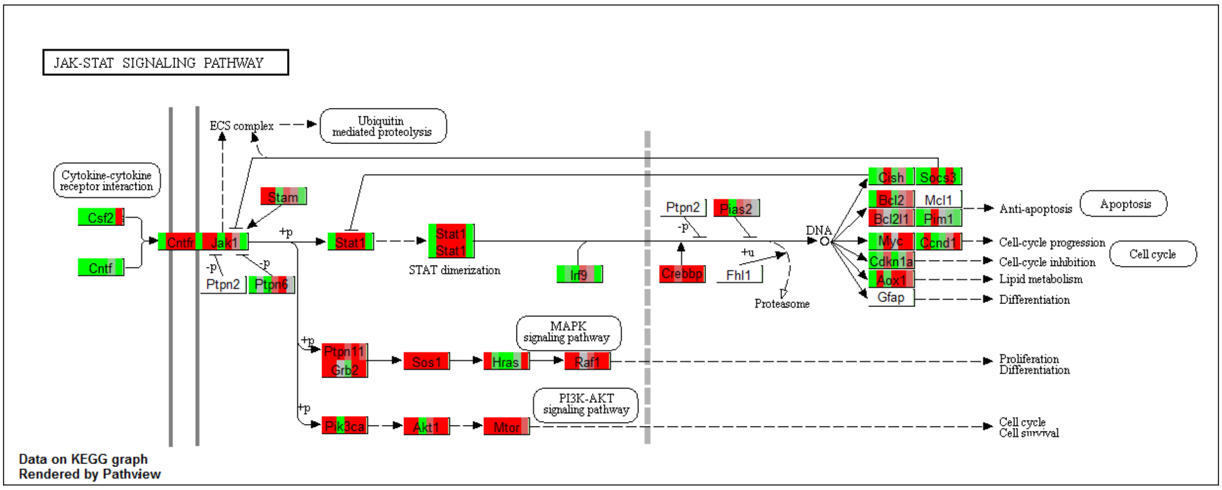
JAK-STAT SIGNALING PATHWAY
ECS complex
Ubiquitin mediated proteolysis
Cytokine-cytokine receptor interaction
Csf2
Cntf
Cntfr
Jak1
Stam
Stat1
Stat1
Stat1
STAT dimerization
Ptpn2
Ptpn6
Irf9
Ptpn2
Pias2
Crebbp
Fhl1
Proteasome
DNA
Cish
Socs3
Bcl2
Mcl1
Bcl2l1
Pim1
Myc
Ccnd1
Cdkn1a
Aox1
Gfap
Anti-apoptosis
Apoptosis
Cell-cycle progression
Cell cycle
Cell-cycle inhibition
Lipid metabolism
Differentiation
MAPK signaling pathway
Ptpn11
Grb2
Sos1
Hras
Raf1
Proliferation
Differentiation
PI3K-AKT signaling pathway
Pik3ca
Akt1
Mtor
Cell cycle
Cell survival
Data on KEGG graph
Rendered by Pathview

d4

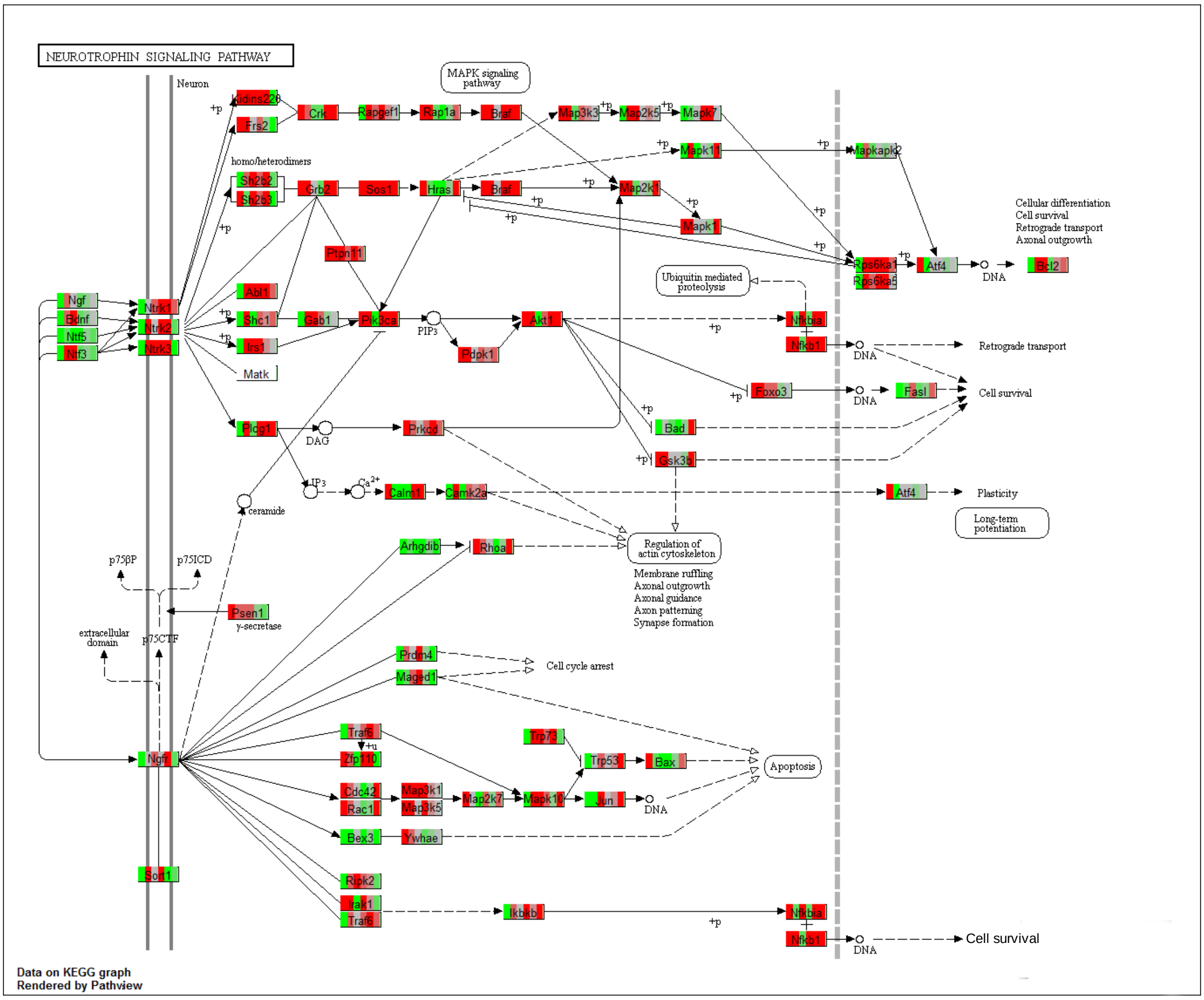
NEUROTROPHIN SIGNALING PATHWAY
Neuron
Kidins220
Frs2
Crk
Rapgef1
Rap1a
Braf
Map3k3
Map2k5
Mapk7
MAPK signaling pathway
Mapk11
Mapkapk2
homo/heterodimers
Sh2b2
Sh2b3
Grb2
Sos1
Hras
Braf
Map2k1
Mapk1
Ptpn11
Rps6ka1
Rps6ka5
Atf4
DNA
Bcl2
Cellular differentiation
Cell survival
Retrograde transport
Axonal outgrowth
Ubiquitin mediated proteolysis
Ngf
Bdnf
Ntf5
Ntf3
Ntrk1
Ntrk2
Ntrk3
Abl1
Shc1
Gab1
Pik3ca
PIP3
Akt1
Nfkbia
Nfkb1
Irs1
Pdpk1
Matk
Foxo3
Fasl
Retrograde transport
Cell survival
Plcg1
DAG
Prkcd
Bad
Gsk3b
IP3
Calm1
Camk2a
Atf4
Plasticity
Long-term potentiation
ceramide
Arhgdib
Rhoa
Regulation of actin cytoskeleton
Membrane ruffling
Axonal outgrowth
Axonal guidance
Axon patterning
Synapse formation
p75βP
p75ICD
Psen1
γ-secretase
extracellular domain
p75CTF
Prdm4
Maged1
Cell cycle arrest
Traf6
Zfp110
Trp73
Trp53
Bax
Apoptosis
Ngfr
Cdc42
Rac1
Map3k1
Map3k5
Map2k7
Mapk10
Jun
Bex3
Ywhae
Sort1
Ripk2
Irak1
Traf6
Ikbkb
Nfkbia
Nfkb1
Cell survival
Data on KEGG graph
Rendered by Pathview

d5

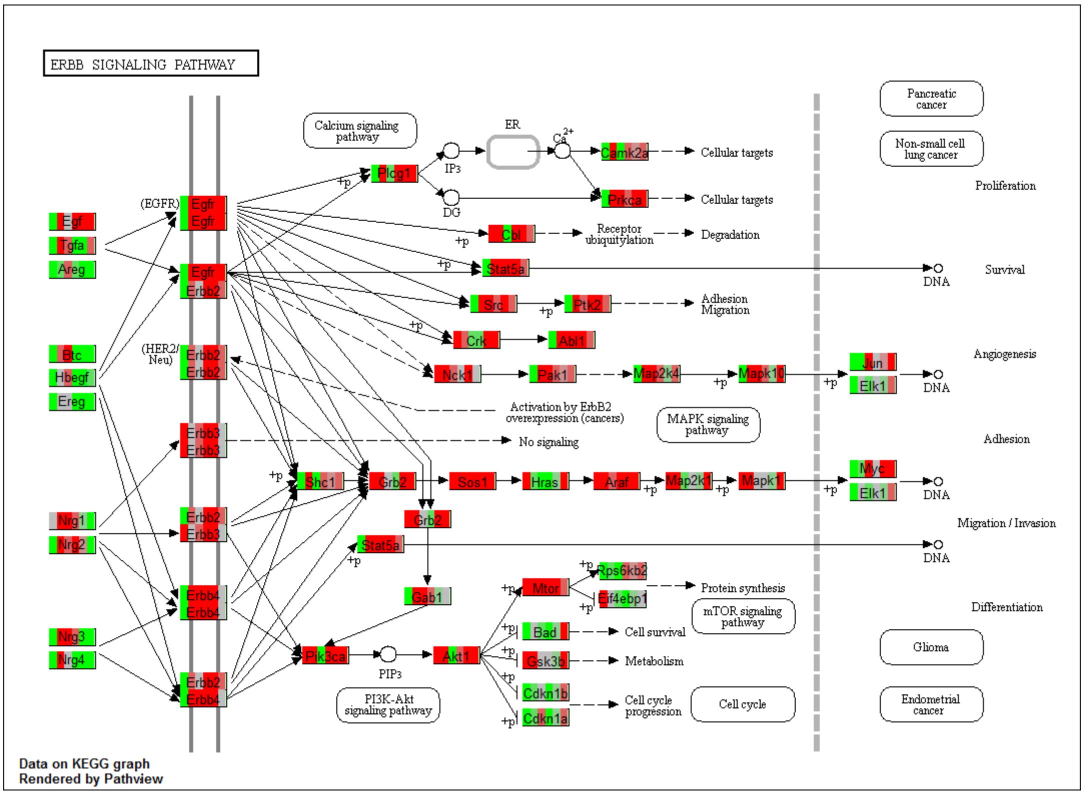
ERBB SIGNALING PATHWAY
Egf
Tgfa
Areg
Btc
Hbegf
Ereg
Nrg1
Nrg2
Nrg3
Nrg4
(EGFR)
(HER2/Neu)
Egfr
Erbb2
Erbb3
Erbb4
Calcium signaling pathway
ER
IP3
DG
Ca^{2+}
Plcg1
Camk2a
Prkca
Cellular targets
Cbl
Receptor ubiquitylation
Degradation
Stat5a
Src
Ptk2
Adhesion Migration
Crk
Abl1
Nck1
Pak1
Map2k4
Mapk10
Jun
Elk1
Activation by ErbB2 overexpression (cancers)
MAPK signaling pathway
No signaling
Shc1
Grb2
Sos1
Hras
Araf
Map2k1
Mapk1
Myc
Gab1
Mtor
Rps6kb2
Eif4ebp1
Protein synthesis
mTOR signaling pathway
Pik3ca
PIP3
Akt1
Bad
Gsk3b
Cdkn1b
Cdkn1a
Cell survival
Metabolism
Cell cycle progression
Cell cycle
PI3K-Akt signaling pathway
DNA
+p
Pancreatic cancer
Non-small cell lung cancer
Proliferation
Survival
Angiogenesis
Adhesion
Migration / Invasion
Differentiation
Glioma
Endometrial cancer
Data on KEGG graph
Rendered by Pathview

d6

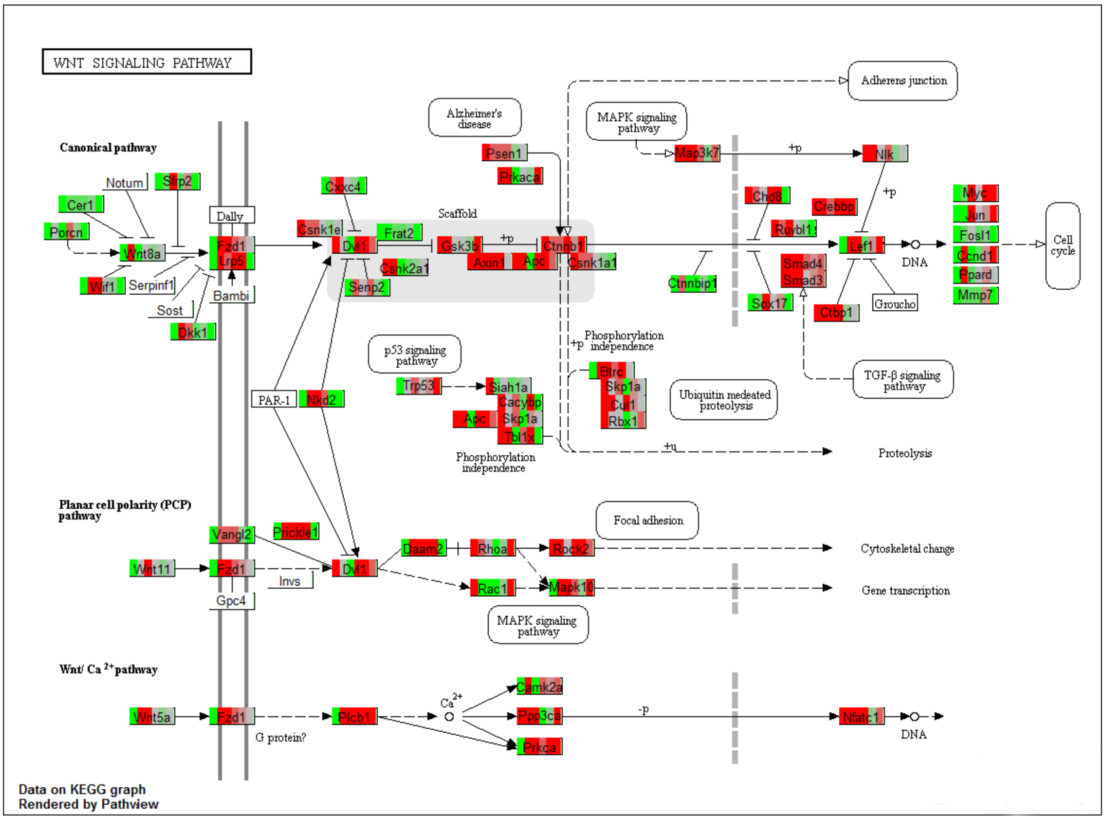
WNT SIGNALING PATHWAY
Canonical pathway
Notum
Sfrp2
Cer1
Porcn
Wnt8a
Wif1
Serpinf1
Sost
Dkk1
Dally
Fzd1
Lrp5
Bambi
Alzheimer's disease
Psen1
Prkaca
Cxxc4
Csnk1e
Dvl1
Frat2
Scaffold
Gsk3b
Axin1
Apc
Ctnnb1
Csnk1a1
Csnk2a1
Senp2
MAPK signaling pathway
Map3k7
Nlk
Adherens junction
Chd8
Crebbp
Ruvbl1
Smad4
Smad3
Sox17
Lef1
Ctbp1
Groucho
Ctnnbip1
DNA
Myc
Jun
Fosl1
Ccnd1
Ppard
Mmp7
Cell cycle
p53 signaling pathway
Trp53
Siah1a
Cacybp
Skp1a
Tbl1x
Phosphorylation independence
PAR-1
Nkd2
Btrc
Cul1
Rbx1
Ubiquitin mediated proteolysis
TGF-β signaling pathway
Proteolysis
+p
+u
Planar cell polarity (PCP) pathway
Vangl2
Prickle1
Wnt11
Invs
Gpc4
Daam2
Rhoa
Rock2
Rac1
Mapk10
Focal adhesion
Cytoskeletal change
Gene transcription
Wnt/ Ca^{2+} pathway
Wnt5a
Plcb1
G protein?
Ca^{2+}
Camk2a
Ppp3ca
Prkca
-p
Nfatc1
Data on KEGG graph
Rendered by Pathview

d7

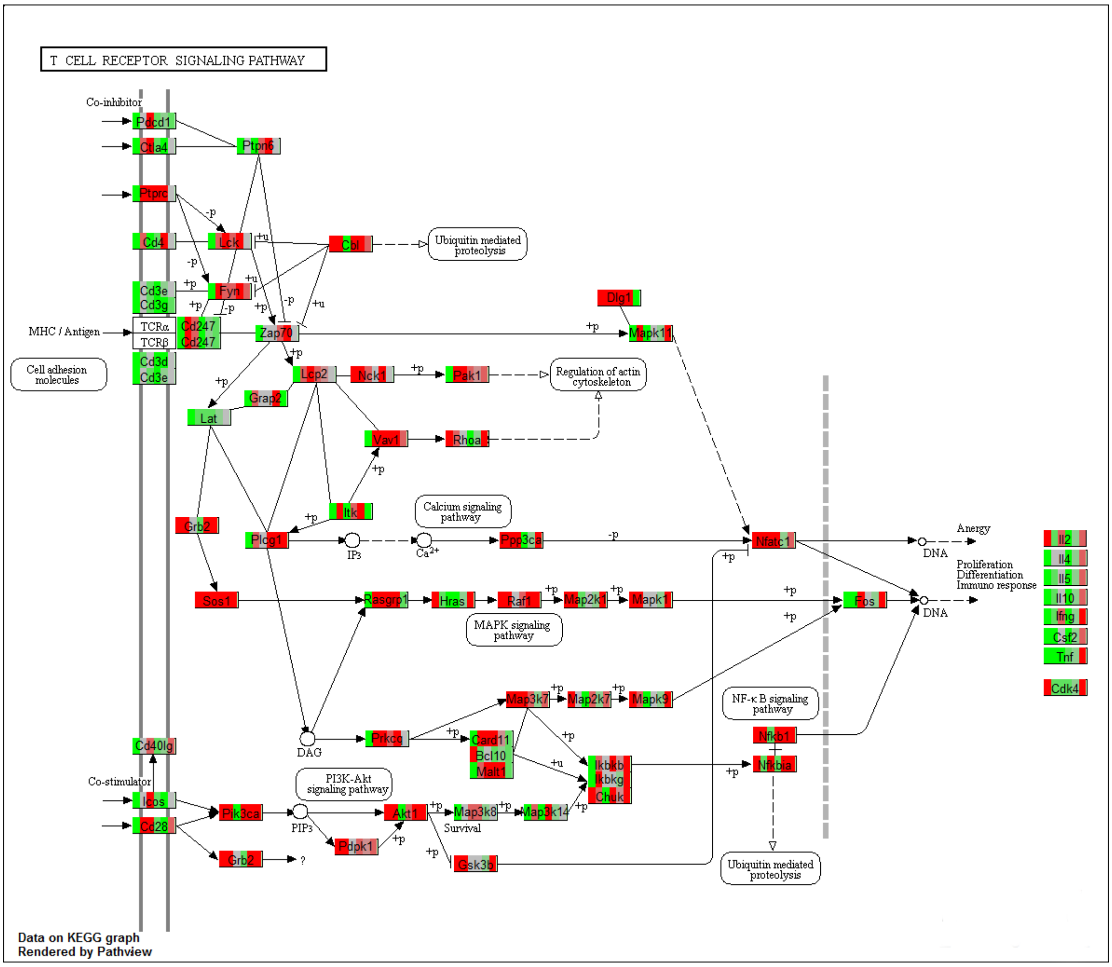
T CELL RECEPTOR SIGNALING PATHWAY
Data on KEGG graph
Rendered by Pathview

d8

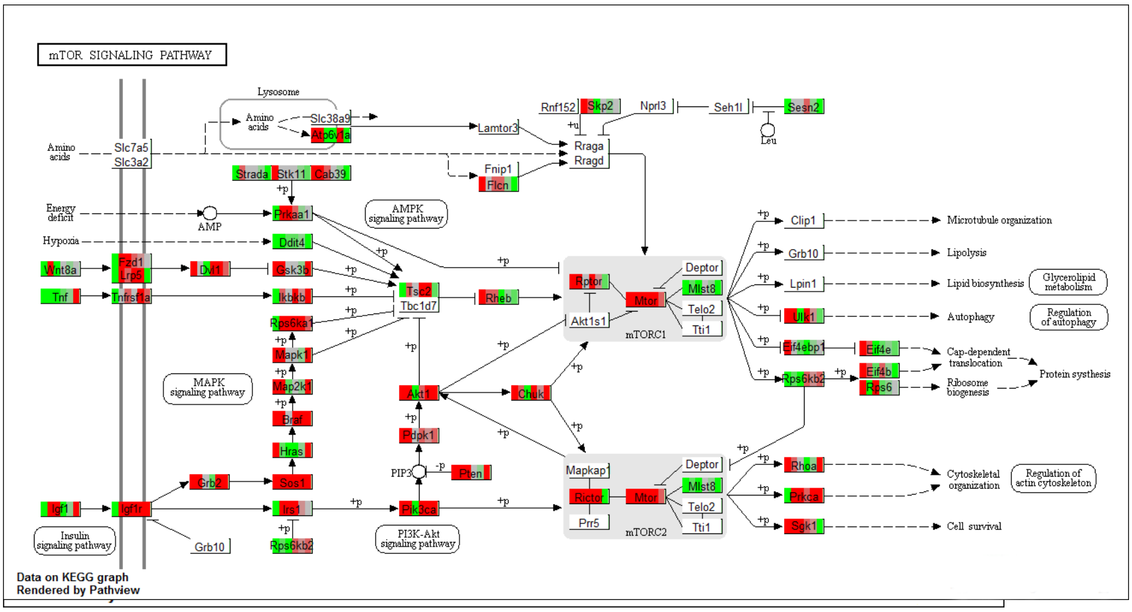
mTOR SIGNALING PATHWAY
Data on KEGG graph
Rendered by Pathview

d9

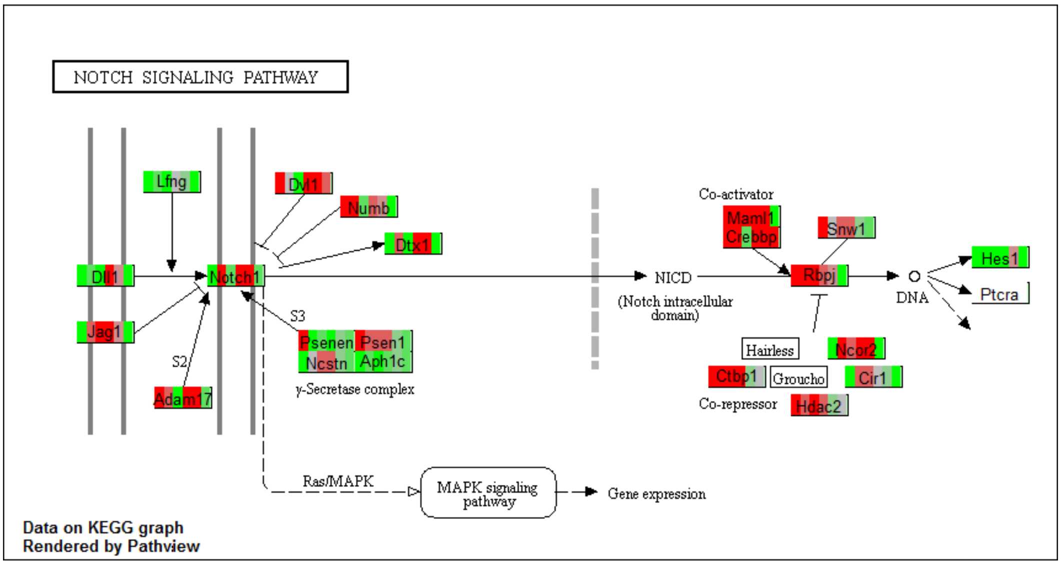
NOTCH SIGNALING PATHWAY
Lfng
Dll1
Jag1
Notch1
Adam17
S2
Dvl1
Numb
Dtx1
S3
Psenen
Psen1
Ncstn
Aph1c
γ-Secretase complex
NICD
(Notch intracellular domain)
Co-activator
Maml1
Crebbp
Snw1
Rbpj
DNA
Hes1
Ptcra
Hairless
Ncor2
Ctbp1
Groucho
Cir1
Co-repressor
Hdac2
Ras/MAPK
MAPK signaling pathway
Gene expression
Data on KEGG graph
Rendered by Pathview

Figure S19

a

GOT

	I	II	III	IV
WE	2043	2131	847	61
Other	1822	4500	4506	1225
SOTCs	104	304	522	447

LDT

	Op-Bi	Ch-Ve	Eut-Am	Ma-Mur	Mus
WE	2124	1101	1197	297	363
Other	1977	2869	4339	1382	1486
SOTCs	73	188	372	360	384

DOT

	Ce	Eu-Op	Me-Deu	Ch-Am	Ma-Mur
WE	1558	2002	703	389	79
Other	2940	2975	2382	1918	400
SOTCs	243	257	204	237	85

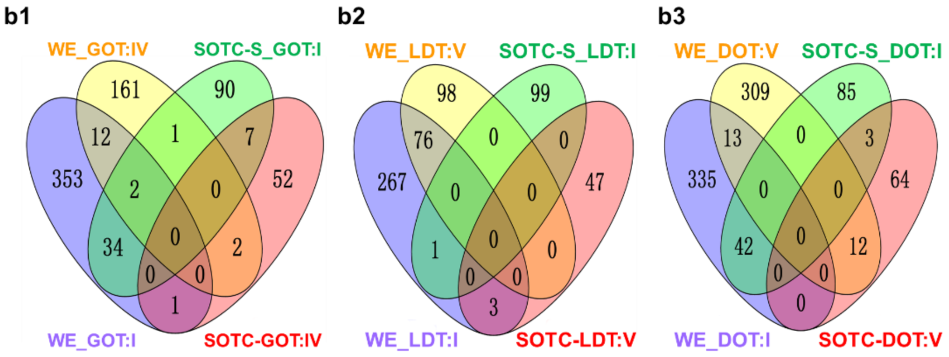

c

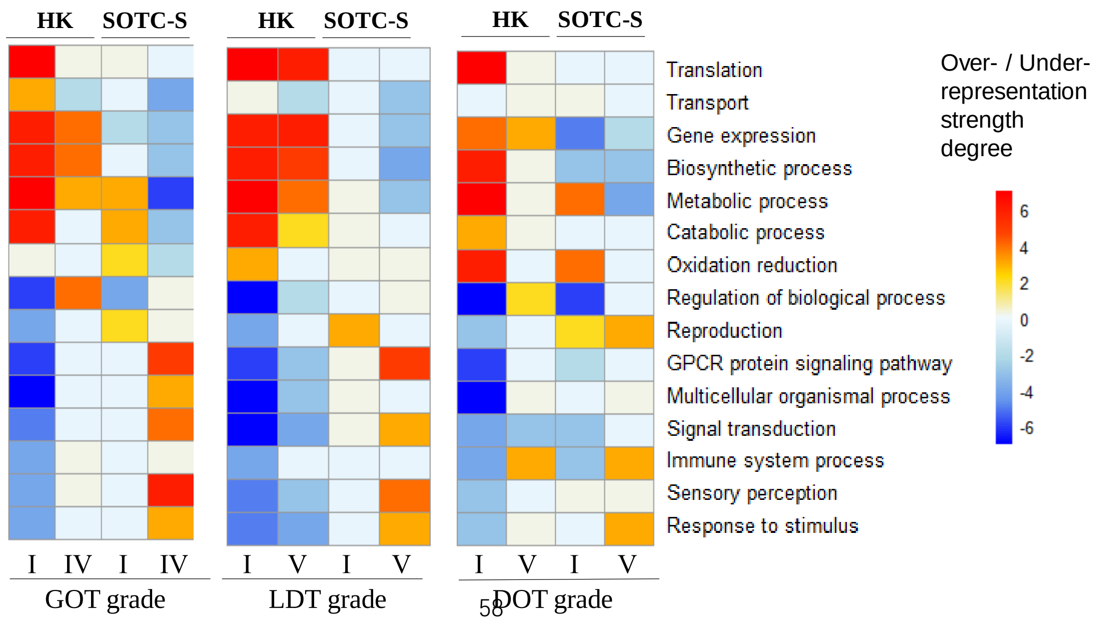

d

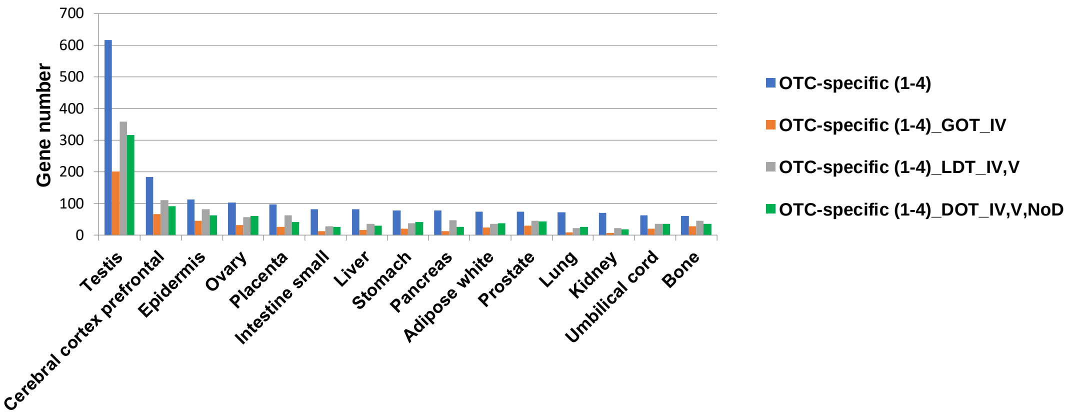
700
600
500
400
300
200
100
0
Gene number
Testis
Cerebral cortex prefrontal
Epidermis
Ovary
Placenta
Intestine small
Liver
Stomach
Pancreas
Adipose white
Prostate
Lung
Kidney
Umbilical cord
Bone
OTC-specific (1-4)
OTC-specific (1-4)_GOT_IV
OTC-specific (1-4)_LDT_IV,V
OTC-specific (1-4)_DOT_IV,V,NoD

e

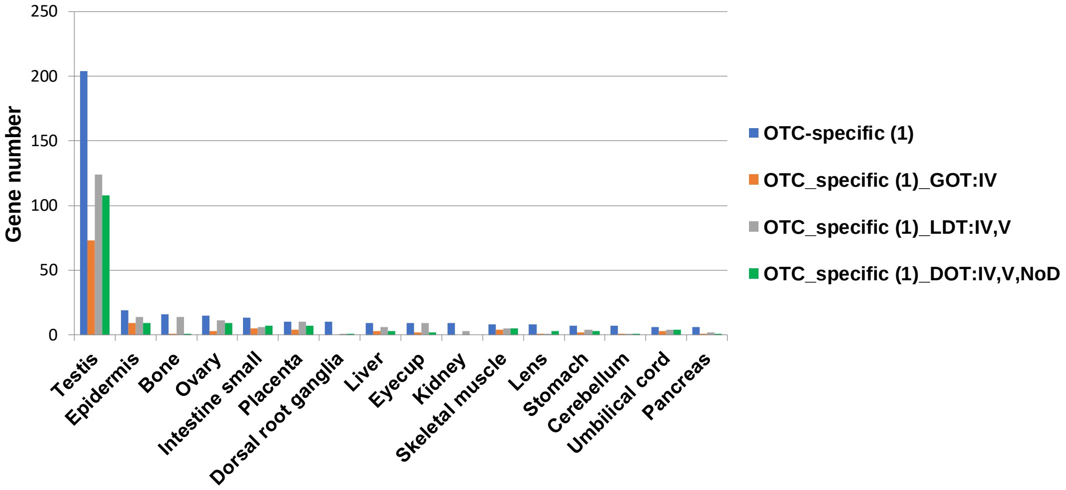
250
200
150
100
50
0
Gene number
Testis
Epidermis
Bone
Ovary
Intestine small
Placenta
Dorsal root ganglia
Liver
Eyecup
Kidney
Skeletal muscle
Lens
Stomach
Cerebellum
Umbilical cord
Pancreas
OTC-specific (1)
OTC_specific (1)_GOT:IV
OTC_specific (1)_LDT:IV,V
OTC_specific (1)_DOT:IV,V,NoD
